# Allele-specific gene editing to rescue dominant *CRX*-associated LCA7 phenotypes in a retinal organoid model

**DOI:** 10.1016/j.stemcr.2021.09.007

**Published:** 2021-10-14

**Authors:** Kathleen R. Chirco, Shereen Chew, Anthony T. Moore, Jacque L. Duncan, Deepak A. Lamba

**Affiliations:** 1 Department of Ophthalmology, University of California San Francisco, San Francisco, CA, USA; 2 The Eli and Edythe Broad Center of Regeneration Medicine and Stem Cell Research, University of California San Francisco, San Francisco, CA, USA; 3 Divison of Neuroscience, Oregon National Primate Research Center, Oregon Health & Science University, Beaverton, OR, USA; 4 Casey Eye Institute, Oregon Health & Science University, Portland, OR, USA

**Keywords:** Leber congenital amaurosis, LCA7, CRX, retinal organoid, photoreceptors cells, gene editing, CRISPR/Cas9, scRNA-seq, allelic knockdown

## Abstract

Cases of Leber congenital amaurosis caused by mutations in *CRX* (LCA7) exhibit an early form of the disease and show signs of significant photoreceptor dysfunction and eventual loss. To establish a translational *in vitro* model system to study gene-editing-based therapies, we generated LCA7 retinal organoids harboring a dominant disease-causing mutation in *CRX*. Our LCA7 retinal organoids develop signs of immature and dysfunctional photoreceptor cells, providing us with a reliable *in vitro* model to recapitulate LCA7. Furthermore, we performed a proof-of-concept study in which we utilize allele-specific CRISPR/Cas9-based gene editing to knock out mutant *CRX* and saw moderate rescue of photoreceptor phenotypes in our organoids. This work provides early evidence for an effective approach to treat LCA7, which can be applied more broadly to other dominant genetic diseases.

## Highlights

• A retinal organoid model of CRX-associated Leber congenital amaurosis was generated
• LCA7 retinal organoids show impaired photoreceptor maturation and gene expression
• scRNA-seq profiling revealed both rod and cone photoreceptor dysfunction
• Allele-specific editing of mutant CRX promotes disease rescue in retinal organoids

## Graphical abstract

**Figure:**
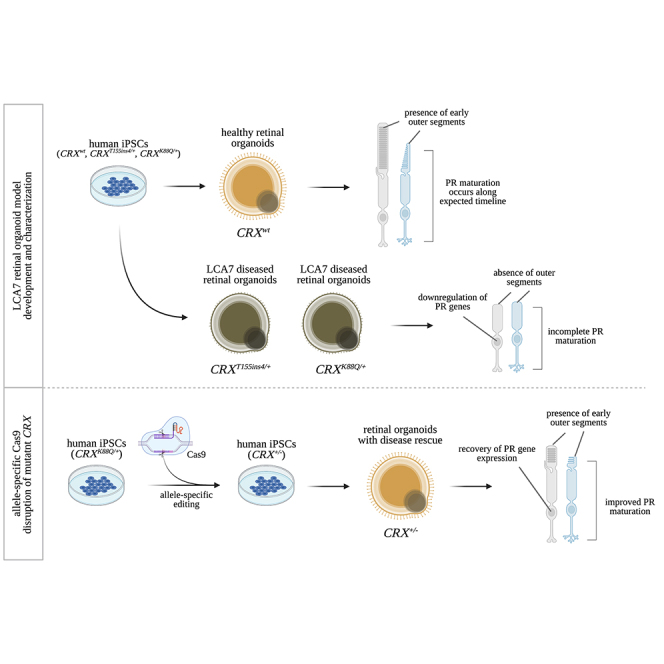

## Teaser

LCA7 currently has no effective treatments; therefore, Dr. Lamba and colleagues generated LCA7 retinal organoids from patient-derived stem cells. The retinal organoids recapitulated LCA7 disease phenotypes, allowing for testing of an allele-specific gene editing approach. Early signs of disease rescue were achieved, providing evidence for a promising new strategy to treat LCA7 and other dominant retinal diseases.

## Introduction

Leber congenital amaurosis (LCA) is a group of early-onset inherited retinal diseases that accounts for approximately 5% of all inherited retinopathies (Koenekoop, 2004; Stone, 2007). Although LCA patients can exhibit a range of disease severity, they commonly exhibit poor vision from infancy, nystagmus, and substantially reduced or non-recordable electroretinogram (ERG) responses (Kumaran et al., 2017). Aside from these core characteristics, variable disease phenotypes can exist among LCA patients, due to disease-causing variants occurring in at least 25 genes (den Hollander et al., 2008; Thompson et al., 2018). While most of these variants are inherited in an autosomal recessive fashion, LCA-causing mutations in the *CRX* gene are typically autosomal dominant. The CRX (cone-rod homeobox) protein is a transcription factor expressed early in newly differentiated photoreceptor cells during retinal development. In addition to autoregulation, CRX plays a critical role in driving expression of genes required for photoreceptor maturation and function, as well as cytoskeletal matrix of the active zone (CAZ) genes (Corbo et al., 2010; Hennig et al., 2008).

*CRX*-associated LCA (LCA7) accounts for roughly 2% of all LCA cases (Hull et al., 2014; Stone, 2007) and represents an especially devastating form of the disease, likely due to incomplete formation of photoreceptor outer segments, as has been reported in an LCA7 mouse model (Tran et al., 2014). Despite the presence of immature photoreceptor cells, preservation of the outer nuclear layer (ONL) is visible for some time in mice and in patients with LCA7 (Tran et al., 2014, Figure S1). While we know that disease-causing mutations in *CRX* have a significant impact on photoreceptor maturation and function, no treatments currently exist for LCA7. Therefore, novel therapeutic approaches must be developed to treat this dominant form of the disease.

Advancements in the human three-dimensional (3D) retinal organoid field have provided a promising alternative to rodent models for studying genetic retinal diseases (reviewed in Artero Castro et al., 2019; Kruczek and Swaroop, 2020). Therefore, we aimed to establish an *in vitro* human retinal organoid model system to study mutation-specific LCA7 phenotypes, allowing us to visualize the initiation and progression of disease phenotypes as the retina develops. Here, we reprogrammed peripheral blood mononuclear cells (PBMCs) derived from patients with a clinical diagnosis of LCA7. Each of the two resulting human induced pluripotent stem cell (hiPSC) lines harbors a dominant disease-causing mutation in *CRX*: *CRX*^*T155ins4/+*^ (c.464_465insGGCA; p.T155ins4) or *CRX*^*K88Q/+*^ (c.262A>C; p.K88Q). The hiPSC lines were then differentiated to generate 3D retinal organoids to establish a human-tissue-based model of LCA7. Characterization of these organoids revealed severe photoreceptor phenotypes at the cellular and molecular levels, along with a clear defect in photoreceptor outer-segment maturation. Since CRX is believed to be largely haplosufficient (Furukawa et al., 1999; Ibrahim et al., 2018), we performed a gene-editing-based proof-of-concept study to examine our ability to rescue these phenotypes by knocking out the mutant allele in our model. We found a robust increase in key photoreceptor markers in edited (*CRX*^*+/−*^) compared with unedited (*CRX*^*K88Q/+*^) organoids. This work not only establishes a model system to further study LCA7 disease mechanisms, it also provides evidence to support the development of an allele-specific gene-editing-based therapeutic approach to treat LCA7.

## Results

### LCA7 retinal organoids lack outer-segment-like projections

**Figure 1:**
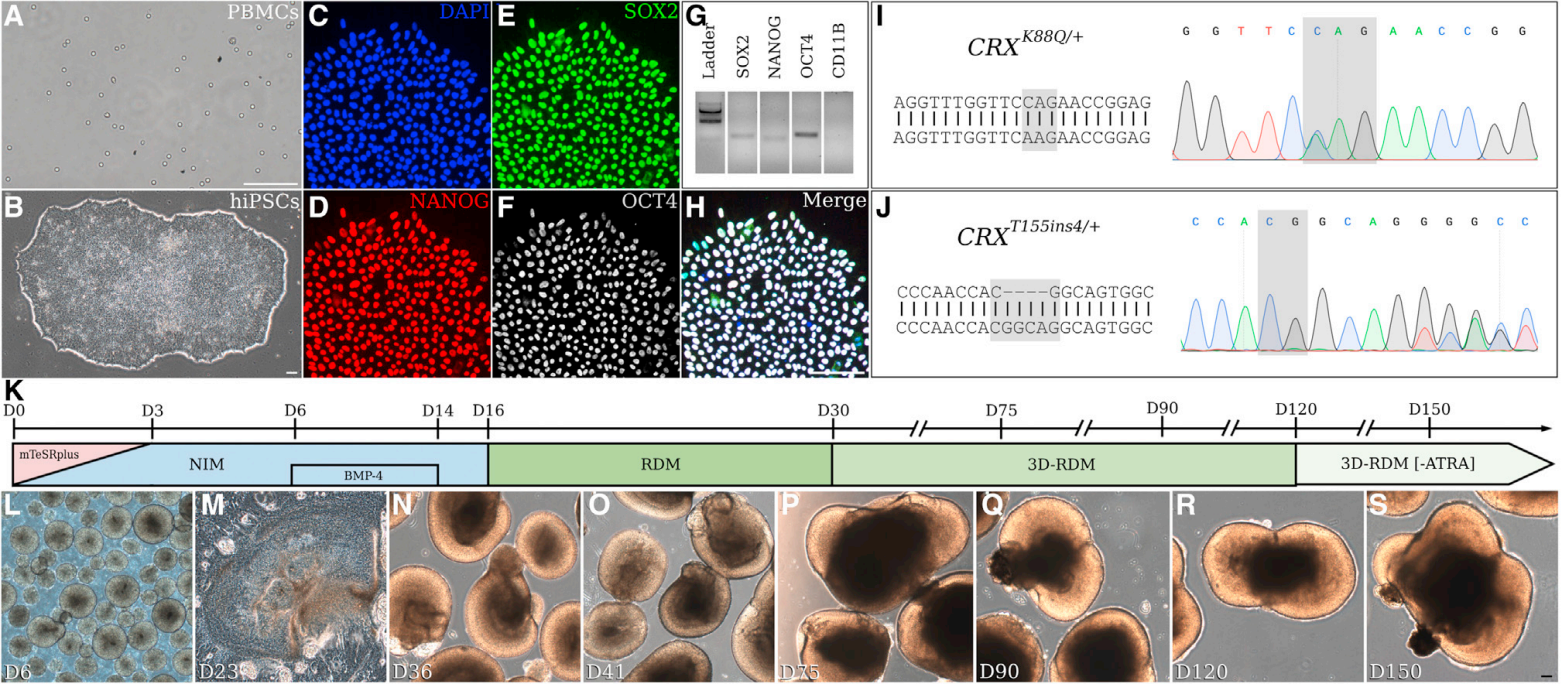
Characterization and differentiation of patient iPSC lines (A–S) Patient PBMCs (A) were reprogrammed to generate stable hiPSC lines (B). Immunocytochemistry was performed with antibodies against NANOG (D), SOX2 (E) and OCT3/4 (F), and cells were counterstained with DAPI (C). The merged image is shown in (H). Primers for *SOX2*, *NANOG*, *OCT4*, and *CD11B* were utilized for RT-PCR analysis (G). Sanger sequencing results for hiPSCs to confirm the presence of the *CRX*^*K88Q/+*^ (I) and *CRX*^*T155ins4/+*^ (J) variants. The retinal differentiation protocol timeline is summarized in (K), and representative images of the differentiation process are shown for the control hiPSC line (*CRX*^*WT*^) in (L)–(S). Scale bar (A, B, H, and S), 100 μm.

**Figure 2:**
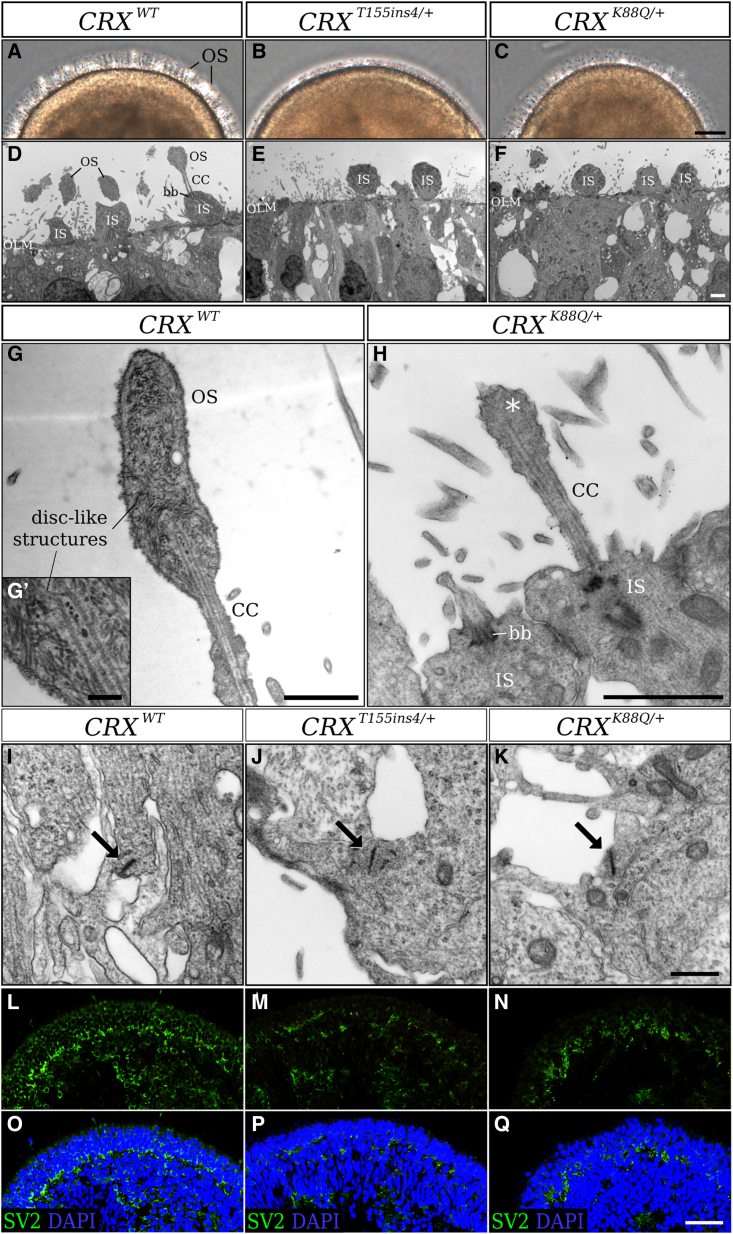
Altered outer-segment morphology and synapse markers in patient hiPSC-derived LCA7 retinal organoids compared with control organoids at D180 (A–C) Phase-contrast images for *CRX*^*WT*^ (A), *CRX*^*T155ins4/+*^ (B), and *CRX*^*K88Q/+*^ (C) retinal organoids at D180 of differentiation were taken along the edge of the organoids to show early outer-segment morphology. (D–K) TEM images for *CRX*^*WT*^ (D), *CRX*^*T155ins4/+*^ (E), and *CRX*^*K88Q/+*^ (F) retinal organoids were collected at D180 (n = 1 organoid per line). Early outer segments for the *CRX*^*WT*^ retinal organoids show disc-like structures at D180 (G, G′), whereas *CRX*^*K88Q/+*^ retinal organoids have underdeveloped outer-segment-like features (asterisk, H). TEM images show the formation of ribbon synapses (arrows) for *CRX*^*WT*^ (I), *CRX*^*T155ins4/+*^ (J), and *CRX*^*K88Q/+*^ (K) at D180. (L–Q) Immunolabeling for SV2 (green) is shown for *CRX*^*WT*^ (L and O), *CRX*^*T155ins4/+*^ (M and P), and *CRX*^*K88Q/+*^ (N and Q) at D180. DAPI counterstaining is shown in blue (O–Q). Scale bar (C), 10 μm; scale bars (D–H), 5 μm; scale bar (G′), 1 μm; scale bar (K), 500 nm; scale bar (Q), 100 μm. OS, outer segment; IS, inner segment; OLM, outer limiting membrane; CC, connecting cilium; bb, basal body. See also Figures S2 and S5.

Using previously published 3D retinal organoid differentiation protocols, we generated organoids from control (*CRX*^*WT*^) and LCA7 (*CRX*^*T155ins4/+*^ or *CRX*^*K88Q/+*^) hiPSC lines (Figure 1). The control (Figures 1L–1S) and LCA7 retinal organoids consistently showed comparable retinal morphology throughout differentiation up to day 150 (D150). By D180, LCA7 retinal organoids show obvious differences in the appearance of the projections that protrude from the edge of the organoids (Figures 2A–2C and S2). After further analysis using transmission electron microscopy (TEM), these projections appear to be early outer-segment-like structures in the control retinal organoids, with disc-like structures observed throughout (Figures 2D, 2G, and 2G′). For both *CRX* mutations, the retinal organoids have clear inner segments and some connecting cilia, but no outer segments were present along the outer edge of the organoids (Figures 2E, 2F, and 2H). The closest to an early outer segment we could find in any of the LCA7 organoids is shown in Figure 2H, and may simply represent the end structure of the connecting cilium, as no discs are visible. When these organoids were cultured through D240 of differentiation, the morphological defect remained visible in both of the LCA7 retinal organoids (Figure S4).

### Photoreceptor maturation is disrupted in LCA7 retinal organoids

**Figure 3:**
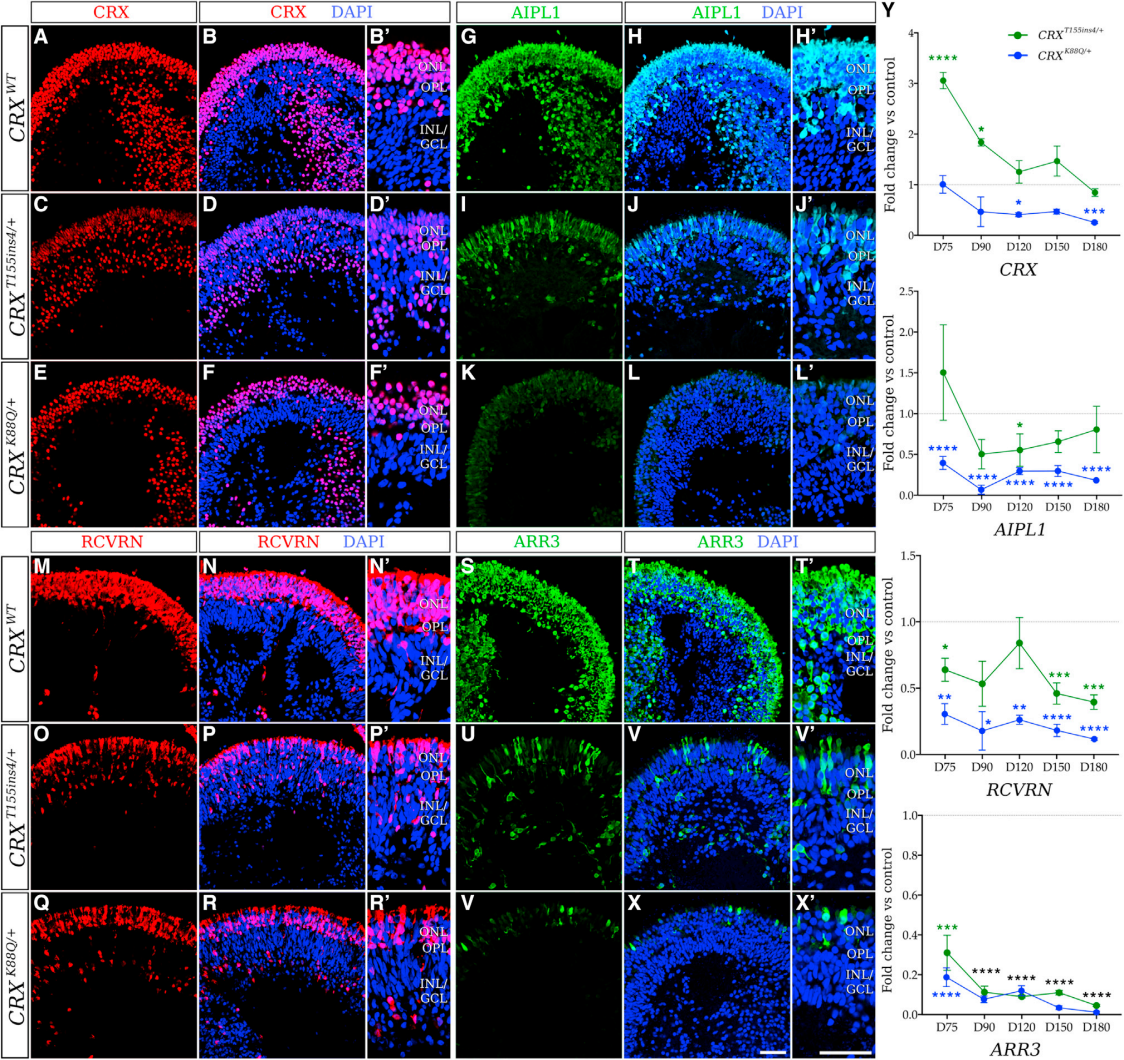
Altered expression of early photoreceptor cell markers in patient hiPSC-derived LCA7 retinal organoids compared with control organoids at D180 (A–Y) qRT-PCR data (I) are shown for *CRX*^*T155ins4/+*^ (green line) and *CRX*^*K88Q/+*^ (blue line) at D75, D90, D120, D150, and D180 as fold change compared with control organoids for *CRX*, *AIPL1*, *RCVRN*, and *ARR3* (n = 14 total organoids from two experimental replicates per line, per time point). The dotted line represents no change compared with control (y = 1). Immunofluorescence staining using antibodies against CRX (red; J–O′), AIPL1 (green; P–U′), RCVRN (red; V–AA′), and ARR3 (green; AB–AG′) are shown for control (*CRX*^*WT*^; J–K′, P–Q′, V–W′, AB–AC′), *CRX*^*T155ins4/+*^ (L–M′, R–S′, X–Y′, AD–AE′), and *CRX*^*K88Q/+*^ (N–O′, T–U′, Z–AA′, AF–AG′) retinal organoids at D180 (n = 9 total organoids from three experimental replicates per line). Nuclei are counterstained with DAPI (blue). Scale bar (AG and AG′), 100 μm. OPL, outer plexiform layer; INL/GCL, inner nuclear layer/ganglion cell layer. ^∗^p < 0.05, ^∗∗^p < 0.01, ^∗∗∗^p < 0.005, ^∗∗∗∗^p < 0.001. All statistical analyses were performed using one-way ANOVA with a Dunnett test to correct for multiple comparisons in GraphPad Prism 8 software. See also Figures S3, S4, and S7.

**Figure 4:**
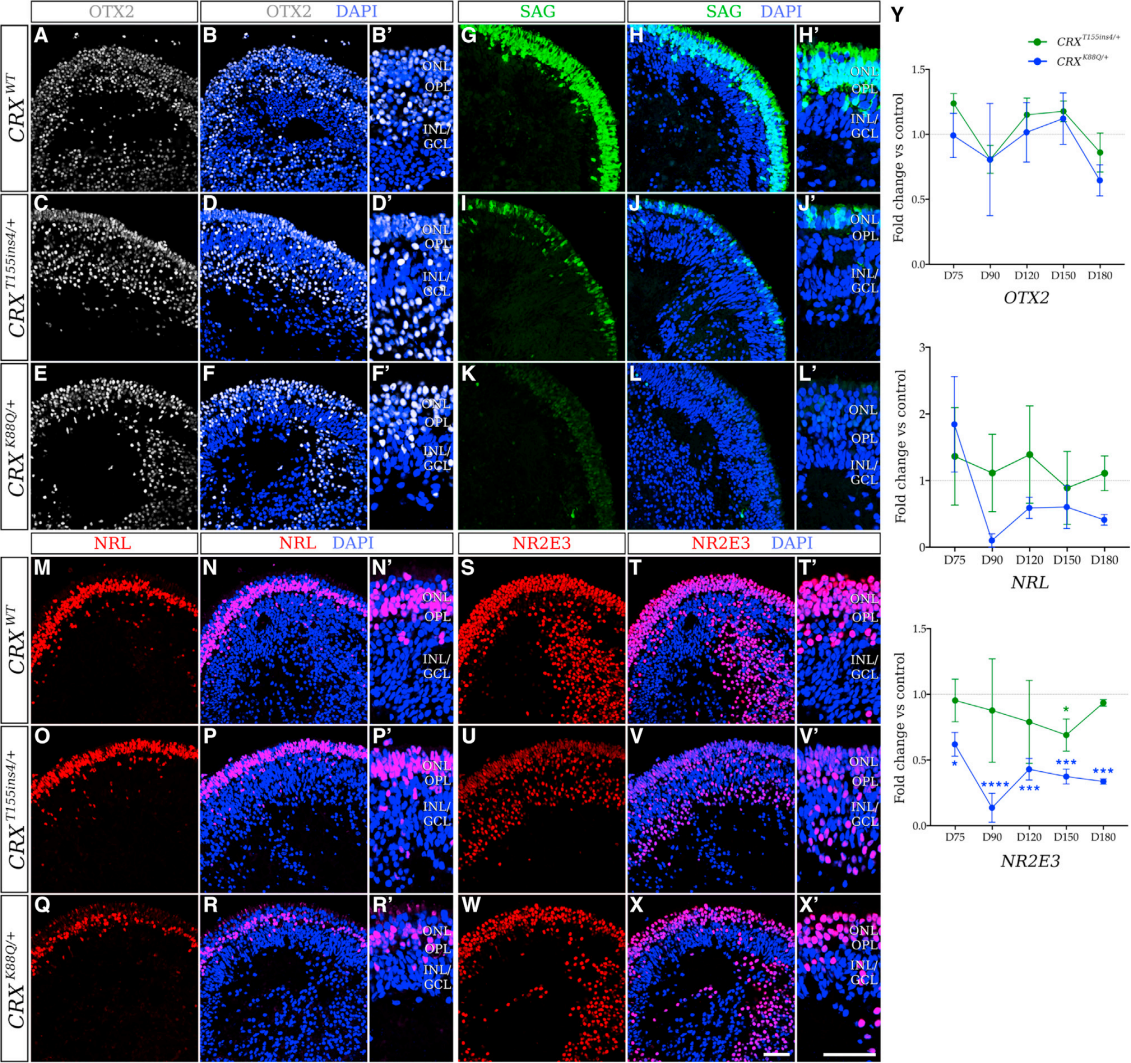
Altered expression of rod photoreceptor cell markers in LCA7 retinal organoids at D180 (A–Y) Immunofluorescence staining using antibodies against OTX2 (white; A–F′), SAG (green; G–L′), NRL (red; M–R′), and NR2E3 (red; S–X′) are shown for control (*CRX*^*WT*^; A–B′, G–H′, M–N′, S–T′), *CRX*^*T155ins4/+*^ (C–D′, I–J′, O–P′, U–V′), and *CRX*^*K88Q/+*^ (E–F′, K–L′, Q–R′, W–X′) retinal organoids at D180 (n = 9 total organoids from three experimental replicates per line). Nuclei are counterstained with DAPI (blue). OPL, outer plexiform layer; INL/GCL, inner nuclear layer/ganglion cell layer. qRT-PCR data (Y) are shown for *CRX*^*T155ins4/+*^ (green line) and *CRX*^*K88Q/+*^ (blue line) at D75, D90, D120, D150, and D180 as fold change compared with control organoids for *OTX2*, *NRL*, and *NR2E3* (n = 14 total organoids from two experimental replicates per line). The dotted line represents no change compared with control (y = 1). All statistical analyses were performed using one-way ANOVA with a Dunnett test to correct for multiple comparisons in GraphPad Prism 8 software. Scale bar (X and X′), 100 μm. ^∗^p < 0.05, ^∗∗∗^p < 0.005, ^∗∗∗∗^p < 0.001. See also Figures S3, S4, and S7.

**Figure 5:**
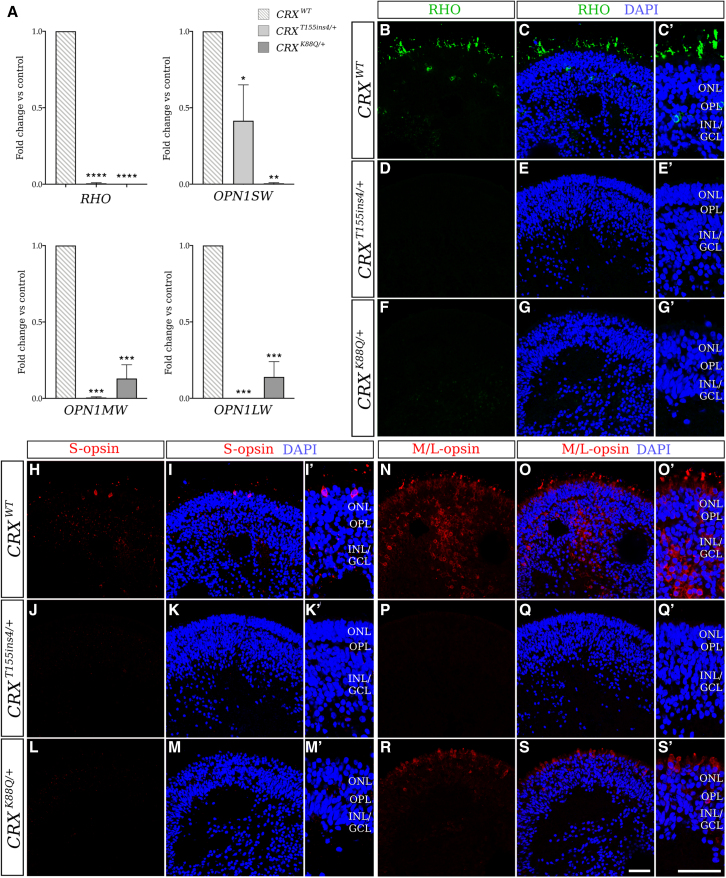
qRT-PCR and immunofluorescence staining reveal significant downregulation of late photoreceptor cell markers in LCA7 organoids at D180 (A–S′) qRT-PCR data (A) are shown for *CRX*^*WT*^ (gray lines), *CRX*^*T155ins4/+*^ (light gray), and *CRX*^*K88Q/+*^ (dark gray) at D180 as fold change compared with control organoids for *RHO*, *OPN1SW*, *OPN1MW*, and *OPN1LW* (n = 14 total organoids from two experimental replicates per line). ^∗^p < 0.05, ^∗∗^p < 0.01, ^∗∗∗^p < 0.005, ^∗∗∗∗^p < 0.001. Immunofluorescence staining using antibodies against RHO (green; B–G′), S-opsin (red; H–M′), and M/L-opsin (green; N–S′) is shown for control (*CRX*^*WT*^; B–C′, H–I′, N–O′), *CRX*^*T155ins4/+*^ (D–E′, J–K′, P–Q′), and *CRX*^*K88Q/+*^ (F–G′, L–M′, R–S′) retinal organoids at D180 (n = 9 total organoids from three experimental replicates per line). Nuclei are counterstained with DAPI (blue). Scale bar (S and S′), 100 μm. OPL, outer plexiform layer; INL/GCL, inner nuclear layer/ganglion cell layer. All statistical analyses were performed using one-way ANOVA with a Dunnett test to correct for multiple comparisons in GraphPad Prism 8 software. See also Figures S3 and S4.

To further characterize the LCA7 organoids at the cellular and molecular levels, immunofluorescence (IF) and quantitative real-time PCR (qRT-PCR) analyses were performed at multiple time points during differentiation: D75, D90, D120, D150, and D180. These data revealed comparable levels of OTX2 and a decrease in immunolabeling of recoverin (RCVRN) and AIPL1 for both *CRX*^*T155ins4/+*^ and *CRX*^*K88Q/+*^ organoids (Figures 3, 4, and S3). qRT-PCR data for each of these markers was consistent with the IF findings (Figure 3Y). Total *CRX* mRNA and immunolabeling data show an increase in total CRX for the *CRX*^*T155ins4/+*^ organoids, especially early in photoreceptor differentiation, and a decrease for the *CRX*^*K88Q/+*^ organoids compared with control (Figures 3A–3F’ and S3). While we only saw a mild decrease in neural retina-specific leucine zipper protein (NRL) levels, we observed a robust decrease in rod arrestin (SAG), and a complete absence of rhodopsin (RHO) in both LCA7 organoids. NR2E3 appeared normal in *CRX*^*T155ins4/+*^ organoids but low in *CRX*^*K88Q/+*^ organoids compared with control (Figures 4 and 5A–5G′). Immunolabeling for both LCA7 organoids showed low levels of cone arrestin (ARR3), blue cone opsin (S-opsin/OPN1SW), and green and red cone opsin (M/L-opsin/OPN1MW and OPN1LW), which is supported by qRT-PCR (Figures 3 and 5). Downregulation of key photoreceptor cell markers was more prominent in the *CRX*^*K88Q/+*^ organoids than the *CRX*^*T155ins4/+*^ organoids for all markers except M/L-opsin, which was higher in *CRX*^*K88Q/+*^ organoids at D180 (Figure 5).

In addition to the morphological defects, the histological phenotypes persisted out to D240 of differentiation (Figure S4). Other retinal cell types appeared unaffected by the mutations in *CRX* (Figure S5). Although clear photoreceptor defects can be observed in the LCA7 organoids, these cells form normal-looking ribbon synapses with unaltered expression of key ribbon synapse markers (Figures 2I–2K; Table S5). However, we did observe moderate changes in some synaptic vesicle markers, including SV2B, SNAP25, and STX3 (Figure 2L–2Q and S5D). We also saw a significant decrease in cytoskeletal matrix of the active zone (CAZ) marker levels, including *RIMS2*, *MPP4*, and *UNC119*, in both LCA7 organoids (Figure S5D; Table S5). These data are supported by previously published work demonstrating the role of CRX in presynaptic active zone formation in mice (Assawachananont et al., 2018).

### Single-cell transcriptome analysis reveals photoreceptor-specific defects in LCA7 retinal organoids

**Figure 6:**
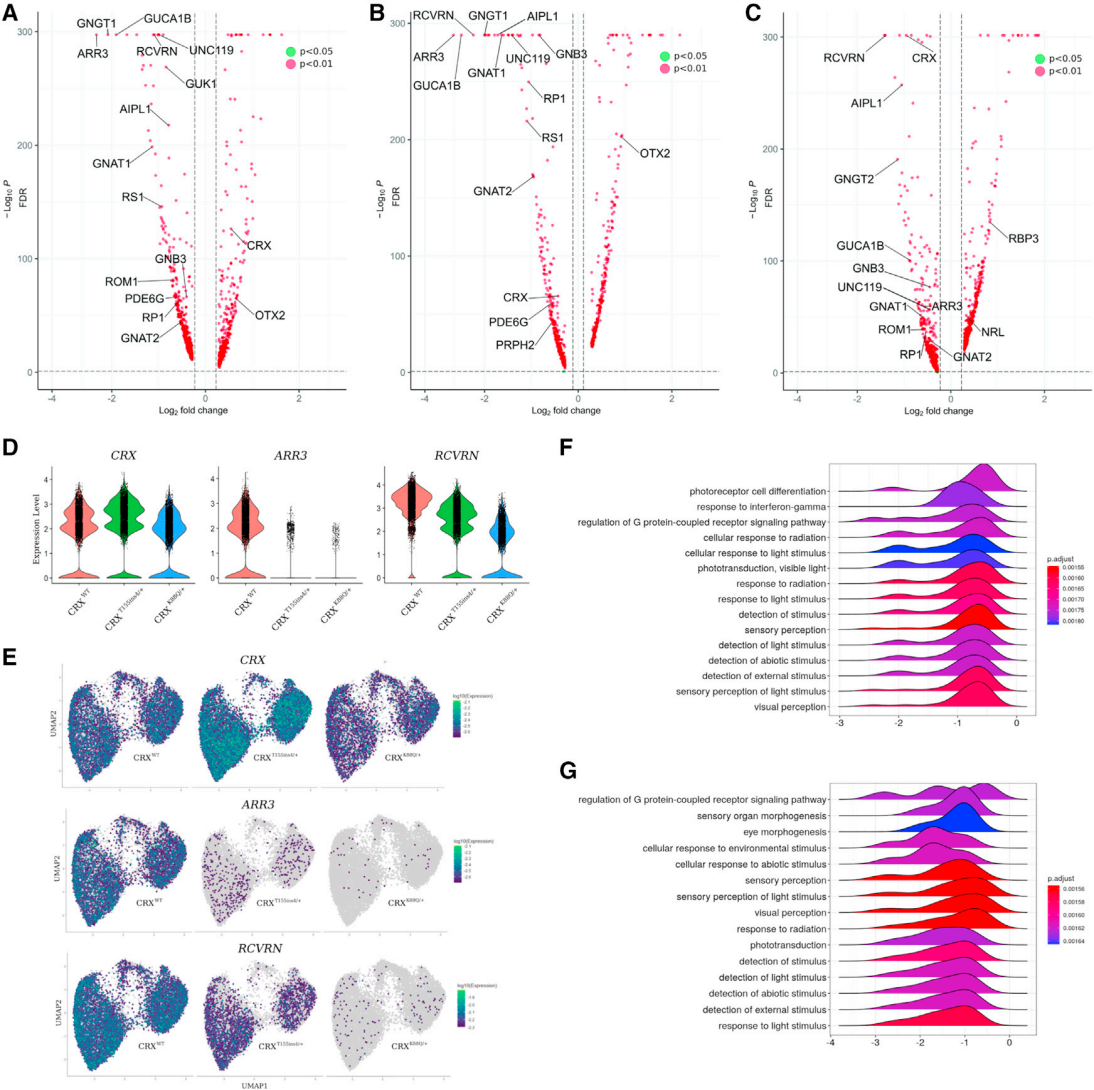
scRNA-seq of D150 reveals changes to photoreceptor transcripts in LCA7 versus control organoids (A–G) Volcano plots comparing *CRX*^*T155ins4/+*^ transcripts with those of control organoids (A), *CRX*^*K88Q/+*^ transcripts to those of control (B), and *CRX*^*K88Q/+*^ to *CRX*^*T155ins4/+*^ transcripts (C). Green dots represent genes with a log_2_ fold change >0.2 and an adjusted p value of p < 0.05. Red dots represent genes with a log_2_ fold change >0.2 and an adjusted p value of <0.01. Expression levels for *CRX*, *ARR3*, and *RCVRN* are shown as violin plots (D) for control (*CRX*^*WT*^, red), *CRX*^*T155ins4/+*^ (green), and *CRX*^*K88Q/+*^ (blue). UMAP graphs for *CRX*, *ARR3*, and *RCVRN* transcripts are also shown for all three genotypes (E). GSEA was performed for genes with significantly altered expression in *CRX*^*T155ins4/+*^ (F) and *CRX*^*K88Q/+*^ (G) organoids, and data for the top 15 categories are shown via ridge plots. The x axis represents fold change, and the y axis represents the number of genes. Libraries were prepared using n = 5 organoids per line. Statistical analyses were performed using the Wilcoxon rank-sum test and genes with log_2_ fold change of ≥0.25 and a p value <0.05 were considered significant. See also Figure S5.

To comprehensively capture changes in retinal gene expression for the LCA7 organoids compared with control, we performed single-cell RNA sequencing (scRNA-seq) using the 10X Genomics platform at D150 of differentiation. Three-dimensional principal component analysis to assess global gene expression changes revealed overlapping clusters for the two LCA7 lines, which were equally separated from the control line when plotting the first two principal components (Figure S5A), suggesting similar divergence in gene expression patterns for the LCA7 lines. The overlapping LCA7 clusters separated into distinct clusters in the third principal component, indicating that differences in gene expression exist between the two lines (Figure S5B). To broadly compare effects of the gene mutations on retinal and photoreceptor development and maturation, we carried out gene set enrichment analysis (GSEA) on significantly over- and under-expressed genes in the datasets for each mutation (Table S6). The top GSEA categories point primarily to a defect in photoreceptor maturation, leading to impaired phototransduction and light perception (Figure 6). In alignment with our qRT-PCR data, the scRNA-seq data revealed significant dysregulation of specific photoreceptor genes between control and LCA7 organoids, as well as mutation-specific differences in various genes, including *CRX*, *RCVRN*, *ARR3*, and *AIPL1* (Figures 6 and S5, Table S5). These data also revealed upregulation of OTX2 in our LCA7 organoids at D150 (Figure S5), which was not observed through IF or qRT-PCR data analyses. This change in *OTX2* expression might indicate a compensatory response by the diseased photoreceptor cells, as OTX2 and CRX share a number of gene targets in photoreceptor cells (Samuel et al., 2014).

### Allele-specific editing of mutant *CRX* allele in patient-derived hiPSCs rescues photoreceptor defects in LCA7 retinal organoids

**Figure 7:**
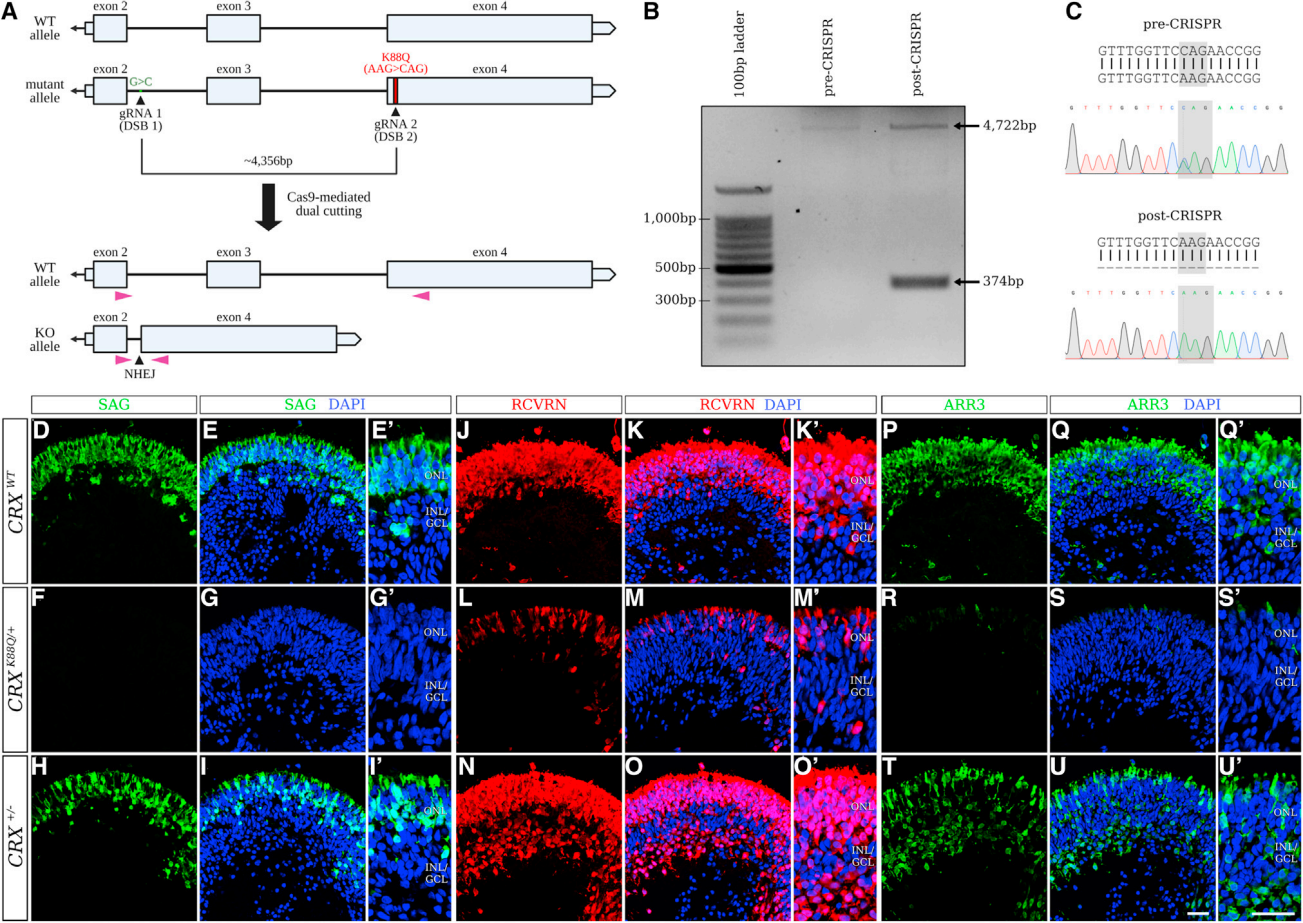
CRISPR/Cas9-mediated knockout of the mutant *CRX* allele in patient hiPSC (A–C) The CRISPR/Cas9 dual-cutting target sites are mapped onto the mutant allele of the *CRX* gene (A). PCR was performed using primers shown in (A; purple triangles), revealing an additional 374-bp band representing the edited “knockout (KO) allele” after CRISPR/Cas9 editing (B). Sanger sequencing was also utilized to confirm loss of the K88Q mutation after CRISPR-mediated editing (C). (D–U′) Immunofluorescence staining using antibodies against SAG (green; D–I′), RCVRN (red; J–O′), and ARR3 (green; P–U′) are shown for control (*CRX*^*WT*^; D–E′, J–K′, and P–Q′), *CRX*^*K88Q/+*^ (F–G′, L–M′, and R–S′), and *CRX*^*+/−*^ (H–I′, N–O′, and T–U′) retinal organoids at D180 (n = 3 organoids per line). Nuclei are counterstained with DAPI (blue). Scale bars (U and U′), 100 μm. OPL, outer plexiform layer; INL/GCL, inner nuclear layer/ganglion cell layer. See also Figures S6 and S7.

Based on data published in *Crx*^*+/−*^ mice (Furukawa et al., 1999), and a case study examining a human cohort with a *CRX* deletion mutation (Ibrahim et al., 2018), we know that one copy of CRX is sufficient to allow for proper maturation (although delayed) and function in photoreceptor cells. Therefore, we wanted to explore a therapeutic approach that would eliminate the mutant allele and allow the wild-type protein to be solely expressed. To this end, we took a dual-cutting approach using CRISPR/Cas9 tools. To achieve dual cutting, we first identified single nucleotide polymorphisms (SNPs) that were only present on the mutant allele. Using Sanger sequencing, we identified five SNPs on the mutant allele upstream of the *K88Q* mutation (data not shown), designed single guide RNA oligonucleotides (gRNAs) to target each SNP independently, and then cloned those guides into the PX459 plasmid containing wild-type Cas9 (Figure S7A). To eliminate the mutant *CRX* allele in *CRX*^*K88Q/+*^ hiPSCs, we simultaneously introduced two of the PX459 plasmids, one containing a gRNA targeting the *K88Q* mutation, and one containing a gRNA targeting an upstream allelic SNP (Figure 7A). The most successful gRNA targeted an SNP located just downstream of exon 2, within the intron (Table S2). The edited allele could be identified as a 374-bp band in a PCR gel (Figure 7B), and Sanger sequencing confirmed the loss of the *K88Q* mutation (Figure 7C). The resulting hiPSC line will be referred to as *CRX*^*+/−*^ going forward.

The *CRX*^*+/−*^ hiPSCs were differentiated alongside control and *CRX*^*K88Q/+*^ hiPSCs, and organoid morphology for the *CRX*^*+/−*^ line appeared normal through D180 of differentiation. IF data at D90, D120, D150, and D180 showed a dramatic increase in SAG, RCVRN, and ARR3 levels in the *CRX*^*+/−*^ organoids compared with *CRX*^*K88Q/+*^ (Figures 7D–7U′ and S6). While we found RCVRN levels to be comparable between *CRX*^*+/−*^ and control organoids at D180, the levels of SAG and ARR3 in the *CRX*^*+/−*^ organoids do not fully reach those of the control organoids. This could suggest a mild delay in photoreceptor maturation, which would be in line with the developmental delay observed in *CRX*^*+/−*^ mouse retinae (Furukawa et al., 1999).

## Discussion

In the current study, we set out to generate an *in vitro* model of LCA7, using 3D human retinal organoids. iPSC lines were made from LCA patients with dominant disease-causing variants in the *CRX* gene. Those hiPSC lines were then differentiated to generate retinal organoids with LCA7 mutations: *CRX*^*T155ins4/+*^ or *CRX*^*K88Q/+*^. We discovered a clear morphological phenotype in our LCA7 organoids by D180, at which time the organoids had not developed outer-segment-like projections, in contrast to control organoids. This phenotype persisted to D240, which was the latest time point we analyzed for this study. We also observed a photoreceptor cell-specific phenotype within the LCA7 organoids, with decreased expression of early pan-photoreceptor markers, including recoverin/RCVRN and AIPL1. Both LCA7 organoids exhibited lower levels of rod-specific markers, including rod arrestin/SAG and rhodopsin/RHO, and cone-specific markers, including cone arrestin/ARR3 and all three of the cone opsins. All of the markers analyzed in this study are critical for normal photoreceptor function, and downregulation of any one of them could lead to dysfunctional photoreceptor cells. Therefore, dysregulation of all of these key genes at the same time in the LCA7 organoids indicates severe photoreceptor disease. Together, these data suggest the presence of immature photoreceptor cells in the LCA7 retinal organoids, which aligns nicely with previously published work in LCA7 mice (Tran et al., 2014), and retinal organoids (Kruczek et al., 2021), providing a representative model system for this disease.

Furthermore, we saw distinct differences in the photoreceptor phenotype for the *CRX*^*K88Q/+*^ organoids compared with the *CRX*^*T155ins4/+*^ organoids, with the *K88Q* mutation resulting in a more significant decrease in mRNA and/or protein for some of the affected markers: CRX, RCVRN, AIPL1, SAG, and ARR3. We also observed downregulation of two key photoreceptor markers, NRL and NR2E3, that were unaffected by the *T155ins4* mutation at D180. In contrast, M-opsin/OPN1MW and L-opsin/OPN1LW were downregulated to a larger extent in the *CRX*^*T155ins4/+*^ organoids compared with *CRX*^*K88Q/+*^. Finally, the morphology differs slightly between the *CRX*^*T155ins4/+*^ and *CRX*^*K88Q/+*^ organoids, with the former having more severely stunted inner segment projections. Despite that, neither of the LCA7 lines form outer segments. Interestingly, one of the *T155ins4* patients exhibited slower visual decline (hand-motion vision and a measurable Goldmann visual field peripherally at age 12 years) compared with the patient with the *K88Q* mutation (bare light perception by the age of 4 years), aligning nicely with the severity of the photoreceptor phenotype in our retinal organoids. These differences may be attributed to the type and location of the mutations, with *K88Q* affecting the homeobox domain, and the *T155ins4* mutation causing a frameshift and early stop, likely resulting in a truncated CRX^T155ins4^ protein that lacks a complete transactivation domain. This early stop results in the formation of a truncated CRX^T155ins4^ protein. The exact mechanism(s) for these variant-specific differences will need to be investigated further in future experiments, and could lend insight into the cause(s) for phenotypic variability observed in LCA7 patients. The mechanism(s) behind the variant-specific differences in *CRX* mRNA and protein levels is unknown. One hypothesis is that the variants alter the stability of the CRX mRNA molecules, with the T155ins4 mutation resulting in stabilized mRNA, and the K88Q mutation resulting in destabilized mRNA, similar to what has been hypothesized in mouse models (Tran et al., 2014). Another hypothesis is that there is a mutant protein-mediated reduction in overall CRX function, which subsequently drives the expression of both wild-type and T155ins4 *CRX* alleles in an attempt to compensate. The latter hypothesis, however, does not explain the observed decrease in the *CRX*^*K88Q/+*^ organoids. Investigations into this intriguing phenomenon will be carried out in future studies.

A recent study generated a similar model, where they made retinal organoids from patient-derived hiPSCs harboring dominant mutations in *CRX* (Kruczek et al., 2021). Although the mutations studied by Kruczek and colleagues differ slightly from those shown here (*CRX*^*I138fs48/+*^ and *CRX*^*K88N/+*^), the resulting proteins likely act quite similarly to ours. In alignment with that hypothesis, our data for the *CRX*^*T155ins4/+*^ organoids show similar trends to those of the *CRX*^*I138fs48/+*^ organoids at corresponding time points for CRX, recoverin, rhodopsin, and L/M-opsin levels. Likewise, our *CRX*^*K88Q/+*^ organoids have expression patterns much like those of the *CRX*^*K88N/+*^ organoids. Despite these similarities, *CRX*^*K88Q/+*^ organoids appear to have a more drastic decrease in RCVRN immunolabeling at both D180 and D240 compared with 200-day-old *CRX*^*K88N/+*^ organoids, whereas there is less of a change in L/M-opsin immunolabeling in our organoids compared with theirs. Finally, in contrast to the *CRX*^*I138fs48/+*^ organoids, we observed a significant downregulation of *OPN1SW* mRNA in the *CRX*^*T155ins4/+*^ organoids compared with control.

Gene augmentation has been used successfully to treat other forms of LCA, like the RPE65-based voretigene neparvovec (Russell et al., 2017), and Kruczek and authors show promising evidence of rescue using gene augmentation to overexpress wild-type CRX in *CRX*-mutant retinal organoids using adeno-associated viruses (AAVs) (Kruczek et al., 2021). Here, we use an alternative approach that utilizes CRISPR/Cas9-based gene editing to selectively eliminate the mutant allele, allowing wild-type CRX to resume normal function unhindered. This approach was established because most LCA-causing mutations in *CRX* are dominant, and we know that CRX is largely haplosufficient (Furukawa et al., 1999; Ibrahim et al., 2018). The CRISPR/Cas9 system was used in this study due to its efficiency in generating targeted double-stranded DNA breaks at precise locations in the genome. For the initial proof-of-concept study presented here, the allele harboring the *K88Q* mutation in the *CRX*^*K88Q/+*^ hiPSC line was targeted, along with an upstream SNP present on the mutant allele of *CRX*. These cells were then differentiated and were found to have a substantial increase in CRX, RCVRN, ARR3, and SAG compared with the unedited *CRX*^*K88Q/+*^ organoids. These data suggest that the allele-specific editing approach is a promising strategy to provide at least moderate rescue of photoreceptor cell development and maturation in retinal organoids by D180. Going forward, we will test this strategy in developing organoids, for both the *CRX*^*K88Q/+*^ and *CRX*^*T155ins4/+*^ lines, to determine efficiency of targeting photoreceptor cells with these tools, and to establish a timeline for effective therapeutic intervention. This method could theoretically be applied to any dominant *CRX* mutation, and could work for additional dominant genetic diseases in other tissues, providing a broader application outside the specific mutations presented in this work.

## Experimental procedures

### PBMC collection and reprogramming

This study was approved by the Institutional Review Board (Induced Pluripotent Stem Cells for Retinal Research, IRB# 18-26,409) at the University of California San Francisco (UCSF). Patients were seen by a retinal specialist at one of the UCSF Department of Ophthalmology clinics, and were recruited into the study after informed consent. Relevant donor information can be found in Table S1, and the full sample processing and reprogramming details can be found in the supplemental experimental procedures.

### *CRX* gene sequencing

To determine the presence or loss of *CRX* variants in the hiPSC lines, a 597-bp region of the *CRX* gene surrounding the variants was amplified using PCR with a Taq polymerase (Thermo Scientific, EP0402). The reactions were run through a 1% agarose gel, and the DNA was isolated and cleaned up using the QIAquick Gel Extraction Kit (Qiagen, 28,704). *CRX* amplicons were then sent to Eurofins Scientific for Sanger sequencing. This method was also used to identify non-disease-causing allelic SNPs, which could then be used as a target for Cas9-mediated disruption of the mutant *CRX* allele. See supplemental experimental procedures for more details.

### Generating CRISPR-Cas9 plasmids and homology-directed repair donor constructs

All CRISPR-Cas9 single guide RNAs (gRNAs) used for this study were designed with the tools available through the Benchling informatics platform (www.benchling.com). gRNAs targeting the dominant disease-causing *CRX* variant *CRX*^*K88Q*^ (c.262A>C), as well as gRNAs targeting allele-specific SNPs between exons 2 and 4 of the *CRX* gene were generated. Single gRNAs were cloned into the pSpCas9(BB)-2A-Puro (PX459) plasmid (Addgene, 62988) containing the *Streptococcus pyogenes Cas9* (*spCas9*) gene and the puromycin resistance (*PuroR*) gene for puromycin-mediated clonal selection. Oligo sequences can be found in Table S2.

### Disruption of the mutant *CRX* allele in hiPSCs

The lipofectamine-based transfection protocol described above was also utilized to disrupt the mutant *CRX* allele in hiPSCs. To eliminate the mutant *CRX* allele, hiPSCs containing the *CRX*^*K88Q/+*^ genotype were simultaneous transfected with two separate PX459-Cas9 plasmids (at a 1:1 ratio): one containing a gRNA directly targeting the *K88Q* mutation (3 μg), and one containing a gRNA targeting an SNP near exon 2 on the mutant allele (3 μg). After puromycin selection, individual clones were assessed by PCR amplification followed by Sanger sequencing of the *CRX* gene.

### Retinal organoid differentiation

Retinal organoids were generated using a protocol derived from previously published work (Capowski et al., 2019; Ohlemacher et al., 2015; Zhong et al., 2014). Briefly, hiPSCs were grown on six-well tissue culture (TC)-treated plates coated with Matrigel (Corning, 354234), and fresh mTeSR Plus medium was given to the cells every other day. To passage cells, each well was rinsed with 1× Dulbecco's phosphate-buffered saline (DPBS) before applying EDTA (1:1,000 in 1× DPBS) to the cells for 2 min. EDTA was then aspirated and cells were given fresh mTeSR Plus medium before colonies were gently lifted using a cell lifter. Cells used to maintain the line were plated in a new Matrigel-coated six-well plate. Colonies used to make retinal organoids were carried through the differentiation protocol (Figure 1K). Phase images of the retinal organoids were captured using an Olympus IX51 inverted microscope. See supplemental experimental procedures for full details and media recipes.

### Immunocytochemistry and immunofluorescence

Each newly reprogrammed hiPSC clone was grown on a 24-well TC-treated cell culture plate to reach ∼50%–70% confluency. The cells were then fixed on the plate for 10 min at room temperature using 4% paraformaldehyde (PFA) prior to staining. Retinal organoids were collected at D75, D90, D120, D150, D180, and D240 (n = 3/clone per collection, with three separate collections per time point) for each genotype. Organoids were fixed for 30 min in 4% PFA. Cells and tissue sections were stained with various antibodies (see Table S3 for full list of antibodies) and counterstained with 4′,6-diamidino-2-phenylindole (DAPI, 1 μg/mL; Roche, 10236276001) for 2 min, before being imaged. Extended processing details are described in supplemental experimental procedures.

### qRT-PCR analysis

Total RNA was extracted from six or seven retinal organoids per collection (n = 2 collections per clone) using the RNeasy Extraction Kit (Qiagen, 74104). cDNA was then generated using the iScript cDNA Synthesis Kit (Bio-Rad, 1708891). To run qRT-PCR experiments, the iTaq Universal SYBR Green Supermix (Bio-Rad, 1725124) was employed. Primer sequences can be found in Table S4. All data summary graphs were made using GraphPad Prism 8, and all statistical analyses were performed via one-way ANOVA with a Dunnett test to correct for multiple comparisons. See supplemental experimental procedures for more details.

### TEM

D180 retinal organoids (hiPSC *CRX*^*WT*^ control, *CRX*^*T155ins4/+*^, and *CRX*^*K88Q/+*^) were fixed overnight in 4% PFA (Fisher Acros, 41678-500) + 2.5% glutaraldehyde (Polysciences, 00376) in 0.1 M PO_4_ buffer solution and rinsed four or five times with 0.1 M PO_4_ buffer. Fixed organoids were then stained in 1% osmium tetroxide (OsO_4_; Electron Microscopy Sciences, 19180) in distilled H_2_O (dH_2_O) for 1 h at room temperature. Organoids were washed in five changes of cold dH_2_O, followed by staining in 2% uranyl acetate (Electron Microscopy Sciences, 22400) in dH_2_O for 1 h at 37°C. After washing in dH_2_O, the organoids were dehydrated in ethanol (50%, 70%, 95%, 2 × 100%), for 20 min each. The dehydration process was continued with five 20-min incubations in absolute ethanol. The organoids were exposed to propylene oxide (PO; Polysciences Inc., 00235-1) for two 5-min rinses, and then infiltrated with a 1:1 ratio of Epon resin (Epon-812; Electron Microscopy Sciences, 13940) and PO overnight at room temperature. The next day, the mixture was replaced with degassed 100% Epon resin for 1–2 h. The organoids were then embedded in 100% Epon resin in PELCO Silicone Rubber Molds (Ted Pella Inc., PELCO 105) via polymerization for 48 h at 60°C. Sections 70 nm thick were collected on copper mesh grids and imaged using a Philips Tecnai 10 electron microscope.

### scRNA-seq data analysis

Five retinal organoids per line (*CRX*^*WT*^, *CRX*^*T155ins4/+*^, and *CRX*^*K88Q/+*^) were dissociated on D150 using the Papain Dissociation System (Worthington Biochemical Corp, LK003150) following manufacturer's instructions. Briefly, after applying pre-warmed papain solution (20 U/mL papain +0.0005% DNase) for 20 min at 37°C with gentle agitation, organoids were triturated using a sterile transfer pipette to generate a single-cell suspension. The cell suspension was centrifuged at 300 x g for 5 min to pellet the cells. After removing the supernatant, cells were resuspended in fresh 1:1 DMEM/F12 + 10% FBS and counted to determine cell concentration. Cells were then spun down once more at 300 × *g* for 5 min to pellet the cells. Cells were resuspended in 1× DPBS with 0.04% BSA to a final concentration of 1,300 cells/μL in 100 μL per line. Cells were partitioned and barcoded using a 10X Chromium Controller with a target recovery of 8,000 cells per line, followed by library construction with the Chromium Next GEM Single Cell 3ʹ Dual Index Reagent Kits v3.1 (10X Genomics, PN-1000269) according to the manufacturer's instructions. Single-cell libraries were sequenced on an Illumina NovaSeq at the UCSF Institute for Human Genetics Core.

The mean number of reads per cell for each line was 13,148 (*CRX*^*WT*^), 8,525 (*CRX*^*T155ins4/+*^), and 14,337 (*CRX*^*K88Q/+*^), and the median unique molecular identifier (UMI) per cell was 926 (*CRX*^*WT*^), 642 (*CRX*^*T155ins4/+*^), and 868 (*CRX*^*K88Q/+*^). CellRanger software (version 5.0.0) was used with default parameters for library demultiplexing, fastq file generation, read alignment, and UMI quantification. Further processing was carried out in Rstudio using Seurat 4.0.0 (Stuart et al., 2019). Seurat objects were crated to include genes expressed in at least 20 cells and cells with a minimum of 500 genes per cell. Data were further filtered to exclude cells with >2,500 genes or >20% mitochondrial genes. The datasets were merged and exported to Monocle 3 (Cao et al., 2019). Data were log normalized, and principal components were determined and scaled. Data from control (*CRX*^*WT*^), *CRX*^*K88Q/+*^, and *CRX*^*T155ins4/+*^ organoids were aligned to remove batch effects using Batchelor in Monocle 3 (Haghverdi et al., 2018). To visualize nuclear transcriptomic profiles in two-dimensional space, Uniform Manifold Approximation and Projection (UMAP) was performed with the following parameters: umap.min_dist = 0.2, umap.n_neighbors = 10. Differential expression analysis was performed on normalized scaled data in Seurat using the Wilcoxon rank-sum test and genes with logfc.threshold of 0.25 and p value <0.05 were considered for volcano plots and enrichment analysis. Volcano plots were generated using the EnhancedVolcano package (www.bioconductor.org/packages/release/bioc/vignettes/EnhancedVolcano/inst/doc/EnhancedVolcano.html). GSEA was performed on differentially expressed genes using ClusterProfShinyGSEA from the NASQAR toolbox (Yousif et al., 2020).

### Data and code availability

The datasets generated during this study are available on GEO: GSE184080.

## Author contributions

K.R.C. planned experiments, conducted experiments, performed data analysis, and wrote the paper. S.C. conducted experiments and edited the paper. A.T.M. conducted experiments and edited the paper. J.L.D. conducted experiments, and wrote and edited the paper, D.A.L. planned experiments, performed data analysis, and wrote the paper.

## Conflicts of interest

K.R.C., S.C., and D.A.L. declare no competing interests. A.T.M. has been a consultant for Roche, Nightstar, and 4-D Therapeutics, and has received funding to participate in clinical trials of gene therapy from AGTC Therapeutics. J.L.D. is a scientific advisory board member for Sparing Vision Inc, California Institute for Regenerative Medicine, and Vedere Bio; a consultant for Astellas, Biogen/Nightstarx Therapeutics, DTx Pharma, Editas Inc, Eloxx, Eyevensys, Gyroscope Therapeutics, and ProQR Therapeutics Inc; and is on the data safety monitoring board for clinical trials with AGTC Therapeutics and Spark Therapeutics. J.L.D. receives funding to support clinical trials from 10.13039/100007819Allergan/10.13039/100006483Abbvie, Acucela, and 10.13039/100005614Biogen/Nightstarx Therapeutics.

## Supplemental information

Document S1. Supplemental experimental procedures, Figures S1–S7, and Tables S1–S6

Document S2. Article plus supplemental information
