# Supplementary material for: Allele-specific gene editing to rescue dominant CRX-associated LCA7 phenotypes in a retinal organoid model
Source: Stem Cell Reports. 2021 Oct 14;16(11):2690–702. doi: 10.1016/j.stemcr.2021.09.007 (PMC8580887; doi:10.1016/j.stemcr.2021.09.007)
Supplement: Document S1. Supplemental experimental procedures, Figures S1–S7, and Tables S1–S6 [file mmc1.pdf]

Stem Cell Reports, Volume *16*

Supplemental Information

Allele-specific gene editing to rescue dominant *CRX*-associated LCA7 phenotypes in a retinal organoid model

Kathleen R. Chirco, Shereen Chew, Anthony T. Moore, Jacque L. Duncan, and Deepak A. Lamba

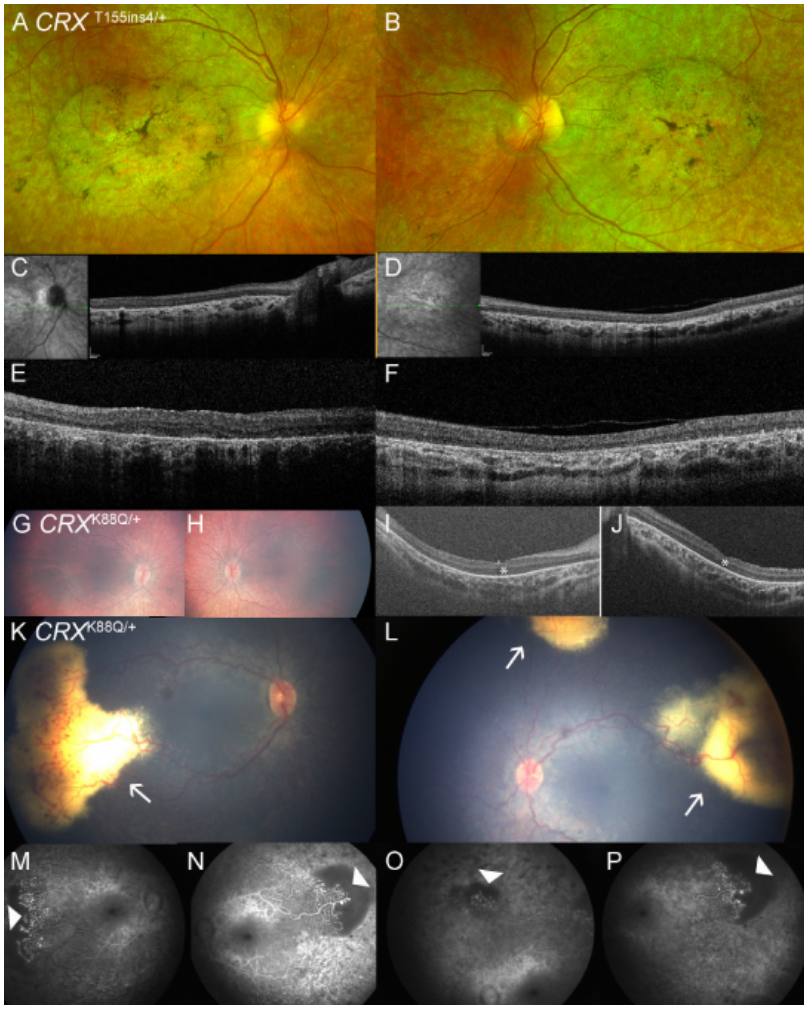

Figure S1. Retinal phenotype of two patients with CRX-associated LCA.
Color fundus photos of the right (**A**) and left (**B**) eyes of a 20-year-old with LCA ($CRX^{T155ins4/+}$) show bilateral macular atrophy with pigment clumping, mild disc pallor, retinal vascular attenuation, and diffuse retinal pigment epithelial (RPE) mottling outside the macular atrophy. Infrared fundus images and optical coherence tomography (OCT) scans through the macula of the right (**C**) and left (**D**) eyes reveal severe loss of the outer retinal layers in the macula magnified in panels (**E**) and (**F**), respectively; the posterior hyaloid is partially detached in the macula of the left eye (**D** and **F**). Color fundus photos of the right (**G**) and left (**H**) eyes of a 1 year old with LCA ($CRX^{K88Q/+}$) show diffuse RPE mottling and mild retinal vascular attenuation with a small region of preserved outer nuclear layer band centrally (*) visible in macular OCT scans from the right (**I**) and left (**J**) eyes. Color fundus photos at age 5 years show peripheral retinal vascular telangiectasias with lipid exudate temporally (arrows) in the right eye (**K**) and temporally and superiorly in the left eye (**L**). Early frames of fluorescein angiography at 1 minute and 32 seconds after injection of fluorescein dye in the right eye (**M**) and 25 seconds in the left eye (**N**) eyes show retinal vascular telangiectasias with peripheral nonperfusion (arrowheads) that stain in the mid-transit phase, 1 minute and 8 seconds (left eye, panel **O**) and 2 minutes and 37 seconds (left eye, panel **P**).

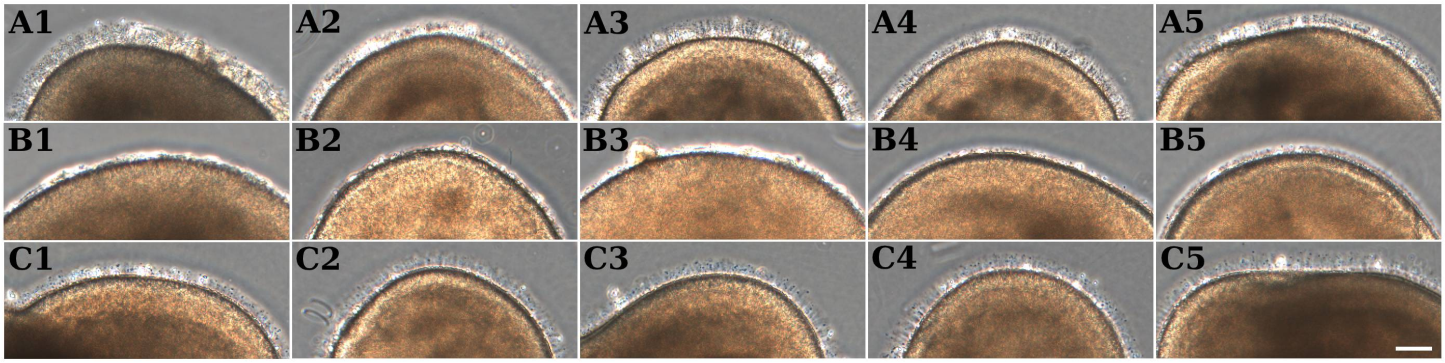

Figure S2. Consistency in early outer segment morphology across multiple differentiation experiments, *related to Figure 2*. Phase-contrast images for *CRX*^{WT} (**A1-A5**), *CRX*$^{T155ins4/+}$ (**B1-B5**), and *CRX*$^{K88Q/+}$ (**C1-C5**) retinal organoids at D180 were taken along the edge to show early outer segment morphological variability between five replicate differentiation experiments per line. Scale bar = 100μm.

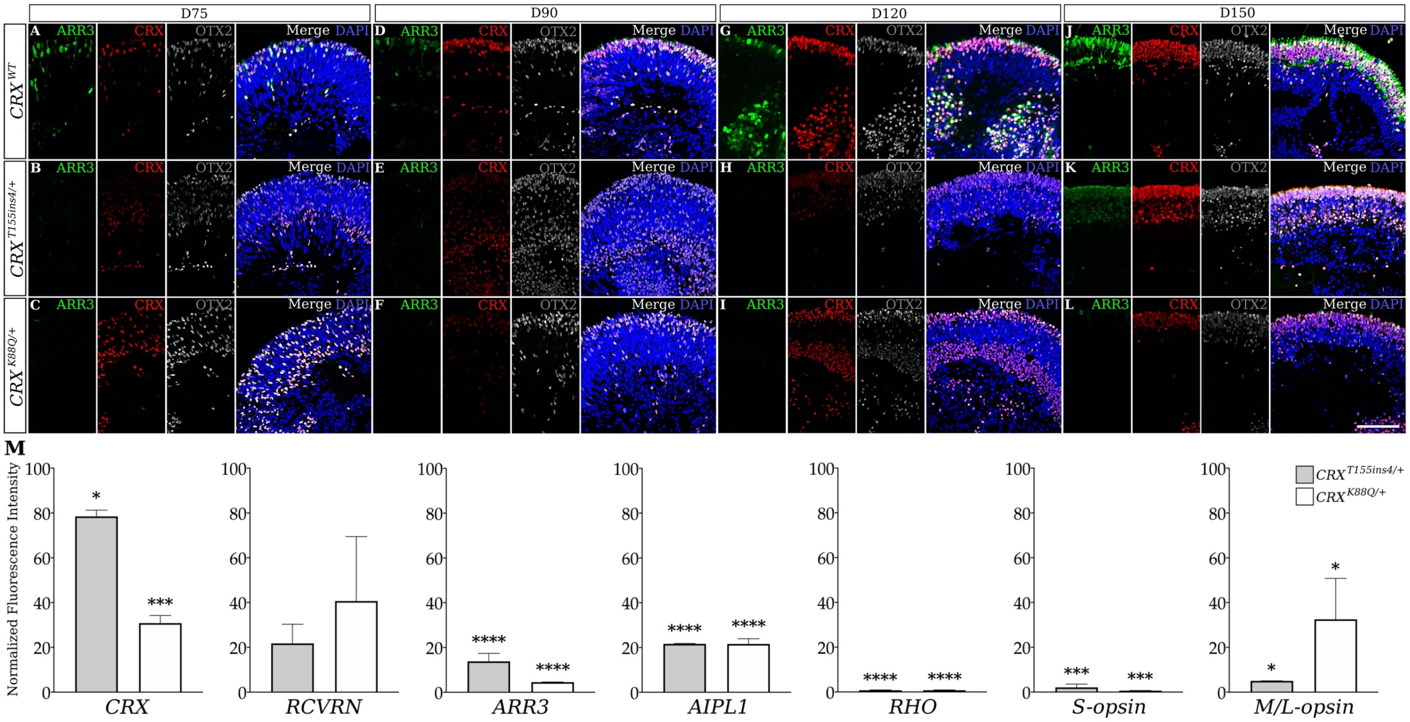

Figure S3. Immunofluorescence staining in retinal organoids at D75, D90, D120, and D150, and fluorescence pseudo-quantification in retinal organoids at D180, *related to Figure 3, Figure 4, and Figure 5*.

Antibodies against ARR3 (green), CRX (red), and OTX2 (white) are shown for CRX^{WT} (**A**, **D**, **G**, **J**), $CRX^{T155ins4/+}$ (**B**, **E**, **H**, **K**), and $CRX^{K88Q/+}$ (**C**, **F**, **I**, **L**) retinal organoids at D75 (**A-C**), D90 (**D-F**), D120 (**G-I**), and D150 (**J-L**). Nuclei are counterstained with DAPI (blue). Scale bar = 100µm. Fluorescence intensity (**M**) was quantified for CRX, RCVRN, ARR3, AIPL1, RHO, S-opsin, and M/L-opsin immunolabeling at D180 for $CRX^{T155ins4/+}$ (gray bar) and $CRX^{K88Q/+}$ (white bar) organoids. Fluorescence intensities were normalized to those of control organoids (n=3 organoids per marker per line).

* = $p<0.05$, *** = $p<0.005$, **** = $p<0.001$. All statistical analyses were performed using one-way ANOVA with a Dunnett test to correct for multiple comparisons in GraphPad Prism 8 software.

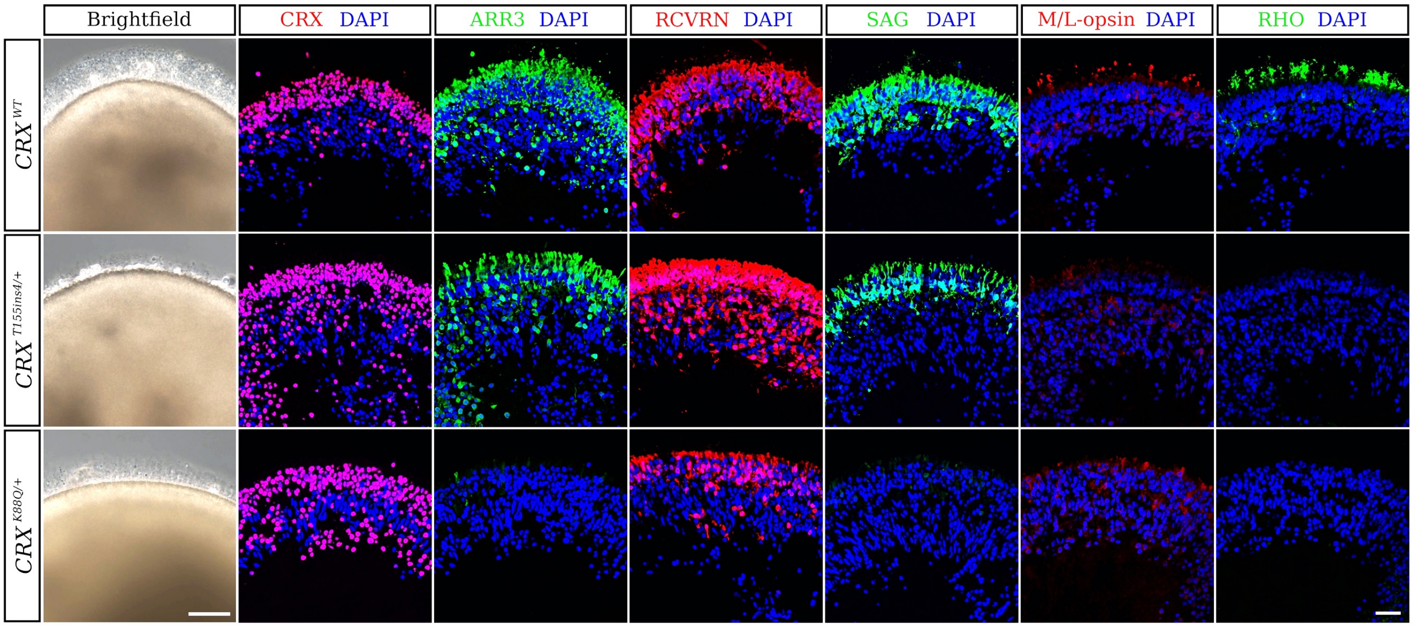

Figure S4. LCA7 retinal organoids show morphological defects and changes to photoreceptor cell markers at D240, *related to Figure 3, Figure 4, and Figure 5.*
Outer segment morphology is also shown in phase-contrast images of D240 retinal organoids for CRX^{WT}, $CRX^{T155ins4/+}$, and $CRX^{K88Q/+}$. IF staining using antibodies against CRX (red), ARR3 (green), RCVRN (red), SAG (green), M/L-opsin (red), RHO (green) are shown for all three genotypes. Nuclei are counterstained with DAPI (blue). Scale bars = 100μm.

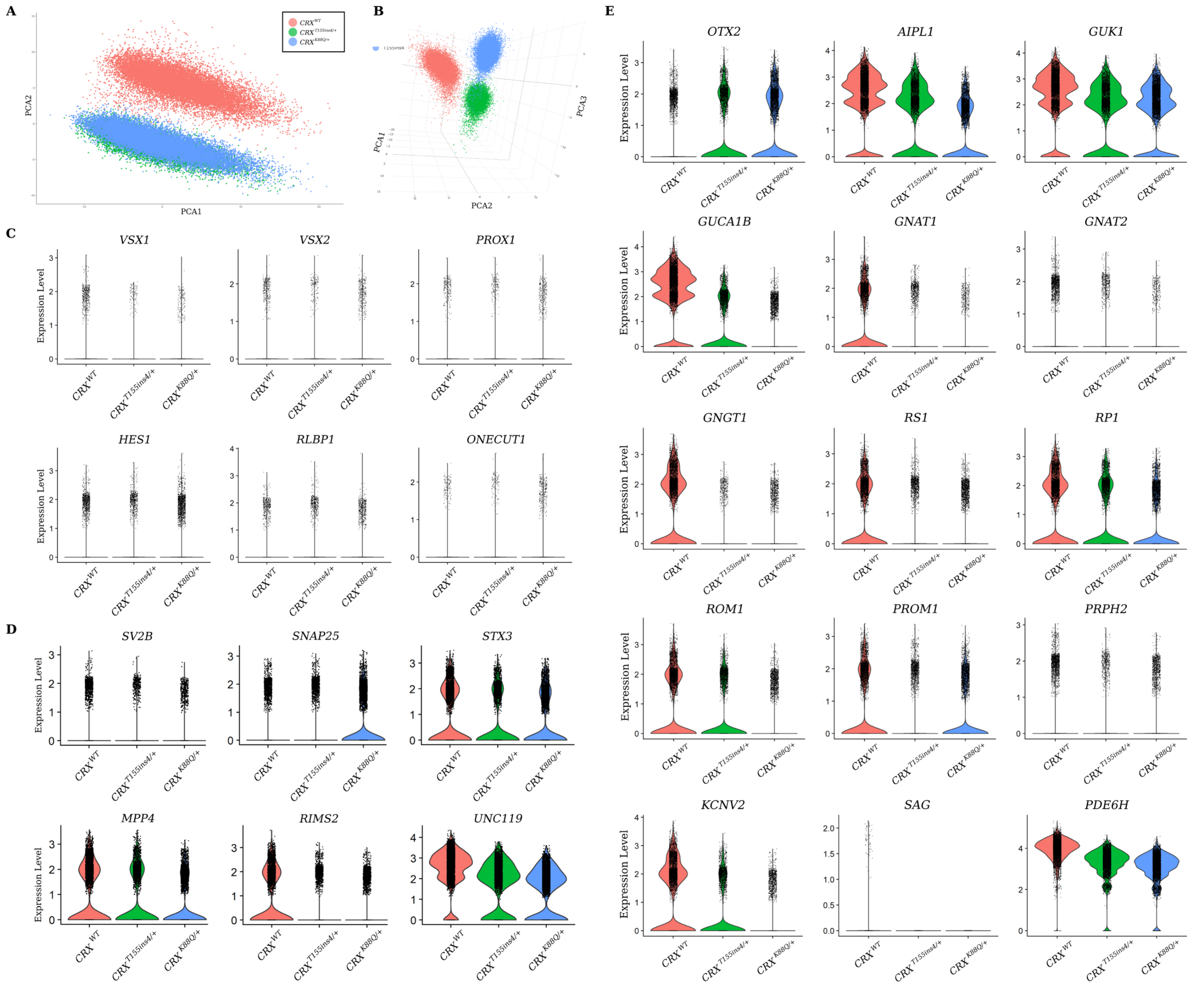

Figure S5. Single-cell RNA sequencing of D150 reveals distinct clusters between control and LCA7 organoid samples but little to no differences in non-photoreceptor cell markers, *related to Figure 2 and Figure 6*.

2D (**A**) and 3D (**B**) PCA plots show the distinct clusters for control (CRX^{WT}, red), $CRX^{T155ins4/+}$ (green), and $CRX^{K88Q/+}$ (blue) samples. Expression levels for bipolar cell (*VSX1*, *VSX2*), amacrine cell (*PROX1*), retinal progenitor cell/Müller glia (*HES1*, *RLBP1*), and retinal ganglion/horizontal cell (*ONECUT1*) transcript are shown for CRX^{WT} (red), $CRX^{T155ins4/+}$ (green), and $CRX^{K88Q/+}$ (blue) using violin plots (**C**). Expression data for vesicle markers (*SV2B*, *SNAP25, STX3*) and CAZ markers (*MPP4*, RIMS2, *UNC119*) are shown as violin plots (**D**) for CRX^{WT} (red), $CRX^{T155ins4/+}$ (green), and $CRX^{K88Q/+}$ (blue). Photoreceptor cell-specific gene expression levels are compared for CRX^{WT} (red), $CRX^{T155ins4/+}$ (green), and $CRX^{K88Q/+}$ (blue) using violin plots (**E**).

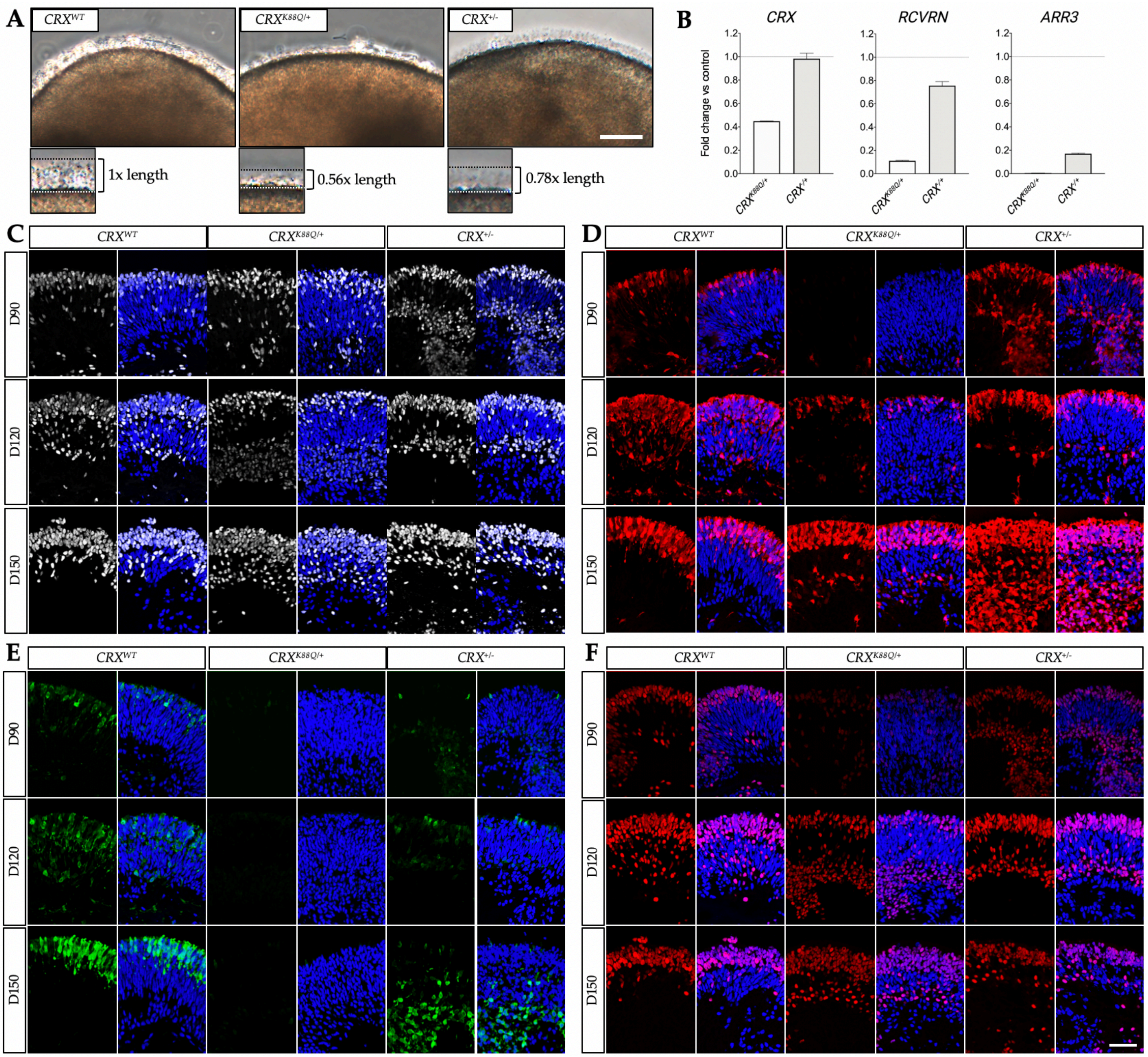

Figure S6. Analyses of *CRX*$^{+/-}$ retinal organoids at D90, D120, D150, and D180, *related to Figure 7*.
Outer segment length measurements for *CRX*$^{+/+}$, *CRX*$^{K88Q/+}$, and *CRX*$^{+/-}$ retinal organoids at D180 are shown in panel **A**. Preliminary qPCR data (n=6 organoids per sample from a single batch; statistics were performed on technical replicates) is shown at D180 for *CRX*$^{+/+}$, *CRX*$^{K88Q/+}$, and *CRX*$^{+/-}$ retinal organoids (**B**). Immunofluorescence staining with antibodies against OTX2 (white, **C**), RCVRN (red, **D**), ARR3 (green, **E**), and CRX (red, **F**) are shown for *CRX*$^{+/+}$, *CRX*$^{K88Q/+}$, and *CRX*$^{+/-}$ retinal organoids at D90, D120, and D150. Nuclei were counterstained with DAPI (blue, **C-F**). Scale bars (**A**&**F**) = 100μm.

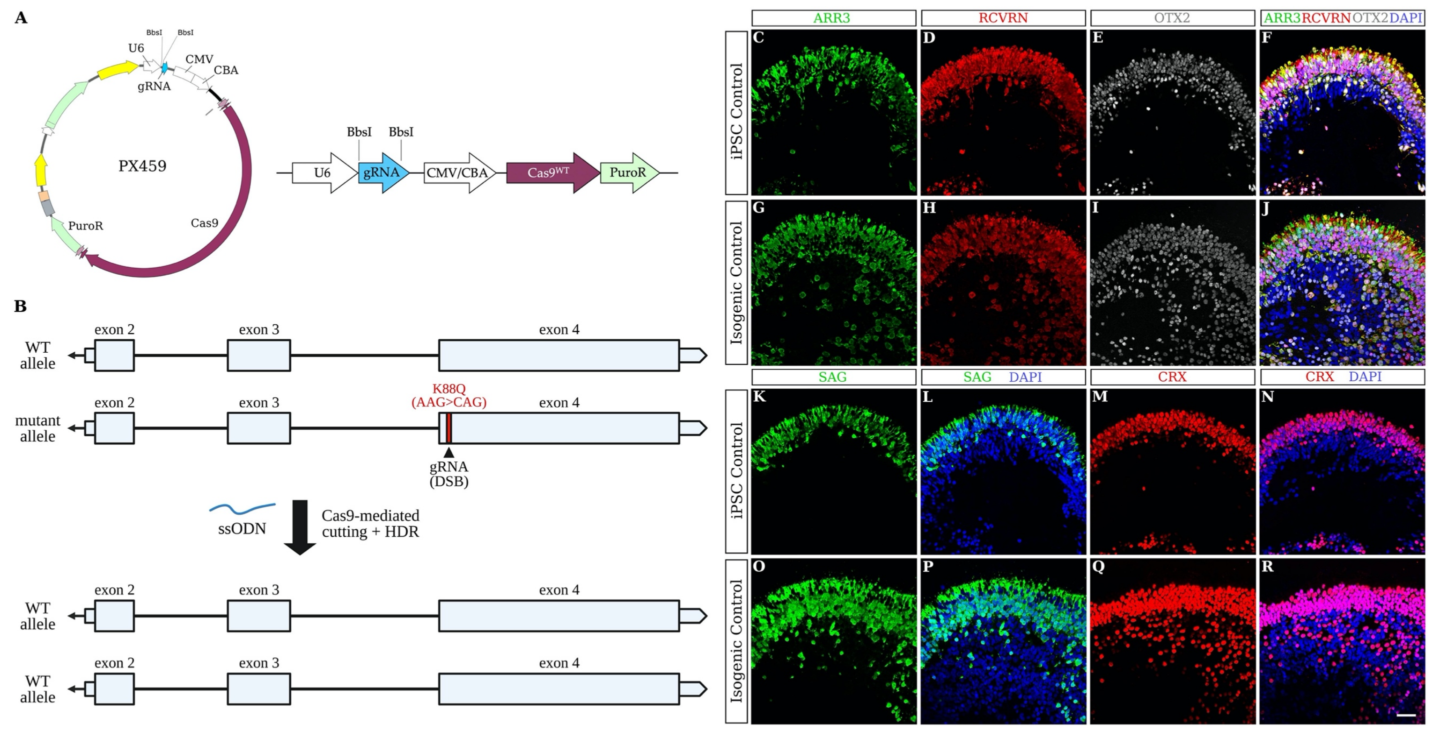

Figure S7. Generation of isogenic control hiPSC line and immunofluorescence in isogenic versus hiPSC control organoids at D180 of differentiation, *related to Figure 3, Figure 4, and Figure 7*.

To ensure our control hiPSC line is relevant in these studies, an isogenic control line was made for the *CRX*$^{K88Q/+}$ hiPSCs using CRISPR/Cas9 tools. A circular and linearized map of the PX459 plasmid is shown in **A**. The CRISPR/Cas9 target site is mapped onto the mutant allele of the *CRX* gene at the *K88Q* mutation (**B**). Immunofluorescence staining using antibodies against ARR3 (green; **C**, **F**, **G**, **J**), RCVRN (red; **D**, **F**, **H**, **J**), OTX2 (white; **E**, **F**, **I**, **J**), SAG (green; **K**, **L**, **O**, **P**), and CRX (red; **M**, **N**, **Q**, **R**) are shown for the hiPSC control (**C-F**, **K-N**) and the isogenic control (**G-J**, **O-R**) retinal organoids at D180. Nuclei are counterstained with DAPI (blue). Scale bar (**R**) = 100μm.

Table S1. Patient donor information.

Sex	Ocular History	Visual Acuity	Age at time of blood collection	Variant
Female	LCA (nystagmus at birth); keratoconus with corneal scarring, cataracts	Bare light perception	44 y	*CRX*^{T155ins4}
Female	LCA (nystagmus at birth, retinal degeneration at 4mo)	Bare light perception	20 y	*CRX*^{T155ins4}
Female	LCA (nystagmus at 2 mo, severe retinal degeneration by 1yr)	Bare light perception	3 y	*CRX*^{K88Q}

Table S2. Oligonucleotide (oligo) sequences.

Name	Oligo sequence [PAM sequence]	Oligo size (bp)
gRNA K88Q	TGCATTTAGCCCTCCGGTTC [TGG]	20
gRNA SNP 1	GAGCCATTGTGCCTAGGCCC [GGG]	20
CRX^{K88Q} ssODN HDR construct	GCTCTCCTGGGCCTCTTCCCCACTTACCCACCCCCATCTCCGCT CTTATCCCCCAGGTTTGGTTCAAGAACCGGAGGGCTAAATGCAG GCAGCAGCGACAGCAGCAGAAACAGCAGCAGCAGCC	128

Table S3. Antibodies for immunofluorescence staining.

Target	Species	Manufacturer/Vendor	Catalog #	Dilution
AIPL1	Rabbit	Gift from Ramamurthy Lab, WVU	n/a	1:150
Blue cone opsin	Rabbit	Millipore	AB5407	1:50
Calbindin	Rabbit	Chemicon/Millipore	AB1778	1:200
CHX10/VSX2	Mouse	SCBT	sc-365519	1:50
Cone arrestin (7G6)	Mouse	Gift from Peter MacLeish Lab	n/a	1:100
CRX	Mouse	SCBT	sc-377138	1:50
CRX	Rabbit	Abcam	ab140603	1:100
Green/red cone opsin	Rabbit	Gift from Jeremy Nathans Lab	n/a	1:100
GFAP	Goat	Abcam	ab53554	1:500
Glutamine synthetase	Rabbit	Abcam	ab49873	1:2500
ISLET1/2	Mouse	DSHB	39.4D5	1:25
NANOG (lot 8)	Rabbit	Cell Signaling	4903	1:250
NRL	Goat	R&D	AF2945	1:100
OCT3/4	Mouse	SCBT	sc-5279	1:100
OTX2	Goat/Biotin	R&D	BAF1979	1:250
PKCa	Rabbit	Cell Signaling	2056T	1:200
PNR/NR2E3	Mouse	R&D	PP-H7223-00	1:25
Recoverin	Rabbit	Millipore	AB5585	1:600
Rhodopsin	Mouse	Millipore	MABN15MI	1:25
SOX2	Goat	R&D	AF2018	1:100
SV2	Mouse	DSHB	SV2	1:100
VGLUT1	Guinea Pig	Chemicon/Millipore	AB5905	1:250
Rod arrestin/SAG	Mouse	SCBT	sc-166383	1:50
Anti-mouse (AlexaFluor 488)	Donkey	Life Technologies	A-21202	1:200
Anti-rabbit (AlexaFluor 555)	Donkey	Life Technologies	A-31572	1:200
Anti-guinea pig (AlexaFluor 594)	Goat	Life Technologies	A-11076	1:200
Anti-goat (AlexaFluor 647)	Donkey	Life Technologies	A-21447	1:200

Table S4. qPCR and RT-PCR Primers.

Target mRNA	Forward primer (5'→3')	Reverse primer (5'→3')	Amplicon size (bp)
ACTB	GGATCAGCAAGCAGGAGTAT	GGTGTAACGCAACTAAGTCATAG	90
AIPL1	GGATCCCGAGTGATCTTTCATT	CAGACCTCGAGCTTGAACAT	128
ARR3	CAGGAATTCACGGAGACTGTAG	GGCCAGGTTGGTATCTTCAT	138
BCL2	GGATGCCTTTGTGGAACTG	CAGCCAGGAGAAATCAAACAG	61
CASP3	CTTCTTTAGAAACATCACGCATCAA	ACCTCAGGGAAACATTCAGAAA	102
CRX	CTGTTTGCCAAGACCCAGTA	CAAACCTGAACCCTGGACT	89
NR2E3	TGATGTCACCAGCAATGACC	TCTTCCAGCAGGATCACCT	180
NRL	GCCCAGTCCCTGTTGATG	CGCAGCTGCCGGTTTAG	142
OPN1SW	CTTCCGCTTCAGCTCCAA	GAACCGGCTCCAGCCA	100
OPN1MW	TGGTCTCTGGCCATCATTTC	AGTACCTGCTCCAACCAAAG	160
OPN1LW	CACCTTCTTCGCATGCTTTG	TCGAAACTGCCGGTTCATAA	139
OTX2	AGGGTGCAGGTATGGTTTAAG	CGAGCTGGAGATGTCTTCTTT	116
P53	GGCCCACTTCACCGTACTAA	GTGGTTTCAAGGCCAGATGT	156
PDE6C	CGAAGAAGCCAGATCCTTATGT	TCATGTCCAGCAGTCCAATG	142
RCVRN	GCTCCTTCCAGACGATGAAA	TTGGCATCAGGCGTTCTT	193
RHO	ACCCTGGGCGGTGAAAT	ATGACCCAGGTGAAGGCAA	137
VSX2	GGAGAAGGCATTCAACGAAGCC	ACTTGGCTCGACGGTTCTGGAA	119
SOX2	CGTTCATCGACGAGGCTAAG	CCGCTCGCCATGCTATT	160
NANOG	CCCAGCTGTGTGTACTCAAT	TTCACCTGTTTGTAGCTGAGG	106
OCT4	TGGGAAGGTATTCAGCCAAAC	CTCTCACTCGGTTCTCGATACT	203
CD11B	CAAAGTGGTACGAGAGCTGTTT	CGAATGACTCCCTCTCTGTCT	150

Table S5. Single-cell RNA sequencing-based gene expression data for key photoreceptor markers in *CRX*$^{T155ins4/+}$ and *CRX*$^{K88Q/+}$ organoids in comparison to control organoids.

		***CRX*$^{T155ins4/+}$**		***CRX*$^{K88Q/+}$**	
**Gene**	**Localization**	**Adj P-value**	**Avg Log_2 Fold Change**	**Adj P-value**	**Avg Log_2 Fold Change**
AIPL1	Rods & Cones	$p < 1x10^{-217}$	-0.71	$p < 1x10^{-300}$	-1.67
CRX	Rods & Cones	$p < 1x10^{-126}$	0.48	$p < 1x10^{-65}$	-0.38
GUCA1B	Rods & Cones	$p < 1x10^{-300}$	-1.89	$p < 1x10^{-300}$	-2.67
GUK1	Rods & Cones	$p < 1x10^{-268}$	-0.76	$p < 1x10^{-300}$	-0.73
KCNV2	Rods & Cones	$p < 1x10^{-270}$	-1.08	$p < 1x10^{-300}$	-1.88
OTX2	Rods & Cones	$p < 1x10^{-64}$	0.59	$p < 1x10^{-202}$	0.83
PRCD	Rods & Cones	$p < 1x10^{-134}$	-0.84	$p < 1x10^{-49}$	-0.53
PROM1	Rods & Cones	$p < 1x10^{-38}$	-0.44	$p < 1x10^{-44}$	-0.50
PRPH2	Rods & Cones	$p < 1x10^{-80}$	-0.65	$p < 1x10^{-42}$	-0.50
RCVRN	Rods & Cones	$p < 1x10^{-300}$	-1.00	$p < 1x10^{-300}$	-2.31
RP1	Rods & Cones	$p < 1x10^{-62}$	-0.50	$p < 1x10^{-249}$	-0.97
GNAT1	Rods	$p < 1x10^{-198}$	-1.05	$p < 1x10^{-300}$	-1.57
GNGT1	Rods	$p < 1x10^{-300}$	-2.11	$p < 1x10^{-300}$	-2.00
NRL	Rods	$p < 1x10^{-117}$	-0.48	$p < 1x10^{-17}$	-0.19
PDE6G	Rods	$p < 1x10^{-66}$	-0.54	$p < 1x10^{-59}$	-0.50
ROM1	Rods	$p < 1x10^{-81}$	-0.62	$p < 1x10^{-288}$	-1.17
SAG	Rods	$p < 1x10^{-06}$	-0.06	$p < 1x10^{-14}$	-0.07
ARR3	Cones	$p < 1x10^{-300}$	-2.43	$p < 1x10^{-300}$	-2.93
GNAT2	Cones	$p < 1x10^{-44}$	-0.47	$p < 1x10^{-167}$	-0.87
OPN1SW	Cones	n.s.	-0.04	$p < 1x10^{-12}$	-0.08
PDE6C	Cones	$p < 1x10^{-14}$	-0.21	$p < 1x10^{-36}$	-0.28
PDE6H	Cones	$p < 1x10^{-300}$	-0.93	$p < 1x10^{-300}$	-1.20
BSN	Ribbon Synapse	n.s.	0.07	n.s.	0.04
CTBP2	Ribbon Synapse	n.s.	0.06	n.s.	-0.17
SNAP25	Synaptic Vesicles	n.s.	0.02	$p < 1x10^{-22}$	0.26
STX3	Synaptic Vesicles	$p < 1x10^{-33}$	-0.38	$p < 1x10^{-35}$	-0.40
SV2B	Synaptic Vesicles	$p < 1x10^{-22}$	-0.33	$p < 1x10^{-41}$	-0.45
MPP4	CAZ	$p < 1x10^{-27}$	-0.34	$p < 1x10^{-144}$	-0.80
RIMS2	CAZ	$p < 1x10^{-122}$	-0.78	$p < 1x10^{-261}$	-1.11
UNC119	CAZ	$p < 1x10^{-300}$	-0.91	$p < 1x10^{-300}$	-1.31
RS1	Synaptic Cleft	$p < 1x10^{-145}$	-0.84	$p < 1x10^{-216}$	-1.01

n.s. = no significance

Table S6. Raw single-cell RNA sequencing data file.
p_val = p-value; avg_log2FC = average $\log_2$ fold change; pct.1 = percentage of cells in which the gene is detected in the first group; pct.2 = percentage of cells in which the gene is detected in the second group; p_val_adjusted = adjusted p-value

Supplemental Experimental Procedures

PBMC Processing and Reprogramming

Whole blood samples were collected in heparinized tubes and processed immediately after collection. Peripheral blood mononuclear cells (PBMCs) were isolated from whole blood samples using a centrifugation method as follows. Whole blood was diluted 1:1 with 1X Dulbecco's Phosphate Buffered Saline (DPBS, Fisher Scientific, MT21031CV) without calcium or magnesium, and 7 mL of diluted blood was added on top of 3 mL of Ficoll-Paque Plus medium (VWR, 95021-205). Samples were then centrifuged for 30 minutes at 400 x g. PBMCs were then collected from the cloudy white interface between the plasma and the Ficoll-Paque. 2 x 10^6 PBMCs were plated into a single well of a 12-well tissue culture plate in Expansion Medium: StemPro-34 SFM (Fisher Scientific, 10639011), hSCF (100ng/mL; PeproTech, 300-07), FLT3L (100ng/mL; PeproTech, 300-19), IL-3 (20ng/mL; Sigma, I1646), IL-6 (20ng/mL; BioLegend, 570802). Media was changed every 2 days to fresh Expansion Medium.

For reprogramming, PBMCs were transduced using the CytoTune™-iPS 2.0 Sendai Reprogramming Kit (ThermoFisher Scientific, A16517) at an MOI of 5, based on cell number according to manufacturer instructions for lot# L2150040. Briefly, PBMCs were resuspended in fresh Expansion Medium containing the appropriate amount of virus and spun down for 30 minutes at 2000rpm before briefly resuspending cells and plating directly into a 24-well plate pre-coated with vitronectin XF (STEMCELL Technologies, 7180). After 16-18 hours, virus-containing media was replaced with fresh Expansion Medium. On day 3 post-transduction, Expansion Medium was replaced with Reprogramming Medium: StemPro-34 SFM (Fisher Scientific, 10639011), hSCF (100ng/mL; PeproTech, 300-07), FLT3L (100ng/mL; PeproTech, 300-19). Starting on day 6 post-transduction, cells were given a half medium change with mTeSR™1 medium (STEMCELL Technologies, 85850) + 0.25mM sodium butyrate (NaB; SelleckChem, S1999) every day until induced pluripotent stem cell (iPSC) colonies began to appear. NaB was removed from the media starting on day 8. Newly formed iPSC colonies were picked off the plate using a P200 pipette tip and transferred to a 6-well plate coated with Matrigel (Corning, 354234). Newly picked iPSC clones were passaged at least 10 times until a clean, fully reprogrammed clone was established and verified by immunocytochemistry and RT-PCR. Passaging was conducted by gently scraping cells off the plate after incubating cells for 2 minutes at room temperature in a solution of EDTA (1:1000; Corning, 46-034-CI) in 1X DPBS. Human iPSC colonies were maintained in mTeSR™ Plus medium (STEMCELL Technolognies, 5825) with media changes occurring every other day. The control hiPSC line (CRX^{WT}, also known as "LiPSC-ER2.2") used throughout this work was originally generated from human umbilical cord blood in a cGMP facility at Lonza Walkersville, Inc. More information on how this line was generated can be found in Baghbaderani et al., 2015.

Reverse transcription polymerase chain reaction (RT-PCR)

To confirm pluripotency in newly reprogrammed hiPSC clones, total RNA was extracted using the RNeasy Extraction Kit (Qiagen, 74104), and cDNA was then generated using the iScript cDNA Synthesis Kit (Bio-Rad, 1708891). RT-PCR using the Taq DNA Polymerase kit (ThermoFisher Scientific, EP0402) was performed to look for mRNA expression of standard pluripotency markers *SOX2*, *OCT4*, and *NANOG*, as well as loss of *CD11B* (PBMC marker) expression. The reactions were then run on a 1% agarose gel to look for the presence of a band at the appropriate size.

Thermal cycling protocol for RT-PCR:

Initial Denaturation	Denaturation	Annealing	Extension	Final Extension
95°C	95°C	56°C	72°C	72°C
3:00	0:30	0:30	0:20	5:00
	Repeat for 34 cycles			

CRX gene sequencing

The thermal cycling protocol used to amplify *CRX* for sequencing applications:

Initial Denaturation	Denaturation	Annealing	Extension	Final Extension
95°C	95°C	56°C	72°C	72°C

3:00	0:30	0:30	0:45	5:00
	Repeat for 34 cycles			

Generating an isogenic control hiPSC line

All CRISPR-Cas9-mediated gene editing in hiPSCs was performed using the following protocol. Stable hiPSC colonies were grown in a 12-well TC-treated cell culture plate in mTeSR™ Plus medium until they reached ~40-50% confluency. The culture medium was then replaced with Opti-MEM™ I Reduced Serum Medium (Gibco, 31985062) 1 hour prior to transfection. To transfect the cells, Lipofectamine^{TM} Stem Transfection Reagent (ThermoFisher Scientific, STEM00008) was used at a concentration of 1µL/500ng of total DNA, following manufacturer's instructions. To generate the isogenic control line, hiPSCs containing the *CRX*$^{K88Q/+}$ genotype were transfected with the PX459-Cas9 plasmid containing an gRNA directly targeting the K88Q mutation (5µg) along with a single-stranded oligodeoxynucleotide (ssODN) construct (synthesized by Integrated DNA Technologies) as a homology-directed repair (HDR) template (500ng). On day 2-5 post-transfection, cells were treated with puromycin (0.25µg/mL) to select for cells that took up the PX459-Cas9 plasmid. After treatment, individual cells were left to grow until they could be split to propagate individual clones. To confirm correction of the *CRX* gene, individual clones were assessed by PCR amplification followed by Sanger sequencing. Oligo sequences can be found in **Table S2**.

Retinal organoid differentiation

Colonies used to make retinal organoids were transferred to a 6-well suspension plate in a solution of 3 parts mTeSR™ Plus medium and 1 part Neural Induction Medium (NIM); this is considered "day 0" of differentiation. To continue driving the hiPSCs toward a neural fate, NIM was slowly increased each day as follows: day 0 = 3:1 of mTeSR™ Plus:NIM, day 1 = 1:1 of mTeSR™ Plus:NIM, day 2 = 1:3 of mTeSR™ Plus:NIM, day 3 = complete NIM. Media was changed to fresh NIM on day 5, followed by fresh NIM + 1.5nM BMP-4 (PeproTech, 120-05ET) on day 6. On day 7, the 3D embryoid bodies (EBs) were transferred to a Matrigel-coated plate with fresh NIM medium + 1.5nM BMP-4, to allow EBs to adhere to the plate. Adherent EBs were fed as follows: day 8 = NIM + 1.5nM BMP-4, day 9 = NIM + 0.75nM BMP-4, day 11 = NIM + 0.75nM BMP-4, day 12 = NIM + 0.375nM BMP-4, day 14 = NIM + 0.375nM BMP-4, day 15 = NIM only. From day 16 through day 29, the EBs were fed Retinal Differentiation Medium (RDM) and regions of the plate that began to develop clear retinal morphology during this time were lifted off using a P1000 pipette tip. Lifted neural retina was transferred to a suspension plate and allowed to resume a 3D organoid structure. From day 30 to day 120, the developing retinal organoids were fed 3D-RDM medium and maintained in suspension. Beyond day 120, organoids were given 3D-RDM medium without added retinoic acid (ATRA).

Media formulations for retinal differentiation protocol:

**Neural Induction Medium (NIM)**	**Manufacturer/Vendor**	**Catalog #**	**Final Concentration**
DME/F12	HyClone	SH30023.02	
MEM nonessential amino acid solution (NEAA)	Corning	25025CI	1X
GlutaMAX™ Supplement	Gibco	35050061	1X (2mM)
Heparin sulfate	Sigma	H3393	2µg/mL
Penicillin/Streptomycin	Corning	30001CI	1%
N-2 Supplement	Gibco	17502001	1X

**Retinal Differentiation Medium (RDM)**	**Manufacturer/Vendor**	**Catalog #**	**Final Concentration**
DME/F12	HyClone	SH30023.02	1 part
DMEM High Glucose	HyClone	SH30022.02	1 part
MEM nonessential amino acid solution (NEAA)	Corning	25025CI	1X
GlutaMAX™ Supplement	Gibco	35050061	1X (2mM)
Penicillin/Streptomycin	Corning	30001CI	1%
B-27™ Supplement	Gibco	17504001	1X

**3D-Retinal Differentiation Medium (3D-RDM)**	**Manufacturer/Vendor**	**Catalog #**	**Final Concentration**
DME/F12	HyClone	SH30023.02	1 part
DMEM High Glucose	HyClone	SH30022.02	1 part

Fetal Bovine Serum	R&D	S11150	5%
GlutaMAX™ Supplement	Gibco	35050061	1X (2mM)
Penicillin/Streptomycin	Corning	30001CI	1%
Taurine	Sigma	T0625	200µM
Chemically Defined Lipid Concentrate	Gibco	11905031	1:1000
B-27™ Supplement	Gibco	17504001	1X
Retinoic acid (ATRA)	Sigma	R2625	1µM

Immunocytochemistry

Each newly reprogrammed hiPSC clone was grown on a 24-well TC-treated cell culture plate to reach ~50-70% confluency. The cells were then fixed on the plate for 10 minutes at room temperature using 4% PFA prior to staining. After rinsing three times with 1X PBS, non-specific binding was blocked using 10% normal donkey serum (NDS; MilliporeSigma, S30-100ML) in 1X PBS with 0.1% Triton X-100 (VWR, 0694) for 15 minutes at room temperature. The cells were then incubated for 1 hour at room temperature with primary antibodies targeting SOX2, NANOG, and OCT4 (see **Table S3** for full list of antibodies). After washing cells three times with 1X PBS, a secondary antibody solution was added to the cells for 30 minutes at room temperature, protected from light. Finally, cells were counterstained with 4′,6-diamidino-2-phenylindole (DAPI, 1µg/mL; Roche, 10236276001) for 2 minutes, before being imaged on an Olympus BX51 microscope in 1X PBS.

Immunofluorescence

Retinal organoids were collected at D75, D90, D120, D150, D180, and D240 (n=3/clone per collection, with 3 separate collections per time point) for each genotype. Organoids were fixed for 30 minutes in 4% PFA, followed by exposure to a series of sucrose solutions in 1X PBS: 10% sucrose for 15 minutes, 15% sucrose for 30 minutes, then 20% sucrose for 3 hours. Organoids were then flash frozen in a 2:1 mixture of 20% sucrose and Optimal Cutting Temperature compound (O.C.T.; VWR, 25608-930). 7µm-thick sections were collected from the cryoblocks for immunofluorescence staining. Sections were incubated in 10% NDS for 15 minutes, followed by a 2-hour incubation with primary antibodies at room temperature (see **Table S3** for full list of antibodies). After rinsing three times with 1X PBS, sections were incubated with the corresponding secondary antibodies for 30 minutes in the dark at room temperature. DAPI solution (1µg/mL) was then applied to each section for 2 minutes in the dark, before rinsing sections three times with 1X PBS. Finally, a coverslip was applied to each slide using Fluoromount-G™ Slide Mounting Medium (Electron Microscopy Sciences, 17984-25). Confocal images were captured using an LSM700 microscope with a 40X lens.

Quantitative real-time PCR (qPCR) analysis

For qPCR experiments, the following thermal cycling protocol was used on a Bio-Rad CFX Connect Real-Time PCR System:

Polymerase activation & initial denaturation	Denaturation	Annealing/ Extension	Melt Curve Analysis
95°C	95°C	58°C	65°C-95°C at 0.5°C increments
0:30	0:10	0:30	0:05/step
	Repeat for 39 cycles		

Fluorescence Intensity analysis

Fluorescence intensity measurements were collected for n=3 organoids per marker per line using FIJI. Measurements were first normalized to DAPI intensity measurements, and then normalized to control. All data summary graphs were made using GraphPad Prism 8, and all statistical analyses were performed via one-way ANOVA with a Dunnett test to correct for multiple comparisons.

Resource Availability

Further information and requests for resources and reagents should be directed to and will be fulfilled by the Corresponding Author, Deepak Lamba (Deepak.Lamba@ucsf.edu). All unique/stable reagents generated in this study are available from Dr. Lamba with a completed Materials Transfer Agreement. The published article includes all scRNAseq datasets generated or analyzed during this study.

Supplemental References

Baghbaderani, B.A., Tian, X., Neo, B.H., Burkall, A., Dimezzo, T., Sierra, G., Zeng, X., Warren, K., Kovarcik, D.P., Fellner, T., et al. (2015). cGMP-Manufactured Human Induced Pluripotent Stem Cells Are Available for Pre-clinical and Clinical Applications. Stem Cell Reports *5*, 647–659. 10.1016/j.stemcr.2015.08.015

Cao, J., Spielmann, M., Qiu, X., Huang, X., Ibrahim, D.M., Hill, A.J., Zhang, F., Mundlos, S., Christiansen, L., Steemers, F.J., et al. (2019). The single-cell transcriptional landscape of mammalian organogenesis. Nature *566*, 496–502. 10.1038/s41586-019-0969-x

Haghverdi, L., Lun, A.T.L., Morgan, M.D., and Marioni, J.C. (2018). Batch effects in single-cell RNA-sequencing data are corrected by matching mutual nearest neighbors. Nat Biotechnol *36*, 421–427. 10.1038/nbt.4091

Stuart, T., Butler, A., Hoffman, P., Hafemeister, C., Papalexi, E., Mauck, W.M., Hao, Y., Stoeckius, M., Smibert, P., and Satija, R. (2019). Comprehensive Integration of Single-Cell Data. Cell *177*, 1888-1902.e21. 10.1016/j.cell.2019.05.031

Yousif, A., Drou, N., Rowe, J., Khalfan, M., and Gunsalus, K.C. (2020). NASQAR: a web-based platform for high-throughput sequencing data analysis and visualization. BMC Bioinformatics *21*, 267. 10.1186/s12859-020-03577-4
