# Supplementary material for: Allele-specific gene editing to rescue dominant CRX-associated LCA7 phenotypes in a retinal organoid model
Source: Stem Cell Reports. 2021 Oct 14;16(11):2690–702. doi: 10.1016/j.stemcr.2021.09.007 (PMC8580887; doi:10.1016/j.stemcr.2021.09.007)
Supplement: Document S2. Article plus supplemental information [file mmc2.pdf]

Stem Cell Reports
Article
OPEN ACCESS

Allele-specific gene editing to rescue dominant *CRX*-associated LCA7 phenotypes in a retinal organoid model

Kathleen R. Chirco,[1,2,3,4] Shereen Chew,[1,2] Anthony T. Moore,[1] Jacque L. Duncan,[1] and Deepak A. Lamba[1,2,*]
[1]Department of Ophthalmology, University of California San Francisco, San Francisco, CA, USA
[2]The Eli and Edythe Broad Center of Regeneration Medicine and Stem Cell Research, University of California San Francisco, San Francisco, CA, USA
[3]Divison of Neuroscience, Oregon National Primate Research Center, Oregon Health & Science University, Beaverton, OR, USA
[4]Casey Eye Institute, Oregon Health & Science University, Portland, OR, USA
*Correspondence: deepak.lamba@ucsf.edu
https://doi.org/10.1016/j.stemcr.2021.09.007

SUMMARY

Cases of Leber congenital amaurosis caused by mutations in *CRX* (LCA7) exhibit an early form of the disease and show signs of significant photoreceptor dysfunction and eventual loss. To establish a translational *in vitro* model system to study gene-editing-based therapies, we generated LCA7 retinal organoids harboring a dominant disease-causing mutation in *CRX*. Our LCA7 retinal organoids develop signs of immature and dysfunctional photoreceptor cells, providing us with a reliable *in vitro* model to recapitulate LCA7. Furthermore, we performed a proof-of-concept study in which we utilize allele-specific CRISPR/Cas9-based gene editing to knock out mutant *CRX* and saw moderate rescue of photoreceptor phenotypes in our organoids. This work provides early evidence for an effective approach to treat LCA7, which can be applied more broadly to other dominant genetic diseases.

INTRODUCTION

Leber congenital amaurosis (LCA) is a group of early-onset inherited retinal diseases that accounts for approximately 5% of all inherited retinopathies (Koenekoop, 2004; Stone, 2007). Although LCA patients can exhibit a range of disease severity, they commonly exhibit poor vision from infancy, nystagmus, and substantially reduced or non-recordable electroretinogram (ERG) responses (Kumaran et al., 2017). Aside from these core characteristics, variable disease phenotypes can exist among LCA patients, due to disease-causing variants occurring in at least 25 genes (den Hollander et al., 2008; Thompson et al., 2018). While most of these variants are inherited in an autosomal recessive fashion, LCA-causing mutations in the *CRX* gene are typically autosomal dominant. The CRX (cone-rod homeobox) protein is a transcription factor expressed early in newly differentiated photoreceptor cells during retinal development. In addition to autoregulation, CRX plays a critical role in driving expression of genes required for photoreceptor maturation and function, as well as cytoskeletal matrix of the active zone (CAZ) genes (Corbo et al., 2010; Hennig et al., 2008).

CRX-associated LCA (LCA7) accounts for roughly 2% of all LCA cases (Hull et al., 2014; Stone, 2007) and represents an especially devastating form of the disease, likely due to incomplete formation of photoreceptor outer segments, as has been reported in an LCA7 mouse model (Tran et al., 2014). Despite the presence of immature photoreceptor cells, preservation of the outer nuclear layer (ONL) is visible for some time in mice and in patients with LCA7 (Tran et al., 2014, Figure S1). While we know that disease-causing mutations in *CRX* have a significant impact on photoreceptor maturation and function, no treatments currently exist for LCA7. Therefore, novel therapeutic approaches must be developed to treat this dominant form of the disease.

Advancements in the human three-dimensional (3D) retinal organoid field have provided a promising alternative to rodent models for studying genetic retinal diseases (reviewed in Artero Castro et al., 2019; Kruczek and Swaroop, 2020). Therefore, we aimed to establish an *in vitro* human retinal organoid model system to study mutation-specific LCA7 phenotypes, allowing us to visualize the initiation and progression of disease phenotypes as the retina develops. Here, we reprogrammed peripheral blood mononuclear cells (PBMCs) derived from patients with a clinical diagnosis of LCA7. Each of the two resulting human induced pluripotent stem cell (hiPSC) lines harbors a dominant disease-causing mutation in *CRX*: $CRX^{T155ins4/+}$ (c.464_465insGGCA; p.T155ins4) or $CRX^{K88Q/+}$ (c.262A>C; p.K88Q). The hiPSC lines were then differentiated to generate 3D retinal organoids to establish a human-tissue-based model of LCA7. Characterization of these organoids revealed severe photoreceptor phenotypes at the cellular and molecular levels, along with a clear defect in photoreceptor outer-segment maturation. Since CRX is believed to be largely haplosufficient (Furukawa et al., 1999; Ibrahim et al., 2018), we performed a gene-editing-based proof-of-concept study to examine our ability to rescue these phenotypes by knocking out the mutant allele in our model. We found a robust increase in key photoreceptor markers in edited ($CRX^{+/-}$) compared with unedited ($CRX^{K88Q/+}$) organoids. This work not only establishes a model system to further study LCA7

This is an open access article under the CC BY license (http://creativecommons.org/licenses/by/4.0/).

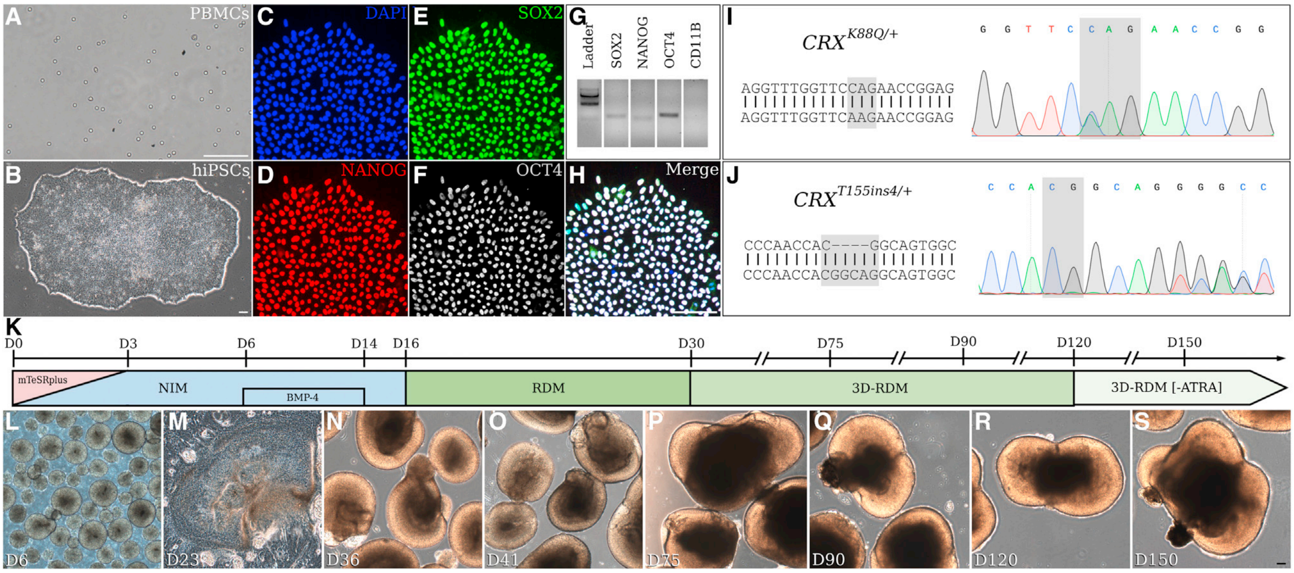

Figure 1. Characterization and differentiation of patient iPSC lines

(A–S) Patient PBMCs (A) were reprogrammed to generate stable hiPSC lines (B). Immunocytochemistry was performed with antibodies against NANOG (D), SOX2 (E) and OCT3/4 (F), and cells were counterstained with DAPI (C). The merged image is shown in (H). Primers for *SOX2*, *NANOG*, *OCT4*, and *CD11B* were utilized for RT-PCR analysis (G). Sanger sequencing results for hiPSCs to confirm the presence of the $CRX^{K88Q/+}$ (I) and $CRX^{T155ins4/+}$ (J) variants. The retinal differentiation protocol timeline is summarized in (K), and representative images of the differentiation process are shown for the control hiPSC line (CRX^{WT}) in (L)–(S). Scale bar (A, B, H, and S), 100 μm.

disease mechanisms, it also provides evidence to support the development of an allele-specific gene-editing-based therapeutic approach to treat LCA7.

RESULTS

LCA7 retinal organoids lack outer-segment-like projections

Using previously published 3D retinal organoid differentiation protocols, we generated organoids from control (CRX^{WT}) and LCA7 ($CRX^{T155ins4/+}$ or $CRX^{K88Q/+}$) hiPSC lines (Figure 1). The control (Figures 1L–1S) and LCA7 retinal organoids consistently showed comparable retinal morphology throughout differentiation up to day 150 (D150). By D180, LCA7 retinal organoids show obvious differences in the appearance of the projections that protrude from the edge of the organoids (Figures 2A–2C and S2). After further analysis using transmission electron microscopy (TEM), these projections appear to be early outer-segment-like structures in the control retinal organoids, with disc-like structures observed throughout (Figures 2D, 2G, and 2G′). For both *CRX* mutations, the retinal organoids have clear inner segments and some connecting cilia, but no outer segments were present along the outer edge of the organoids (Figures 2E, 2F, and 2H). The closest to an early outer segment we could find in any of the LCA7 organoids is shown in Figure 2H, and may simply represent the end structure of the connecting cilium, as no discs are visible. When these organoids were cultured through D240 of differentiation, the morphological defect remained visible in both of the LCA7 retinal organoids (Figure S4).

Photoreceptor maturation is disrupted in LCA7 retinal organoids

To further characterize the LCA7 organoids at the cellular and molecular levels, immunofluorescence (IF) and quantitative real-time PCR (qRT-PCR) analyses were performed at multiple time points during differentiation: D75, D90, D120, D150, and D180. These data revealed comparable levels of OTX2 and a decrease in immunolabeling of recoverin (RCVRN) and AIPL1 for both $CRX^{T155ins4/+}$ and $CRX^{K88Q/+}$ organoids (Figures 3, 4, and S3). qRT-PCR data for each of these markers was consistent with the IF findings (Figure 3Y). Total *CRX* mRNA and immunolabeling data show an increase in total CRX for the $CRX^{T155ins4/+}$ organoids, especially early in photoreceptor differentiation, and a decrease for the $CRX^{K88Q/+}$ organoids compared with control (Figures 3A–3F′ and S3). While we only saw a mild decrease in neural retina-specific leucine zipper protein (NRL) levels, we observed a robust decrease in rod arrestin (SAG), and a complete absence of rhodopsin (RHO) in both LCA7 organoids. NR2E3 appeared normal in $CRX^{T155ins4/+}$ organoids but low in $CRX^{K88Q/+}$ organoids compared with control (Figures 4 and 5A–5G′).

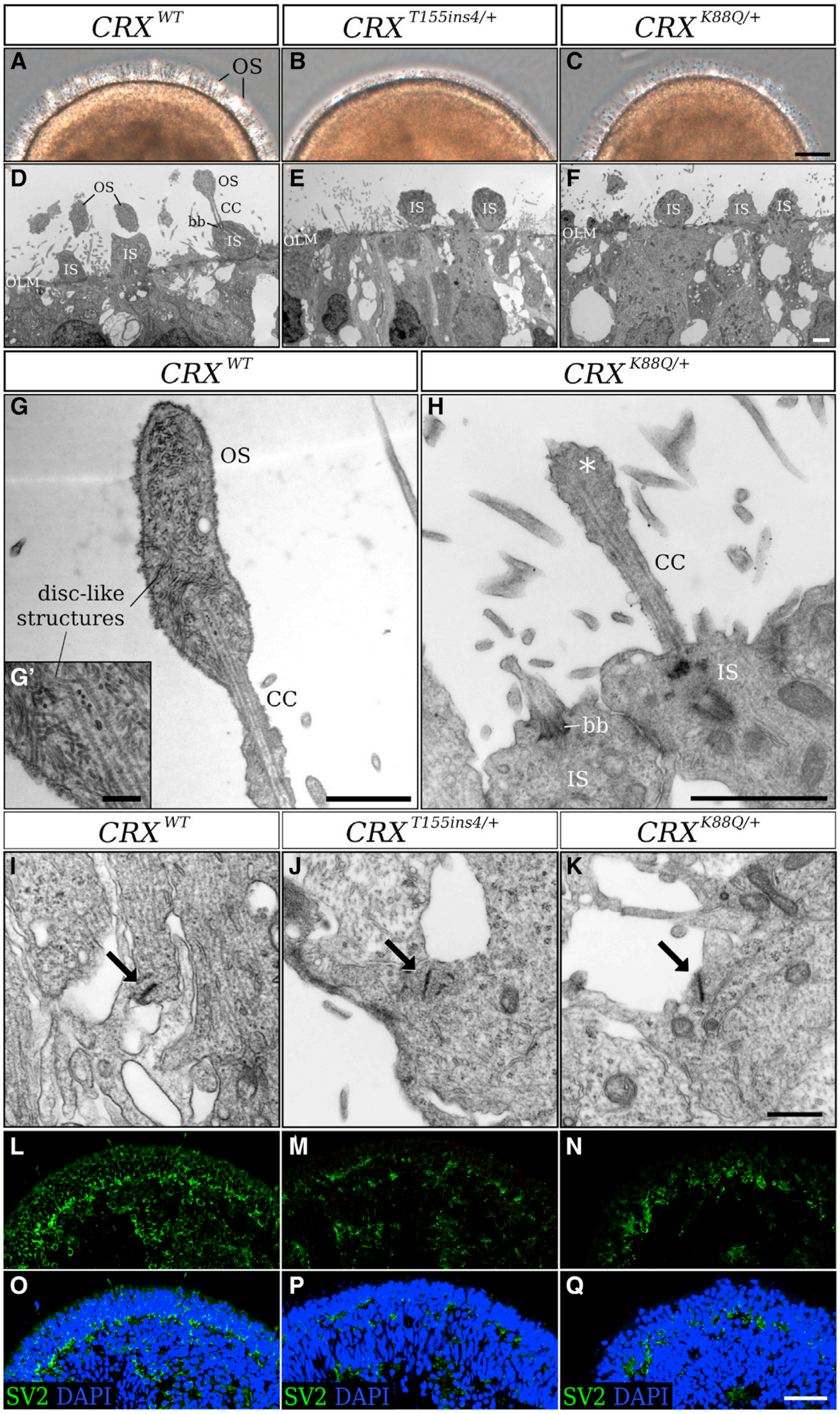

Figure 2. Altered outer-segment morphology and synapse markers in patient hiPSC-derived LCA7 retinal organoids compared with control organoids at D180

(A–C) Phase-contrast images for *CRX*^{WT} (A), *CRX*$^{T155ins4/+}$ (B), and *CRX*$^{K88Q/+}$ (C) retinal organoids at D180 of differentiation were taken along the edge of the organoids to show early outer-segment morphology.
(D–K) TEM images for *CRX*^{WT} (D), *CRX*$^{T155ins4/+}$ (E), and *CRX*$^{K88Q/+}$ (F) retinal organoids were collected at D180 (n = 1 organoid per line). Early outer segments for the *CRX*^{WT} retinal organoids show disc-like structures at D180 (G, G′), whereas *CRX*$^{K88Q/+}$ retinal organoids have underdeveloped outer-segment-like features (asterisk, H). TEM images show the formation of ribbon synapses (arrows) for *CRX*^{WT} (I), *CRX*$^{T155ins4/+}$ (J), and *CRX*$^{K88Q/+}$ (K) at D180.
(L–Q) Immunolabeling for SV2 (green) is shown for *CRX*^{WT} (L and O), *CRX*$^{T155ins4/+}$ (M and P), and *CRX*$^{K88Q/+}$ (N and Q) at D180. DAPI counterstaining is shown in blue (O–Q). Scale bar (C), 10 μm; scale bars (D–H), 5 μm; scale bar (G′), 1 μm; scale bar (K), 500 nm; scale bar (Q), 100 μm. OS, outer segment; IS, inner segment; OLM, outer limiting membrane; CC, connecting cilium; bb, basal body.
See also Figures S2 and S5.

Immunolabeling for both LCA7 organoids showed low levels of cone arrestin (ARR3), blue cone opsin (S-opsin/OPN1SW), and green and red cone opsin (M/L-opsin/OPN1MW and OPN1LW), which is supported by qRT-PCR (Figures 3 and 5). Downregulation of key photoreceptor cell markers was more prominent in the *CRX*$^{K88Q/+}$ organoids than the *CRX*$^{T155ins4/+}$ organoids for all markers except M/L-opsin, which was higher in *CRX*$^{K88Q/+}$ organoids at D180 (Figure 5).

In addition to the morphological defects, the histological phenotypes persisted out to D240 of differentiation (Figure S4). Other retinal cell types appeared unaffected by the

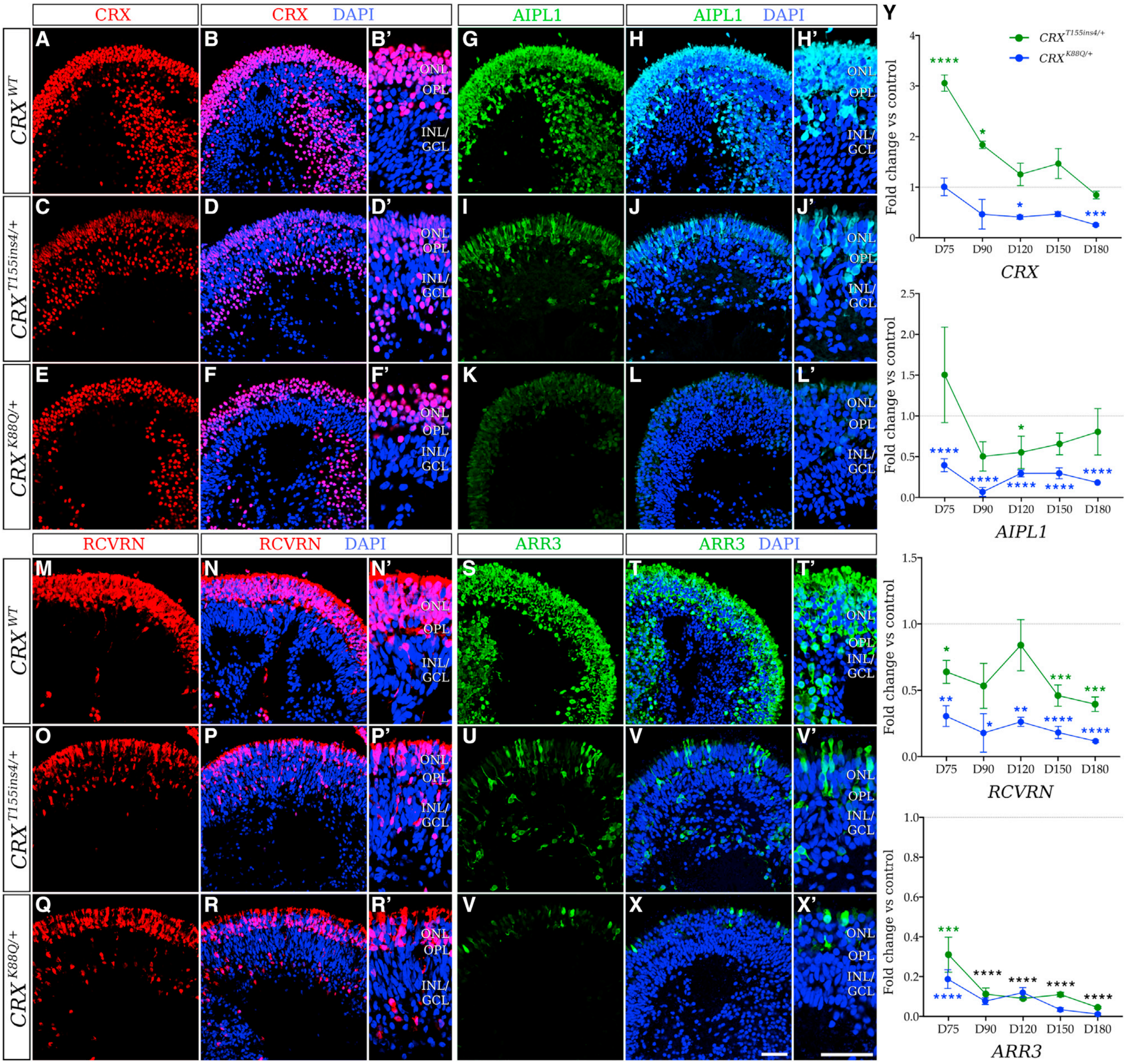

Figure 3. Altered expression of early photoreceptor cell markers in patient hiPSC-derived LCA7 retinal organoids compared with control organoids at D180

(A–Y) qRT-PCR data (I) are shown for *CRX*$^{T155ins4/+}$ (green line) and *CRX*$^{K88Q/+}$ (blue line) at D75, D90, D120, D150, and D180 as fold change compared with control organoids for *CRX*, *AIPL1*, *RCVRN*, and *ARR3* (n = 14 total organoids from two experimental replicates per line, per time point). The dotted line represents no change compared with control (y = 1). Immunofluorescence staining using antibodies against CRX (red; J–O′), AIPL1 (green; P–U′), RCVRN (red; V–AA′), and ARR3 (green; AB–AG′) are shown for control (*CRX*^{WT}; J–K′, P–Q′, V–W′, AB–AC′), *CRX*$^{T155ins4/+}$ (L–M′, R–S′, X–Y′, AD–AE′), and *CRX*$^{K88Q/+}$ (N–O′, T–U′, Z–AA′, AF–AG′) retinal organoids at D180 (n = 9 total organoids from three experimental replicates per line). Nuclei are counterstained with DAPI (blue). Scale bar (AG and AG′), 100 μm. OPL, outer plexiform layer; INL/GCL, inner nuclear layer/ganglion cell layer. *p < 0.05, **p < 0.01, ***p < 0.005, ****p < 0.001. All statistical analyses were performed using one-way ANOVA with a Dunnett test to correct for multiple comparisons in GraphPad Prism 8 software. See also Figures S3, S4, and S7.

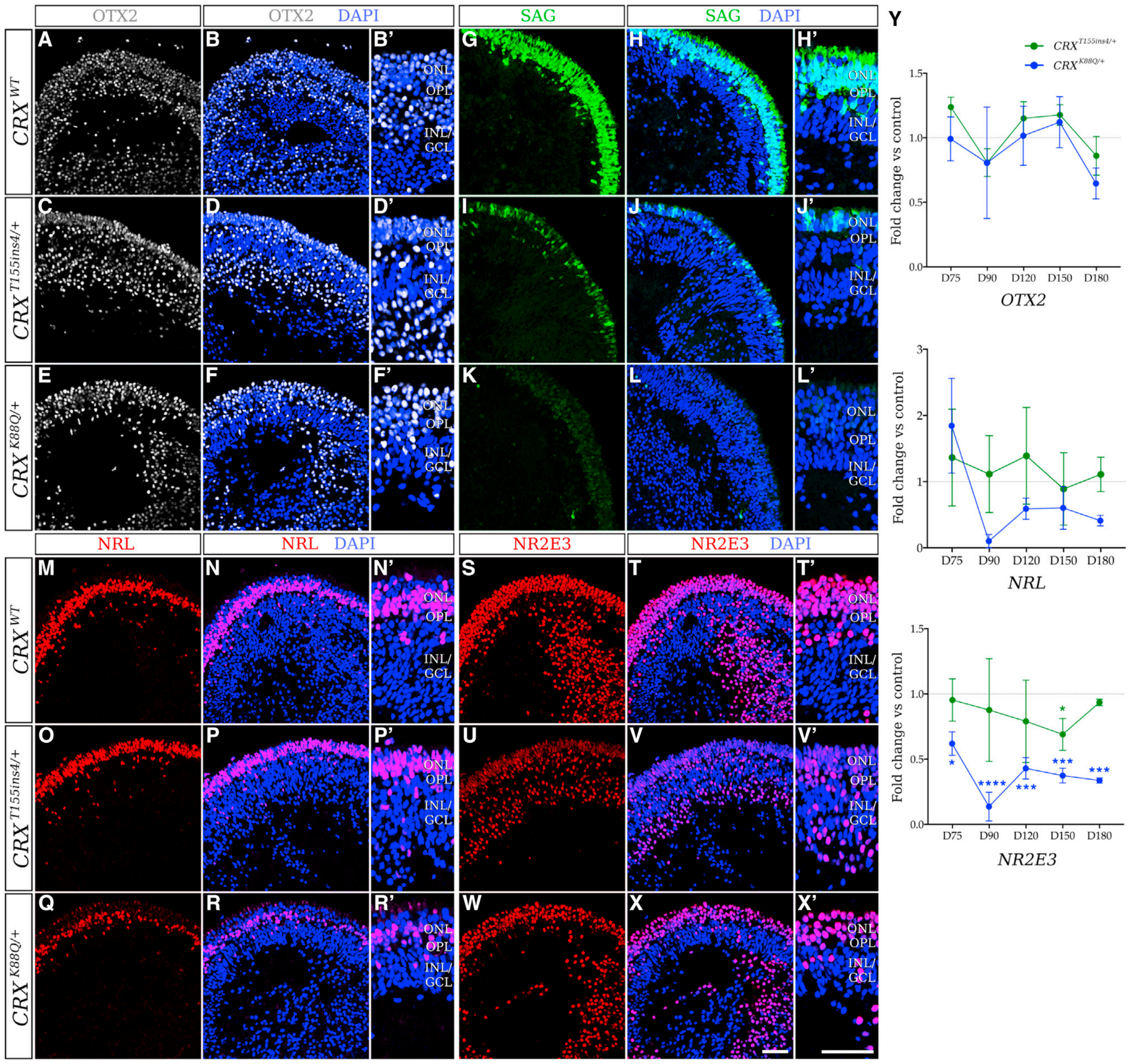

Figure 4. Altered expression of rod photoreceptor cell markers in LCA7 retinal organoids at D180

(A–Y) Immunofluorescence staining using antibodies against OTX2 (white; A–F′), SAG (green; G–L′), NRL (red; M–R′), and NR2E3 (red; S–X′) are shown for control (CRX^{WT}; A–B′, G–H′, M–N′, S–T′), $CRX^{T155ins4/+}$ (C–D′, I–J′, O–P′, U–V′), and $CRX^{K88Q/+}$ (E–F′, K–L′, Q–R′, W–X′) retinal organoids at D180 (n = 9 total organoids from three experimental replicates per line). Nuclei are counterstained with DAPI (blue). OPL, outer plexiform layer; INL/GCL, inner nuclear layer/ganglion cell layer. qRT-PCR data (Y) are shown for $CRX^{T155ins4/+}$ (green line) and $CRX^{K88Q/+}$ (blue line) at D75, D90, D120, D150, and D180 as fold change compared with control organoids for *OTX2*, *NRL*, and *NR2E3* (n = 14 total organoids from two experimental replicates per line). The dotted line represents no change compared with control (y = 1). All statistical analyses were performed using one-way ANOVA with a Dunnett test to correct for multiple comparisons in GraphPad Prism 8 software. Scale bar (X and X′), 100 μm. *$p < 0.05$, ***$p < 0.005$, ****$p < 0.001$.
See also Figures S3, S4, and S7.

mutations in *CRX* (Figure S5). Although clear photoreceptor defects can be observed in the LCA7 organoids, these cells form normal-looking ribbon synapses with unaltered expression of key ribbon synapse markers (Figures 2I–2K; Table S5). However, we did observe moderate changes in some synaptic vesicle markers, including SV2B, SNAP25, and

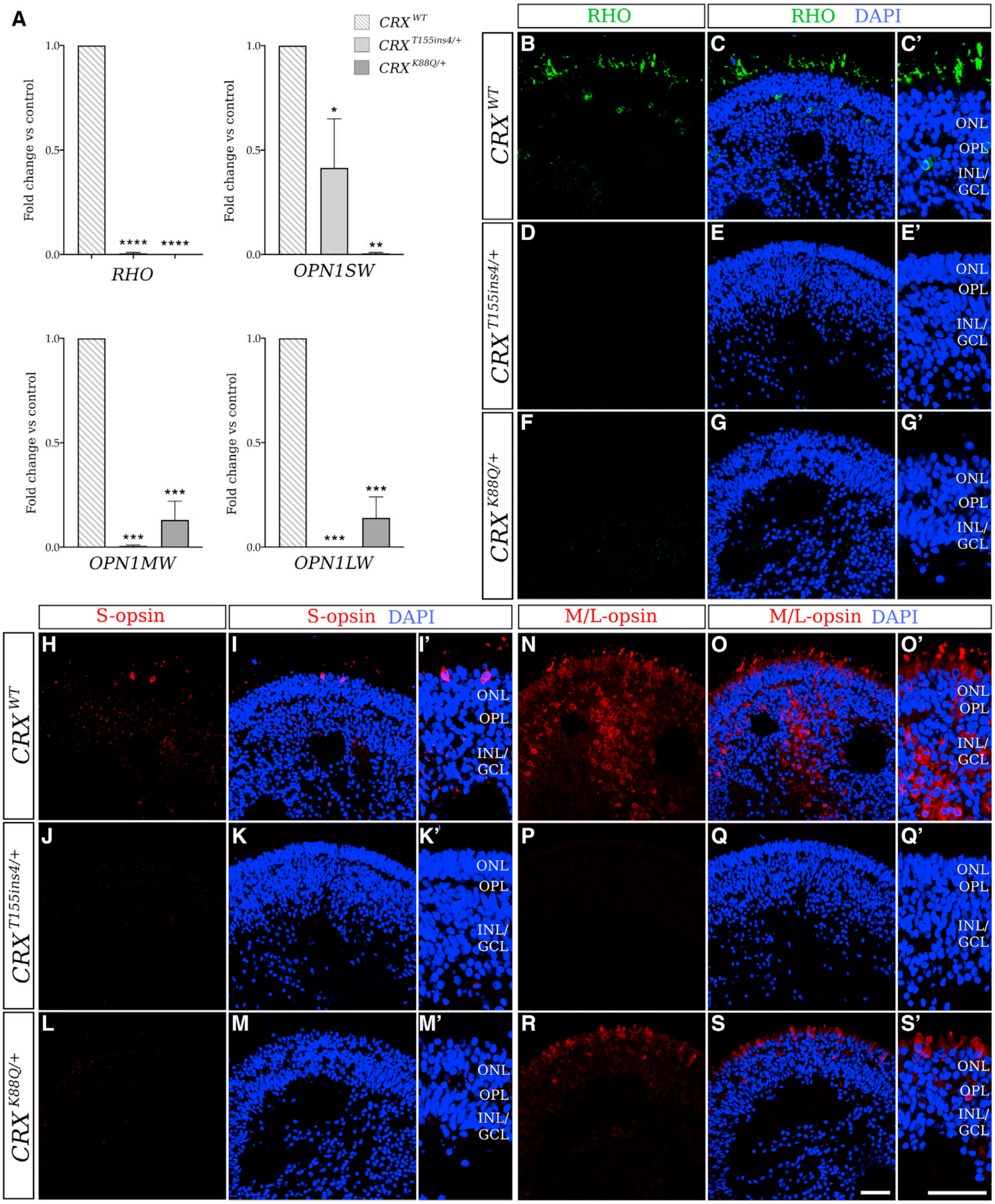

Figure 5. qRT-PCR and immunofluorescence staining reveal significant downregulation of late photoreceptor cell markers in LCA7 organoids at D180

(A–S′) qRT-PCR data (A) are shown for *CRX*^{WT} (gray lines), *CRX*$^{T155ins4/+}$ (light gray), and *CRX*$^{K88Q/+}$ (dark gray) at D180 as fold change compared with control organoids for *RHO*, *OPN1SW*, *OPN1MW*, and *OPN1LW* (n = 14 total organoids from two experimental replicates per line). $^{*}p < 0.05$, $^{**}p < 0.01$, $^{***}p < 0.005$, $^{****}p < 0.001$. Immunofluorescence staining using antibodies against RHO (green; B–G′), S-opsin (red; H–M′), and M/L-opsin (green; N–S′) is shown for control (*CRX*^{WT}; B–C′, H–I′, N–O′), *CRX*$^{T155ins4/+}$ (D–E′, J–K′, P–Q′), and *CRX*$^{K88Q/+}$ (F–G′, L–M′, R–S′) retinal organoids at D180 (n = 9 total organoids from three experimental replicates per line). Nuclei are counterstained with DAPI (blue). Scale bar (S and S′), 100 μm. OPL, outer plexiform layer; INL/GCL, inner nuclear layer/ganglion cell layer. All statistical analyses were performed using one-way ANOVA with a Dunnett test to correct for multiple comparisons in GraphPad Prism 8 software. See also Figures S3 and S4.

STX3 (Figure 2L–2Q and S5D). We also saw a significant decrease in cytoskeletal matrix of the active zone (CAZ) marker levels, including *RIMS2*, *MPP4*, and *UNC119*, in both LCA7 organoids (Figure S5D; Table S5). These data are supported by previously published work demonstrating the role of CRX in presynaptic active zone formation in mice (Assawachananont et al., 2018).

Single-cell transcriptome analysis reveals photoreceptor-specific defects in LCA7 retinal organoids

To comprehensively capture changes in retinal gene expression for the LCA7 organoids compared with control, we performed single-cell RNA sequencing (scRNA-seq) using the 10X Genomics platform at D150 of differentiation. Three-dimensional principal component analysis to assess global gene expression changes revealed overlapping clusters for the two LCA7 lines, which were equally separated from the control line when plotting the first two principal components (Figure S5A), suggesting similar divergence in gene expression patterns for the LCA7 lines. The overlapping LCA7 clusters separated into distinct clusters in the third principal component, indicating that differences in gene expression exist between the two lines (Figure S5B). To broadly compare effects of the gene mutations on retinal and photoreceptor development and maturation, we carried out gene set enrichment analysis (GSEA) on significantly over- and under-expressed genes in the datasets for each mutation (Table S6). The top GSEA categories point primarily to a defect in photoreceptor maturation, leading to impaired phototransduction and light perception (Figure 6). In alignment with our qRT-PCR data, the scRNA-seq data revealed significant dysregulation of specific photoreceptor genes between control and LCA7 organoids, as well as mutation-specific differences in various genes, including *CRX*, *RCVRN*, *ARR3*, and *AIPL1* (Figures 6 and S5, Table S5). These data also revealed upregulation of OTX2 in our LCA7 organoids at D150 (Figure S5), which was not observed through IF or qRT-PCR data analyses. This change in *OTX2* expression might indicate a compensatory response by the diseased photoreceptor cells, as OTX2 and CRX share a number of gene targets in photoreceptor cells (Samuel et al., 2014).

Allele-specific editing of mutant *CRX* allele in patient-derived hiPSCs rescues photoreceptor defects in LCA7 retinal organoids

Based on data published in *Crx*$^{+/-}$ mice (Furukawa et al., 1999), and a case study examining a human cohort with a *CRX* deletion mutation (Ibrahim et al., 2018), we know that one copy of CRX is sufficient to allow for proper maturation (although delayed) and function in photoreceptor cells. Therefore, we wanted to explore a therapeutic approach that would eliminate the mutant allele and allow the wild-type protein to be solely expressed. To this end, we took a dual-cutting approach using CRISPR/Cas9 tools. To achieve dual cutting, we first identified single nucleotide polymorphisms (SNPs) that were only present on the mutant allele. Using Sanger sequencing, we identified five SNPs on the mutant allele upstream of the *K88Q* mutation (data not shown), designed single guide RNA oligonucleotides (gRNAs) to target each SNP independently, and then cloned those guides into the PX459 plasmid containing wild-type Cas9 (Figure S7A). To eliminate the mutant *CRX* allele in *CRX*$^{K88Q/+}$ hiPSCs, we simultaneously introduced two of the PX459 plasmids, one containing a gRNA targeting the *K88Q* mutation, and one containing a gRNA targeting an upstream allelic SNP (Figure 7A). The most successful gRNA targeted an SNP located just downstream of exon 2, within the intron (Table S2). The edited allele could be identified as a 374-bp band in a PCR gel (Figure 7B), and Sanger sequencing confirmed the loss of the *K88Q* mutation (Figure 7C). The resulting hiPSC line will be referred to as *CRX*$^{+/-}$ going forward.

The *CRX*$^{+/-}$ hiPSCs were differentiated alongside control and *CRX*$^{K88Q/+}$ hiPSCs, and organoid morphology for the *CRX*$^{+/-}$ line appeared normal through D180 of differentiation. IF data at D90, D120, D150, and D180 showed a dramatic increase in SAG, RCVRN, and ARR3 levels in the *CRX*$^{+/-}$ organoids compared with *CRX*$^{K88Q/+}$ (Figures 7D–7U′ and S6). While we found RCVRN levels to be comparable between *CRX*$^{+/-}$ and control organoids at D180, the levels of SAG and ARR3 in the *CRX*$^{+/-}$ organoids do not fully reach those of the control organoids. This could suggest a mild delay in photoreceptor maturation, which would be in line with the developmental delay observed in *CRX*$^{+/-}$ mouse retinae (Furukawa et al., 1999).

DISCUSSION

In the current study, we set out to generate an *in vitro* model of LCA7, using 3D human retinal organoids. iPSC lines were made from LCA patients with dominant disease-causing variants in the *CRX* gene. Those hiPSC lines were then differentiated to generate retinal organoids with LCA7 mutations: *CRX*$^{T155ins4/+}$ or *CRX*$^{K88Q/+}$. We discovered a clear morphological phenotype in our LCA7 organoids by D180, at which time the organoids had not developed outer-segment-like projections, in contrast to control organoids. This phenotype persisted to D240, which was the latest time point we analyzed for this study. We also observed a photoreceptor cell-specific phenotype within the LCA7 organoids, with decreased expression of early pan-photoreceptor markers, including recoverin/RCVRN and AIPL1. Both LCA7 organoids exhibited lower levels of rod-specific markers,

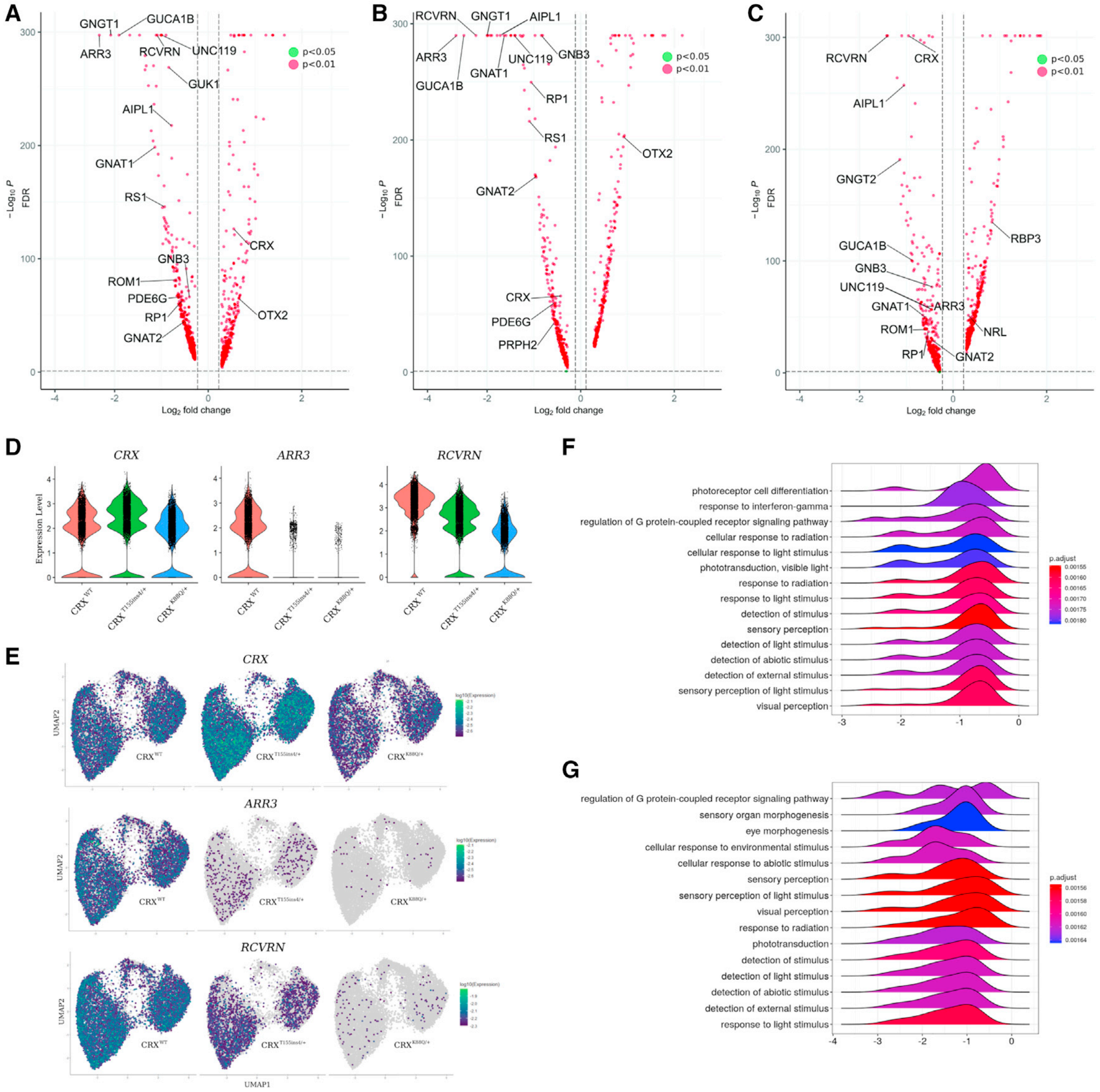

Figure 6. scRNA-seq of D150 reveals changes to photoreceptor transcripts in LCA7 versus control organoids

(A–G) Volcano plots comparing $CRX^{T155ins4/+}$ transcripts with those of control organoids (A), $CRX^{K88Q/+}$ transcripts to those of control (B), and $CRX^{K88Q/+}$ to $CRX^{T155ins4/+}$ transcripts (C). Green dots represent genes with a $\log_2$ fold change >0.2 and an adjusted p value of $p < 0.05$. Red dots represent genes with a $\log_2$ fold change >0.2 and an adjusted p value of <0.01. Expression levels for *CRX*, *ARR3*, and *RCVRN* are shown as violin plots (D) for control (CRX^{WT}, red), $CRX^{T155ins4/+}$ (green), and $CRX^{K88Q/+}$ (blue). UMAP graphs for *CRX*, *ARR3*, and *RCVRN* transcripts are also shown for all three genotypes (E). GSEA was performed for genes with significantly altered expression in $CRX^{T155ins4/+}$ (F) and $CRX^{K88Q/+}$ (G) organoids, and data for the top 15 categories are shown via ridge plots. The x axis represents fold change, and the y axis represents the number of genes. Libraries were prepared using n = 5 organoids per line. Statistical analyses were performed using the Wilcoxon rank-sum test and genes with $\log_2$ fold change of ≥ 0.25 and a p value <0.05 were considered significant.
See also Figure S5.

including rod arrestin/SAG and rhodopsin/RHO, and cone-specific markers, including cone arrestin/ARR3 and all three of the cone opsins. All of the markers analyzed in this study are critical for normal photoreceptor function, and downregulation of any one of them could lead to dysfunctional photoreceptor cells. Therefore, dysregulation of all of these key genes at the same time in the LCA7 organoids indicates severe photoreceptor disease. Together, these data suggest the presence of immature photoreceptor cells in the LCA7 retinal organoids, which aligns nicely with previously published work in LCA7 mice (Tran et al., 2014), and retinal organoids (Kruczek

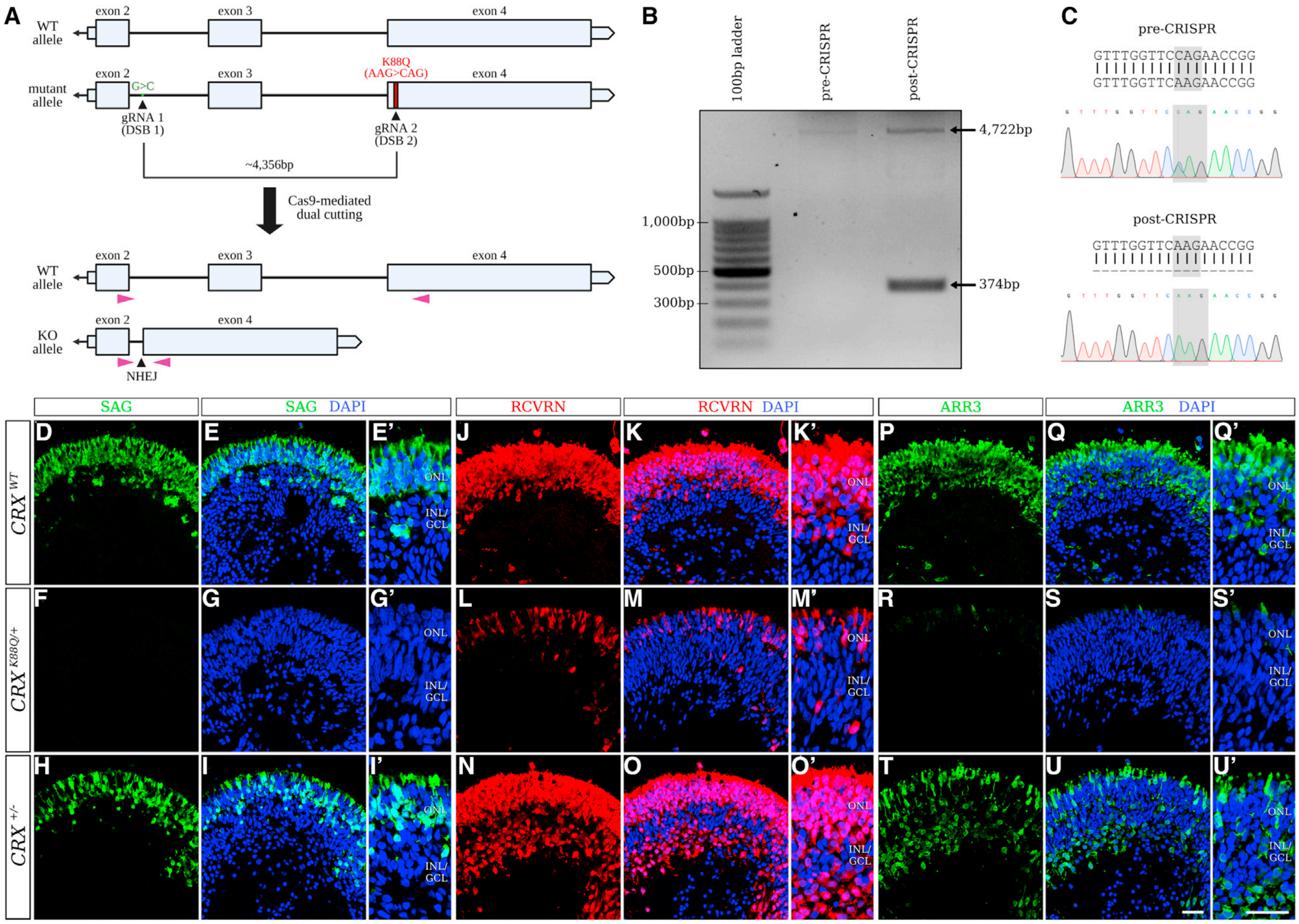

Figure 7. CRISPR/Cas9-mediated knockout of the mutant *CRX* allele in patient hiPSC

(A–C) The CRISPR/Cas9 dual-cutting target sites are mapped onto the mutant allele of the *CRX* gene (A). PCR was performed using primers shown in (A; purple triangles), revealing an additional 374-bp band representing the edited "knockout (KO) allele" after CRISPR/Cas9 editing (B). Sanger sequencing was also utilized to confirm loss of the K88Q mutation after CRISPR-mediated editing (C).
(D–U′) Immunofluorescence staining using antibodies against SAG (green; D–I′), RCVRN (red; J–O′), and ARR3 (green; P–U′) are shown for control (CRX^{WT}; D–E′, J–K′, and P–Q′), $CRX^{K88Q/+}$ (F–G′, L–M′, and R–S′), and $CRX^{+/-}$ (H–I′, N–O′, and T–U′) retinal organoids at D180 (n = 3 organoids per line). Nuclei are counterstained with DAPI (blue). Scale bars (U and U′), 100 μm. OPL, outer plexiform layer; INL/GCL, inner nuclear layer/ganglion cell layer.
See also Figures S6 and S7.

et al., 2021), providing a representative model system for this disease.

Furthermore, we saw distinct differences in the photoreceptor phenotype for the $CRX^{K88Q/+}$ organoids compared with the $CRX^{T155ins4/+}$ organoids, with the *K88Q* mutation resulting in a more significant decrease in mRNA and/or protein for some of the affected markers: CRX, RCVRN, AIPL1, SAG, and ARR3. We also observed downregulation of two key photoreceptor markers, NRL and NR2E3, that were unaffected by the *T155ins4* mutation at D180. In contrast, M-opsin/OPN1MW and L-opsin/OPN1LW were downregulated to a larger extent in the $CRX^{T155ins4/+}$ organoids compared with $CRX^{K88Q/+}$. Finally, the morphology differs slightly between the $CRX^{T155ins4/+}$ and $CRX^{K88Q/+}$ organoids, with the former having more severely stunted inner segment projections. Despite that, neither of the LCA7 lines form outer segments. Interestingly, one of the *T155ins4* patients exhibited slower visual decline (hand-motion vision and a measurable Goldmann visual field peripherally at age 12 years) compared with the patient with the *K88Q* mutation (bare light perception by the age of 4 years), aligning nicely with the severity of the photoreceptor phenotype in our retinal organoids. These differences may be attributed to the type and location of the mutations, with *K88Q* affecting the homeobox domain, and the *T155ins4* mutation causing a frameshift and early

stop, likely resulting in a truncated $CRX^{T155ins4}$ protein that lacks a complete transactivation domain. This early stop results in the formation of a truncated $CRX^{T155ins4}$ protein. The exact mechanism(s) for these variant-specific differences will need to be investigated further in future experiments, and could lend insight into the cause(s) for phenotypic variability observed in LCA7 patients. The mechanism(s) behind the variant-specific differences in *CRX* mRNA and protein levels is unknown. One hypothesis is that the variants alter the stability of the CRX mRNA molecules, with the T155ins4 mutation resulting in stabilized mRNA, and the K88Q mutation resulting in destabilized mRNA, similar to what has been hypothesized in mouse models (Tran et al., 2014). Another hypothesis is that there is a mutant protein-mediated reduction in overall CRX function, which subsequently drives the expression of both wild-type and T155ins4 *CRX* alleles in an attempt to compensate. The latter hypothesis, however, does not explain the observed decrease in the $CRX^{K88Q/+}$ organoids. Investigations into this intriguing phenomenon will be carried out in future studies.

A recent study generated a similar model, where they made retinal organoids from patient-derived hiPSCs harboring dominant mutations in *CRX* (Kruczek et al., 2021). Although the mutations studied by Kruczek and colleagues differ slightly from those shown here ($CRX^{I138fs48/+}$ and $CRX^{K88N/+}$), the resulting proteins likely act quite similarly to ours. In alignment with that hypothesis, our data for the $CRX^{T155ins4/+}$ organoids show similar trends to those of the $CRX^{I138fs48/+}$ organoids at corresponding time points for CRX, recoverin, rhodopsin, and L/M-opsin levels. Likewise, our $CRX^{K88Q/+}$ organoids have expression patterns much like those of the $CRX^{K88N/+}$ organoids. Despite these similarities, $CRX^{K88Q/+}$ organoids appear to have a more drastic decrease in RCVRN immunolabeling at both D180 and D240 compared with 200-day-old $CRX^{K88N/+}$ organoids, whereas there is less of a change in L/M-opsin immunolabeling in our organoids compared with theirs. Finally, in contrast to the $CRX^{I138fs48/+}$ organoids, we observed a significant downregulation of *OPN1SW* mRNA in the $CRX^{T155ins4/+}$ organoids compared with control.

Gene augmentation has been used successfully to treat other forms of LCA, like the RPE65-based voretigene neparvovec (Russell et al., 2017), and Kruczek and authors show promising evidence of rescue using gene augmentation to overexpress wild-type CRX in *CRX*-mutant retinal organoids using adeno-associated viruses (AAVs) (Kruczek et al., 2021). Here, we use an alternative approach that utilizes CRISPR/Cas9-based gene editing to selectively eliminate the mutant allele, allowing wild-type CRX to resume normal function unhindered. This approach was established because most LCA-causing mutations in *CRX* are dominant, and we know that CRX is largely haplosufficient (Furukawa et al., 1999; Ibrahim et al., 2018). The CRISPR/Cas9 system was used in this study due to its efficiency in generating targeted double-stranded DNA breaks at precise locations in the genome. For the initial proof-of-concept study presented here, the allele harboring the *K88Q* mutation in the $CRX^{K88Q/+}$ hiPSC line was targeted, along with an upstream SNP present on the mutant allele of *CRX*. These cells were then differentiated and were found to have a substantial increase in CRX, RCVRN, ARR3, and SAG compared with the unedited $CRX^{K88Q/+}$ organoids. These data suggest that the allele-specific editing approach is a promising strategy to provide at least moderate rescue of photoreceptor cell development and maturation in retinal organoids by D180. Going forward, we will test this strategy in developing organoids, for both the $CRX^{K88Q/+}$ and $CRX^{T155ins4/+}$ lines, to determine efficiency of targeting photoreceptor cells with these tools, and to establish a timeline for effective therapeutic intervention. This method could theoretically be applied to any dominant *CRX* mutation, and could work for additional dominant genetic diseases in other tissues, providing a broader application outside the specific mutations presented in this work.

EXPERIMENTAL PROCEDURES

PBMC collection and reprogramming

This study was approved by the Institutional Review Board (Induced Pluripotent Stem Cells for Retinal Research, IRB# 18-26,409) at the University of California San Francisco (UCSF). Patients were seen by a retinal specialist at one of the UCSF Department of Ophthalmology clinics, and were recruited into the study after informed consent. Relevant donor information can be found in Table S1, and the full sample processing and reprogramming details can be found in the supplemental experimental procedures.

CRX gene sequencing

To determine the presence or loss of *CRX* variants in the hiPSC lines, a 597-bp region of the *CRX* gene surrounding the variants was amplified using PCR with a Taq polymerase (Thermo Scientific, EP0402). The reactions were run through a 1% agarose gel, and the DNA was isolated and cleaned up using the QIAquick Gel Extraction Kit (Qiagen, 28,704). *CRX* amplicons were then sent to Eurofins Scientific for Sanger sequencing. This method was also used to identify non-disease-causing allelic SNPs, which could then be used as a target for Cas9-mediated disruption of the mutant *CRX* allele. See supplemental experimental procedures for more details.

Generating CRISPR-Cas9 plasmids and homology-directed repair donor constructs

All CRISPR-Cas9 single guide RNAs (gRNAs) used for this study were designed with the tools available through the Benchling informatics platform (www.benchling.com). gRNAs targeting

the dominant disease-causing *CRX* variant CRX^{K88Q} (c.262A>C), as well as gRNAs targeting allele-specific SNPs between exons 2 and 4 of the *CRX* gene were generated. Single gRNAs were cloned into the pSpCas9(BB)-2A-Puro (PX459) plasmid (Addgene, 62988) containing the *Streptococcus pyogenes Cas9* (*spCas9*) gene and the puromycin resistance (*PuroR*) gene for puromycin-mediated clonal selection. Oligo sequences can be found in Table S2.

Disruption of the mutant *CRX* allele in hiPSCs

The lipofectamine-based transfection protocol described above was also utilized to disrupt the mutant *CRX* allele in hiPSCs. To eliminate the mutant *CRX* allele, hiPSCs containing the $CRX^{K88Q/+}$ genotype were simultaneous transfected with two separate PX459-Cas9 plasmids (at a 1:1 ratio): one containing a gRNA directly targeting the *K88Q* mutation (3 μg), and one containing a gRNA targeting an SNP near exon 2 on the mutant allele (3 μg). After puromycin selection, individual clones were assessed by PCR amplification followed by Sanger sequencing of the *CRX* gene.

Retinal organoid differentiation

Retinal organoids were generated using a protocol derived from previously published work (Capowski et al., 2019; Ohlemacher et al., 2015; Zhong et al., 2014). Briefly, hiPSCs were grown on six-well tissue culture (TC)-treated plates coated with Matrigel (Corning, 354234), and fresh mTeSR Plus medium was given to the cells every other day. To passage cells, each well was rinsed with 1× Dulbecco's phosphate-buffered saline (DPBS) before applying EDTA (1:1,000 in 1× DPBS) to the cells for 2 min. EDTA was then aspirated and cells were given fresh mTeSR Plus medium before colonies were gently lifted using a cell lifter. Cells used to maintain the line were plated in a new Matrigel-coated six-well plate. Colonies used to make retinal organoids were carried through the differentiation protocol (Figure 1K). Phase images of the retinal organoids were captured using an Olympus IX51 inverted microscope. See supplemental experimental procedures for full details and media recipes.

Immunocytochemistry and immunofluorescence

Each newly reprogrammed hiPSC clone was grown on a 24-well TC-treated cell culture plate to reach ~50%–70% confluency. The cells were then fixed on the plate for 10 min at room temperature using 4% paraformaldehyde (PFA) prior to staining. Retinal organoids were collected at D75, D90, D120, D150, D180, and D240 (n = 3/clone per collection, with three separate collections per time point) for each genotype. Organoids were fixed for 30 min in 4% PFA. Cells and tissue sections were stained with various antibodies (see Table S3 for full list of antibodies) and counterstained with 4′,6-diamidino-2-phenylindole (DAPI, 1 μg/mL; Roche, 10236276001) for 2 min, before being imaged. Extended processing details are described in supplemental experimental procedures.

qRT-PCR analysis

Total RNA was extracted from six or seven retinal organoids per collection (n = 2 collections per clone) using the RNeasy Extraction Kit (Qiagen, 74104). cDNA was then generated using the iScript cDNA Synthesis Kit (Bio-Rad, 1708891). To run qRT-PCR experiments, the iTaq Universal SYBR Green Supermix (Bio-Rad, 1725124) was employed. Primer sequences can be found in Table S4. All data summary graphs were made using GraphPad Prism 8, and all statistical analyses were performed via one-way ANOVA with a Dunnett test to correct for multiple comparisons. See supplemental experimental procedures for more details.

TEM

D180 retinal organoids (hiPSC CRX^{WT} control, $CRX^{T155ins4/+}$, and $CRX^{K88Q/+}$) were fixed overnight in 4% PFA (Fisher Acros, 41678-500) + 2.5% glutaraldehyde (Polysciences, 00376) in 0.1 M PO_4 buffer solution and rinsed four or five times with 0.1 M PO_4 buffer. Fixed organoids were then stained in 1% osmium tetroxide (OsO_4; Electron Microscopy Sciences, 19180) in distilled H_2O (dH_2O) for 1 h at room temperature. Organoids were washed in five changes of cold dH_2O, followed by staining in 2% uranyl acetate (Electron Microscopy Sciences, 22400) in dH_2O for 1 h at 37°C. After washing in dH_2O, the organoids were dehydrated in ethanol (50%, 70%, 95%, 2 × 100%), for 20 min each. The dehydration process was continued with five 20-min incubations in absolute ethanol. The organoids were exposed to propylene oxide (PO; Polysciences Inc., 00235-1) for two 5-min rinses, and then infiltrated with a 1:1 ratio of Epon resin (Epon-812; Electron Microscopy Sciences, 13940) and PO overnight at room temperature. The next day, the mixture was replaced with degassed 100% Epon resin for 1–2 h. The organoids were then embedded in 100% Epon resin in PELCO Silicone Rubber Molds (Ted Pella Inc., PELCO 105) via polymerization for 48 h at 60°C. Sections 70 nm thick were collected on copper mesh grids and imaged using a Philips Tecnai 10 electron microscope.

scRNA-seq data analysis

Five retinal organoids per line (CRX^{WT}, $CRX^{T155ins4/+}$, and $CRX^{K88Q/+}$) were dissociated on D150 using the Papain Dissociation System (Worthington Biochemical Corp, LK003150) following manufacturer's instructions. Briefly, after applying pre-warmed papain solution (20 U/mL papain +0.0005% DNase) for 20 min at 37°C with gentle agitation, organoids were triturated using a sterile transfer pipette to generate a single-cell suspension. The cell suspension was centrifuged at 300 x g for 5 min to pellet the cells. After removing the supernatant, cells were resuspended in fresh 1:1 DMEM/F12 + 10% FBS and counted to determine cell concentration. Cells were then spun down once more at 300 × *g* for 5 min to pellet the cells. Cells were resuspended in 1× DPBS with 0.04% BSA to a final concentration of 1,300 cells/μL in 100 μL per line. Cells were partitioned and barcoded using a 10X Chromium Controller with a target recovery of 8,000 cells per line, followed by library construction with the Chromium Next GEM Single Cell 3′ Dual Index Reagent Kits v3.1 (10X Genomics, PN-1000269) according to the manufacturer's instructions. Single-cell libraries were sequenced on an Illumina NovaSeq at the UCSF Institute for Human Genetics Core.

The mean number of reads per cell for each line was 13,148 (CRX^{WT}), 8,525 ($CRX^{T155ins4/+}$), and 14,337 ($CRX^{K88Q/+}$), and the median unique molecular identifier (UMI) per cell was 926 (CRX^{WT}), 642 ($CRX^{T155ins4/+}$), and 868 ($CRX^{K88Q/+}$). CellRanger software (version 5.0.0) was used with default parameters for library demultiplexing, fastq file generation, read alignment, and UMI quantification. Further processing was carried out in Rstudio using Seurat

4.0.0 (Stuart et al., 2019). Seurat objects were crated to include genes expressed in at least 20 cells and cells with a minimum of 500 genes per cell. Data were further filtered to exclude cells with >2,500 genes or >20% mitochondrial genes. The datasets were merged and exported to Monocle 3 (Cao et al., 2019). Data were log normalized, and principal components were determined and scaled. Data from control (CRX^{WT}), $CRX^{K88Q/+}$, and $CRX^{T155ins4/+}$ organoids were aligned to remove batch effects using Batchelor in Monocle 3 (Haghverdi et al., 2018). To visualize nuclear transcriptomic profiles in two-dimensional space, Uniform Manifold Approximation and Projection (UMAP) was performed with the following parameters: umap.min_dist = 0.2, umap.n_neighbors = 10. Differential expression analysis was performed on normalized scaled data in Seurat using the Wilcoxon rank-sum test and genes with logfc.threshold of 0.25 and p value <0.05 were considered for volcano plots and enrichment analysis. Volcano plots were generated using the EnhancedVolcano package (www.bioconductor.org/packages/release/bioc/vignettes/EnhancedVolcano/inst/doc/EnhancedVolcano.html). GSEA was performed on differentially expressed genes using ClusterProfShinyGSEA from the NASQAR toolbox (Yousif et al., 2020).

Data and code availability

The datasets generated during this study are available on GEO: GSE184080.

SUPPLEMENTAL INFORMATION

Supplemental information can be found online at https://doi.org/10.1016/j.stemcr.2021.09.007.

AUTHOR CONTRIBUTIONS

K.R.C. planned experiments, conducted experiments, performed data analysis, and wrote the paper. S.C. conducted experiments and edited the paper. A.T.M. conducted experiments and edited the paper. J.L.D. conducted experiments, and wrote and edited the paper, D.A.L. planned experiments, performed data analysis, and wrote the paper.

CONFLICTS OF INTEREST

K.R.C., S.C., and D.A.L. declare no competing interests. A.T.M. has been a consultant for Roche, Nightstar, and 4-D Therapeutics, and has received funding to participate in clinical trials of gene therapy from AGTC Therapeutics. J.L.D. is a scientific advisory board member for Sparing Vision Inc, California Institute for Regenerative Medicine, and Vedere Bio; a consultant for Astellas, Biogen/Nightstarx Therapeutics, DTx Pharma, Editas Inc, Eloxx, Eyevensys, Gyroscope Therapeutics, and ProQR Therapeutics Inc; and is on the data safety monitoring board for clinical trials with AGTC Therapeutics and Spark Therapeutics. J.L.D. receives funding to support clinical trials from Allergan/Abbvie, Acucela, and Biogen/Nightstarx Therapeutics.

ACKNOWLEDGMENTS

We would like to thank the patients and their families for their generous blood donations, which were instrumental in this work. We also thank Yien-Ming Kuo at the UCSF Department of Ophthalmology Vision Core for TEM-related organoid processing and for microscopy support, Ivy Hsieh at the San Francisco VA Medical Center for her assistance with TEM, the Bioinformatics Team at the UCSF Institute for Human Genetics Core for scRNA-seq and data processing, and Dr. Bruce Conklin and members of his laboratory for the helpful discussions on genome editing. The research presented here is supported by the National Eye Institute (F32 EY031242 to K.R.C.; R01 EY032197 to D.A.L.; P30 Vision Core grant to UCSF Dept of Ophthalmology), the Research to Prevent Blindness (unrestricted grant to UCSF Dept of Ophthalmology), and a gift from the Claire Giannini Foundation to D.A.L. and J.L.D.

Received: April 18, 2021
Revised: September 14, 2021
Accepted: September 15, 2021
Published: October 14, 2021

REFERENCES

Artero Castro, A., Rodríguez Jimenez, F.J., Jendelova, P., and Erceg, S. (2019). Deciphering retinal diseases through the generation of three dimensional stem cell-derived organoids: Concise Review. Stem Cells *37*, 1496–1504. https://doi.org/10.1002/stem.3089.

Assawachananont, J., Kim, S.-Y., Kaya, K.D., Fariss, R., Roger, J.E., and Swaroop, A. (2018). Cone-rod homeobox CRX controls presynaptic active zone formation in photoreceptors of mammalian retina. Hum. Mol. Genet. *27*, 3555–3567. https://doi.org/10.1093/hmg/ddy272.

Cao, J., Spielmann, M., Qiu, X., Huang, X., Ibrahim, D.M., Hill, A.J., Zhang, F., Mundlos, S., Christiansen, L., Steemers, F.J., et al. (2019). The single-cell transcriptional landscape of mammalian organogenesis. Nature *566*, 496–502. https://doi.org/10.1038/s41586-019-0969-x.

Capowski, E.E., Samimi, K., Mayerl, S.J., Phillips, M.J., Pinilla, I., Howden, S.E., Saha, J., Jansen, A.D., Edwards, K.L., Jager, L.D., et al. (2019). Reproducibility and staging of 3D human retinal organoids across multiple pluripotent stem cell lines. Development *146*. https://doi.org/10.1242/dev.171686.

Corbo, J.C., Lawrence, K.A., Karlstetter, M., Myers, C.A., Abdelaziz, M., Dirkes, W., Weigelt, K., Seifert, M., Benes, V., Fritsche, L.G., et al. (2010). CRX ChIP-seq reveals the cis-regulatory architecture of mouse photoreceptors. Genome Res. *20*, 1512–1525. https://doi.org/10.1101/gr.109405.110.

Furukawa, T., Morrow, E.M., Li, T., Davis, F.C., and Cepko, C.L. (1999). Retinopathy and attenuated circadian entrainment in Crx-deficient mice. Nat. Genet. *23*, 466–470. https://doi.org/10.1038/70591.

Haghverdi, L., Lun, A.T.L., Morgan, M.D., and Marioni, J.C. (2018). Batch effects in single-cell RNA-sequencing data are corrected by matching mutual nearest neighbors. Nat. Biotechnol. *36*, 421–427. https://doi.org/10.1038/nbt.4091.

Hennig, A.K., Peng, G.-H., and Chen, S. (2008). Regulation of photoreceptor gene expression by Crx-associated transcription factor network. Brain Res. *1192*, 114–133. https://doi.org/10.1016/j.brainres.2007.06.036.

den Hollander, A.I., Roepman, R., Koenekoop, R.K., and Cremers, F.P.M. (2008). Leber congenital amaurosis: genes, proteins and disease mechanisms. Prog. Retin. Eye Res. *27*, 391–419. https://doi.org/10.1016/j.preteyeres.2008.05.003.

Hull, S., Arno, G., Plagnol, V., Chamney, S., Russell-Eggitt, I., Thompson, D., Ramsden, S.C., Black, G.C.M., Robson, A., Holder, G.E., et al. (2014). The phenotypic variability of retinal dystrophies associated with mutations in CRX, with report of a novel macular dystrophy phenotype. Invest. Ophthalmol. Vis. Sci. *55*, 6934–6944. https://doi.org/10.1167/iovs.14-14715.

Ibrahim, M.T., Alarcon-Martinez, T., Lopez, I., Fajardo, N., Chiang, J., and Koenekoop, R.K. (2018). A complete, homozygous CRX deletion causing nullizygosity is a new genetic mechanism for Leber congenital amaurosis. Sci. Rep. *8*, 5034. https://doi.org/10.1038/s41598-018-22704-z.

Koenekoop, R.K. (2004). An overview of Leber congenital amaurosis: a model to understand human retinal development. Surv. Ophthalmol. *49*, 379–398. https://doi.org/10.1016/j.survophthal.2004.04.003.

Kruczek, K., and Swaroop, A. (2020). Pluripotent stem cell-derived retinal organoids for disease modeling and development of therapies. Stem Cells *38*, 1206–1215. https://doi.org/10.1002/stem.3239.

Kruczek, K., Qu, Z., Gentry, J., Fadl, B.R., Gieser, L., Hiriyanna, S., Batz, Z., Samant, M., Samanta, A., Chu, C.J., et al. (2021). Gene therapy of dominant CRX-Leber congenital amaurosis using patient stem cell-derived retinal organoids. Stem Cell Rep. *16*, 252–263. https://doi.org/10.1016/j.stemcr.2020.12.018.

Kumaran, N., Moore, A.T., Weleber, R.G., and Michaelides, M. (2017). Leber congenital amaurosis/early-onset severe retinal dystrophy: clinical features, molecular genetics and therapeutic interventions. Br. J. Ophthalmol. *101*, 1147–1154. https://doi.org/10.1136/bjophthalmol-2016-309975.

Ohlemacher, S.K., Iglesias, C.L., Sridhar, A., Gamm, D.M., and Meyer, J.S. (2015). Generation of highly enriched populations of optic vesicle-like retinal cells from human pluripotent stem cells. Curr. Protoc. Stem Cell Biol. *32*, 1H.8.1–1H.8.20. https://doi.org/10.1002/9780470151808.sc01h08s32.

Russell, S., Bennett, J., Wellman, J.A., Chung, D.C., Yu, Z.-F., Tillman, A., Wittes, J., Pappas, J., Elci, O., McCague, S., et al. (2017). Efficacy and safety of voretigene neparvovec (AAV2-hRPE65v2) in patients with RPE65-mediated inherited retinal dystrophy: a randomised, controlled, open-label, phase 3 trial. Lancet *390*, 849–860. https://doi.org/10.1016/S0140-6736(17)31868-8.

Samuel, A., Housset, M., Fant, B., and Lamonerie, T. (2014). Otx2 ChIP-seq reveals unique and redundant functions in the mature mouse retina. PLoS One *9*, e89110. https://doi.org/10.1371/journal.pone.0089110.

Stone, E.M. (2007). Leber congenital amaurosis–A model for efficient genetic testing of heterogeneous disorders: LXIV Edward Jackson Memorial Lecture. Am. J. Ophthalmol. *144*, 791–811.e6. https://doi.org/10.1016/j.ajo.2007.08.022.

Stuart, T., Butler, A., Hoffman, P., Hafemeister, C., Papalexi, E., Mauck, W.M., Hao, Y., Stoeckius, M., Smibert, P., and Satija, R. (2019). Comprehensive integration of single-cell data. Cell *177*, 1888–1902.e21. https://doi.org/10.1016/j.cell.2019.05.031.

Thompson, J.A., De Roach, J.N., McLaren, T.L., and Lamey, T.M. (2018). A mini-review: Leber congenital amaurosis: identification of disease-causing variants and personalised therapies. Adv. Exp. Med. Biol. *1074*, 265–271. https://doi.org/10.1007/978-3-319-75402-4_32.

Tran, N.M., Zhang, A., Zhang, X., Huecker, J.B., Hennig, A.K., and Chen, S. (2014). Mechanistically distinct mouse models for CRX-associated retinopathy. PLoS Genet. *10*, e1004111. https://doi.org/10.1371/journal.pgen.1004111.

Yousif, A., Drou, N., Rowe, J., Khalfan, M., and Gunsalus, K.C. (2020). NASQAR: a web-based platform for high-throughput sequencing data analysis and visualization. BMC Bioinformatics *21*, 267. https://doi.org/10.1186/s12859-020-03577-4.

Zhong, X., Gutierrez, C., Xue, T., Hampton, C., Vergara, M.N., Cao, L.-H., Peters, A., Park, T.S., Zambidis, E.T., Meyer, J.S., et al. (2014). Generation of three-dimensional retinal tissue with functional photoreceptors from human iPSCs. Nat. Commun. *5*, 4047. https://doi.org/10.1038/ncomms5047.

Stem Cell Reports, Volume *16*

Supplemental Information

Allele-specific gene editing to rescue dominant *CRX*-associated LCA7 phenotypes in a retinal organoid model

Kathleen R. Chirco, Shereen Chew, Anthony T. Moore, Jacque L. Duncan, and Deepak A. Lamba

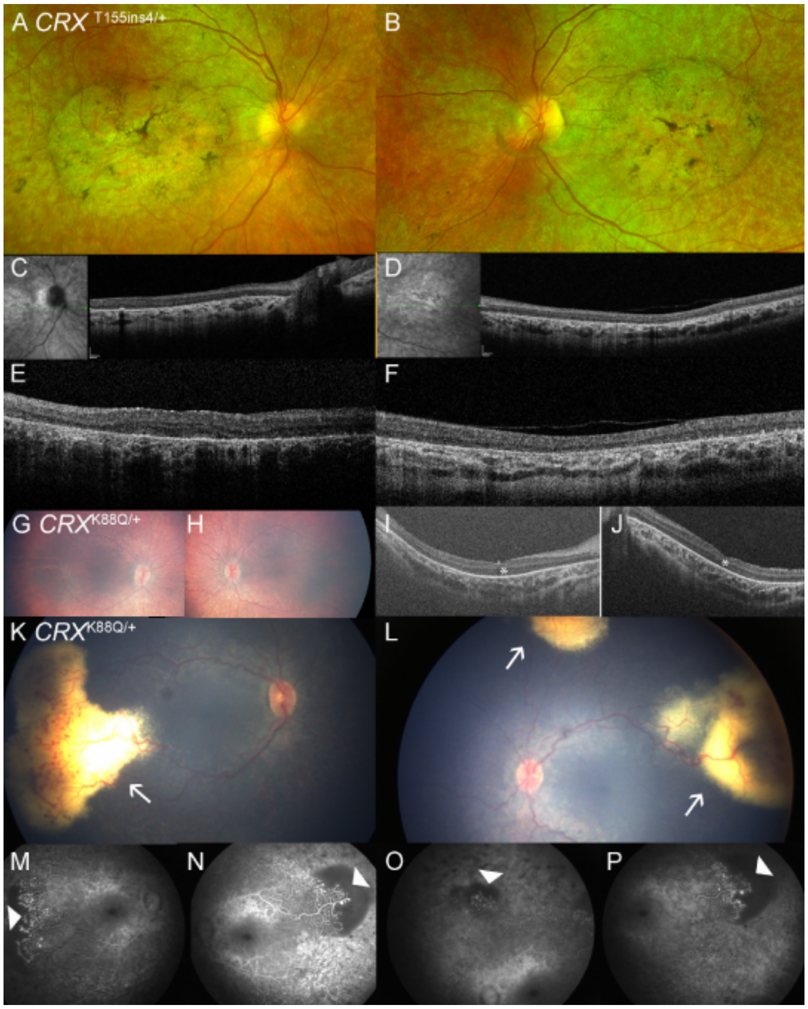

Figure S1. Retinal phenotype of two patients with CRX-associated LCA.
Color fundus photos of the right (**A**) and left (**B**) eyes of a 20-year-old with LCA (*CRX*$^{T155ins4/+}$) show bilateral macular atrophy with pigment clumping, mild disc pallor, retinal vascular attenuation, and diffuse retinal pigment epithelial (RPE) mottling outside the macular atrophy. Infrared fundus images and optical coherence tomography (OCT) scans through the macula of the right (**C**) and left (**D**) eyes reveal severe loss of the outer retinal layers in the macula magnified in panels (**E**) and (**F**), respectively; the posterior hyaloid is partially detached in the macula of the left eye (**D** and **F**). Color fundus photos of the right (**G**) and left (**H**) eyes of a 1 year old with LCA (*CRX*$^{K88Q/+}$) show diffuse RPE mottling and mild retinal vascular attenuation with a small region of preserved outer nuclear layer band centrally (*) visible in macular OCT scans from the right (**I**) and left (**J**) eyes. Color fundus photos at age 5 years show peripheral retinal vascular telangiectasias with lipid exudate temporally (arrows) in the right eye (**K**) and temporally and superiorly in the left eye (**L**). Early frames of fluorescein angiography at 1 minute and 32 seconds after injection of fluorescein dye in the right eye (**M**) and 25 seconds in the left eye (**N**) eyes show retinal vascular telangiectasias with peripheral nonperfusion (arrowheads) that stain in the mid-transit phase, 1 minute and 8 seconds (left eye, panel **O**) and 2 minutes and 37 seconds (left eye, panel **P**).

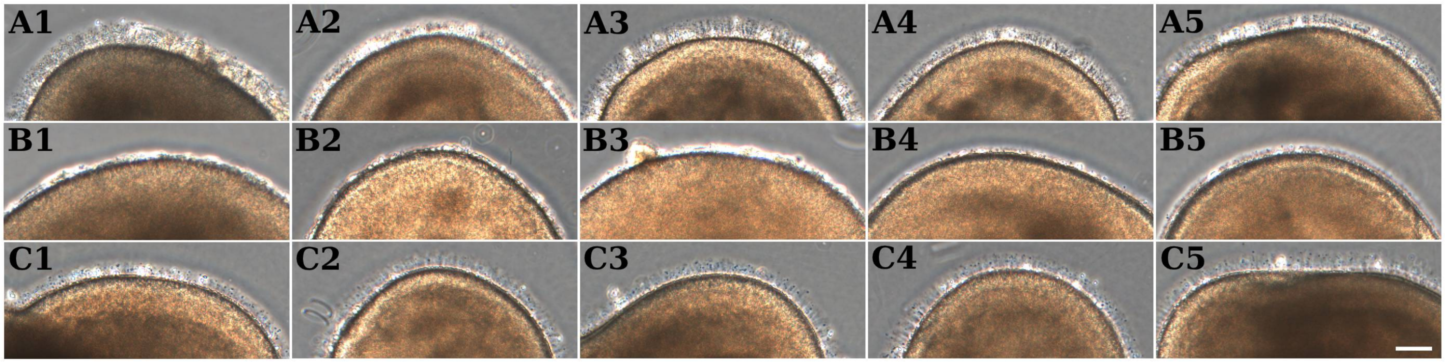

Figure S2. Consistency in early outer segment morphology across multiple differentiation experiments, *related to Figure 2*. Phase-contrast images for *CRX*^{WT} (**A1-A5**), *CRX*$^{T155ins4/+}$ (**B1-B5**), and *CRX*$^{K88Q/+}$ (**C1-C5**) retinal organoids at D180 were taken along the edge to show early outer segment morphological variability between five replicate differentiation experiments per line. Scale bar = 100µm.

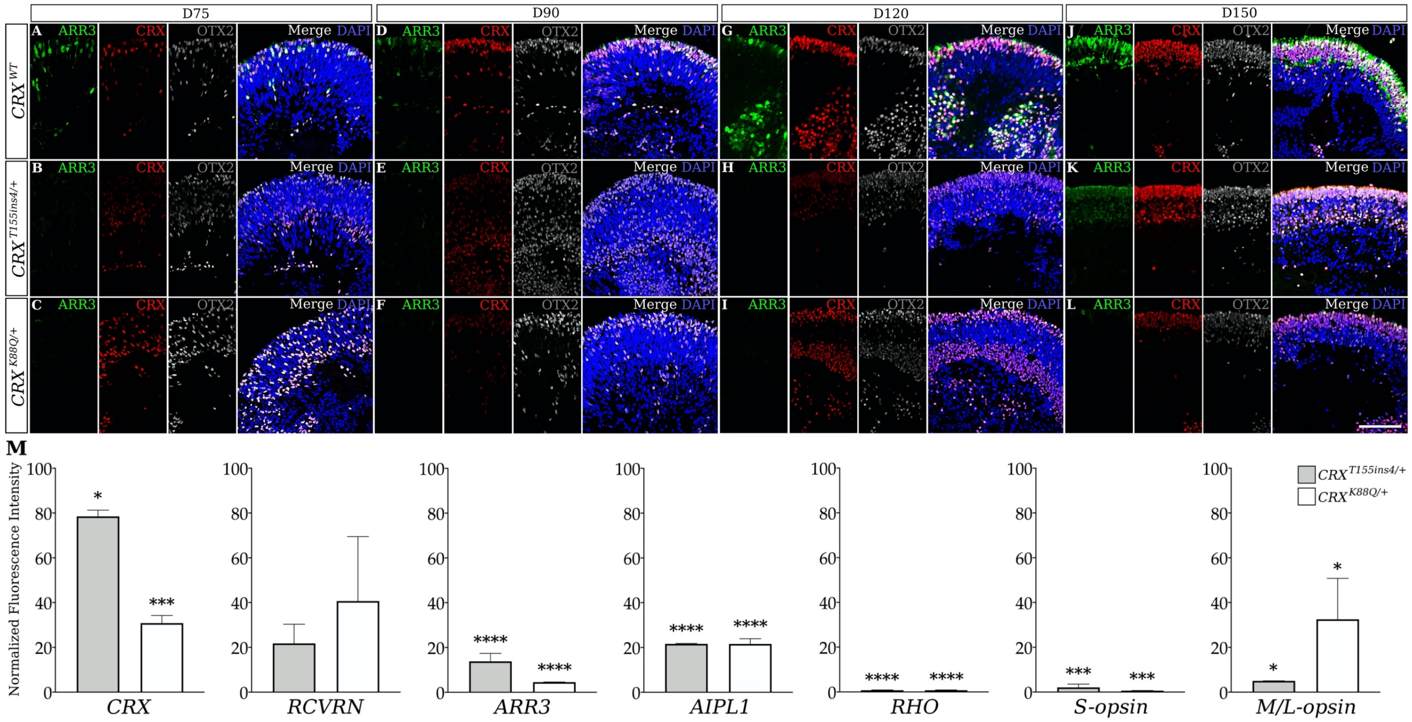

Figure S3. Immunofluorescence staining in retinal organoids at D75, D90, D120, and D150, and fluorescence pseudo-quantification in retinal organoids at D180, *related to Figure 3, Figure 4, and Figure 5*.

Antibodies against ARR3 (green), CRX (red), and OTX2 (white) are shown for *CRX*^{WT} (**A**, **D**, **G**, **J**), *CRX*$^{T155ins4/+}$ (**B**, **E**, **H**, **K**), and *CRX*$^{K88Q/+}$ (**C**, **F**, **I**, **L**) retinal organoids at D75 (**A-C**), D90 (**D-F**), D120 (**G-I**), and D150 (**J-L**). Nuclei are counterstained with DAPI (blue). Scale bar = 100μm. Fluorescence intensity (**M**) was quantified for CRX, RCVRN, ARR3, AIPL1, RHO, S-opsin, and M/L-opsin immunolabeling at D180 for *CRX*$^{T155ins4/+}$ (gray bar) and *CRX*$^{K88Q/+}$ (white bar) organoids. Fluorescence intensities were normalized to those of control organoids (n=3 organoids per marker per line).
* = p<0.05, *** = p<0.005, **** = p<0.001. All statistical analyses were performed using one-way ANOVA with a Dunnett test to correct for multiple comparisons in GraphPad Prism 8 software.

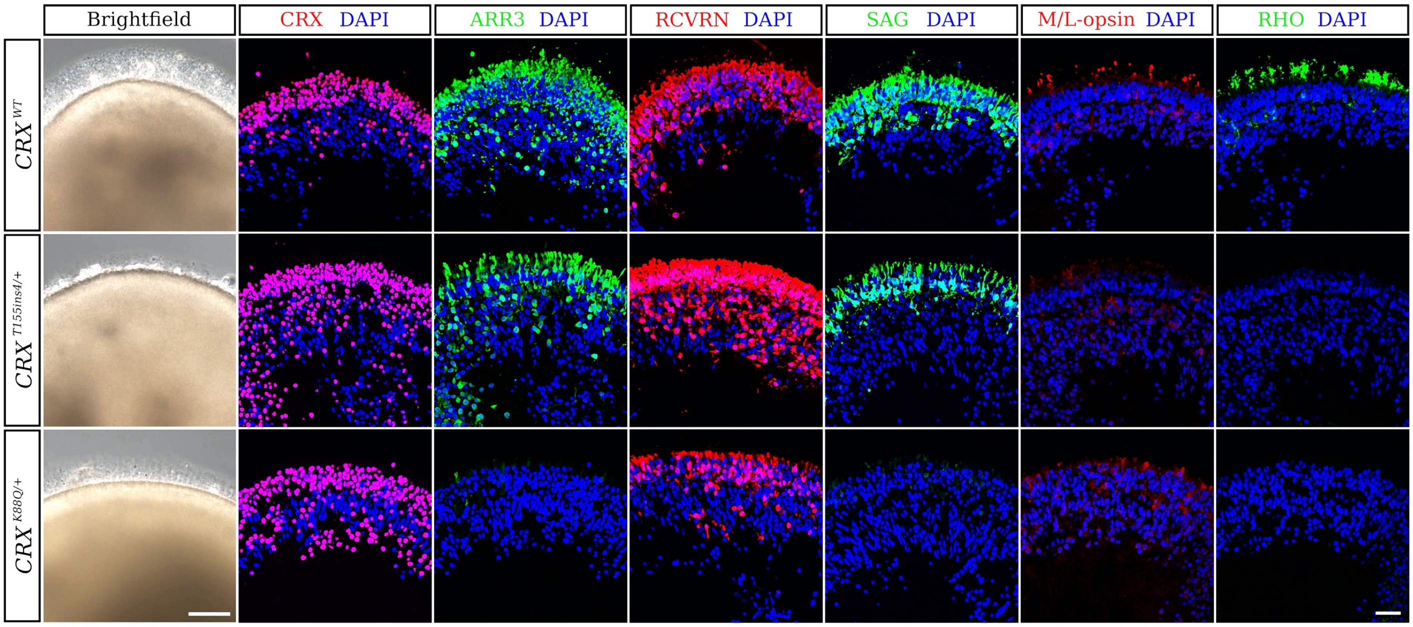

Figure S4. LCA7 retinal organoids show morphological defects and changes to photoreceptor cell markers at D240, *related to Figure 3, Figure 4, and Figure 5.*

Outer segment morphology is also shown in phase-contrast images of D240 retinal organoids for CRX^{WT}, $CRX^{T155ins4/+}$, and $CRX^{K88Q/+}$. IF staining using antibodies against CRX (red), ARR3 (green), RCVRN (red), SAG (green), M/L-opsin (red), RHO (green) are shown for all three genotypes. Nuclei are counterstained with DAPI (blue). Scale bars = 100μm.

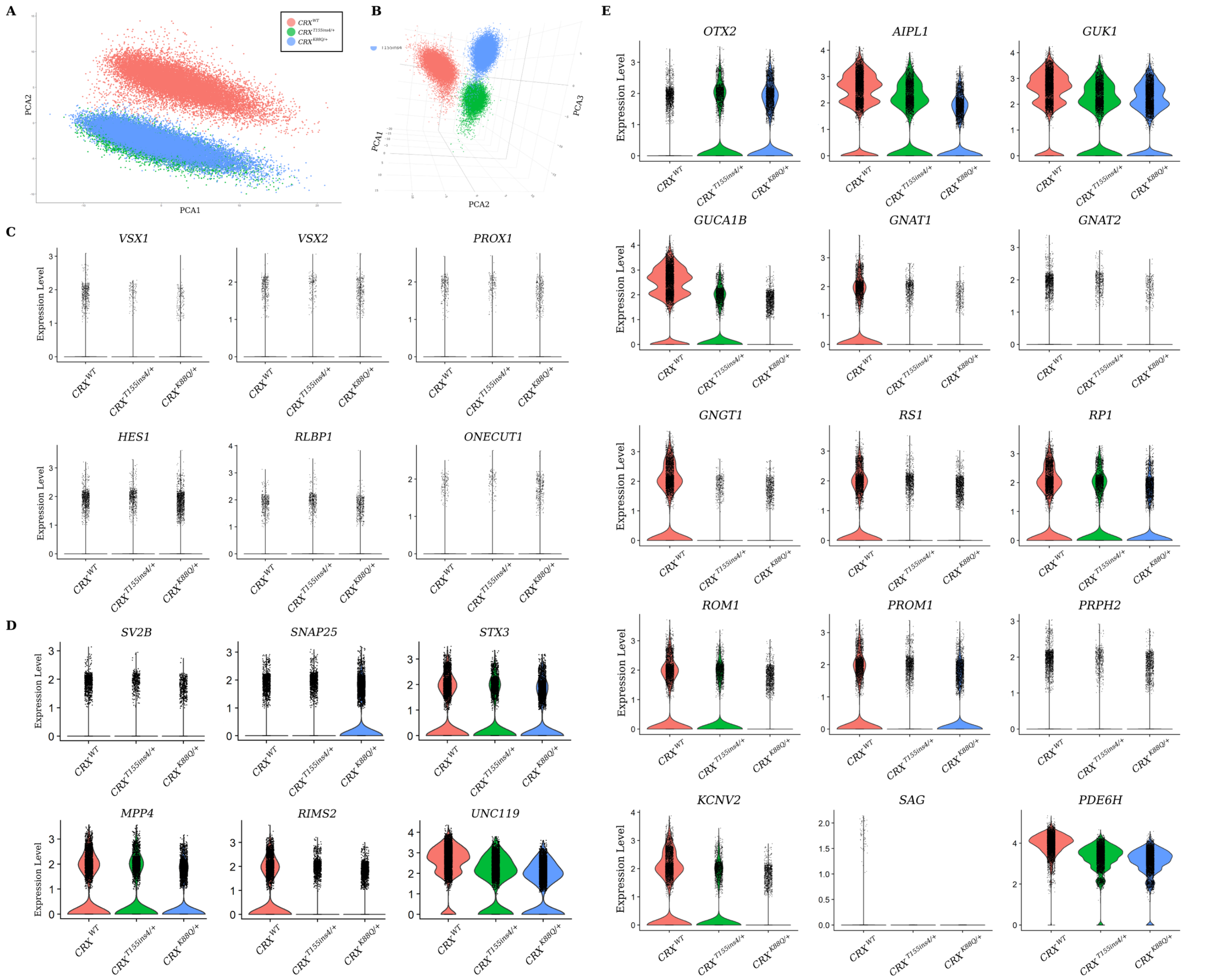

Figure S5. Single-cell RNA sequencing of D150 reveals distinct clusters between control and LCA7 organoid samples but little to no differences in non-photoreceptor cell markers, *related to Figure 2 and Figure 6*.

2D (**A**) and 3D (**B**) PCA plots show the distinct clusters for control (CRX^{WT}, red), $CRX^{T155ins4/+}$ (green), and $CRX^{K88Q/+}$ (blue) samples. Expression levels for bipolar cell (*VSX1*, *VSX2*), amacrine cell (*PROX1*), retinal progenitor cell/Müller glia (*HES1*, *RLBP1*), and retinal ganglion/horizontal cell (*ONECUT1*) transcript are shown for CRX^{WT} (red), $CRX^{T155ins4/+}$ (green), and $CRX^{K88Q/+}$ (blue) using violin plots (**C**). Expression data for vesicle markers (*SV2B*, *SNAP25, STX3*) and CAZ markers (*MPP4*, RIMS2, *UNC119*) are shown as violin plots (**D**) for CRX^{WT} (red), $CRX^{T155ins4/+}$ (green), and $CRX^{K88Q/+}$ (blue). Photoreceptor cell-specific gene expression levels are compared for CRX^{WT} (red), $CRX^{T155ins4/+}$ (green), and $CRX^{K88Q/+}$ (blue) using violin plots (**E**).

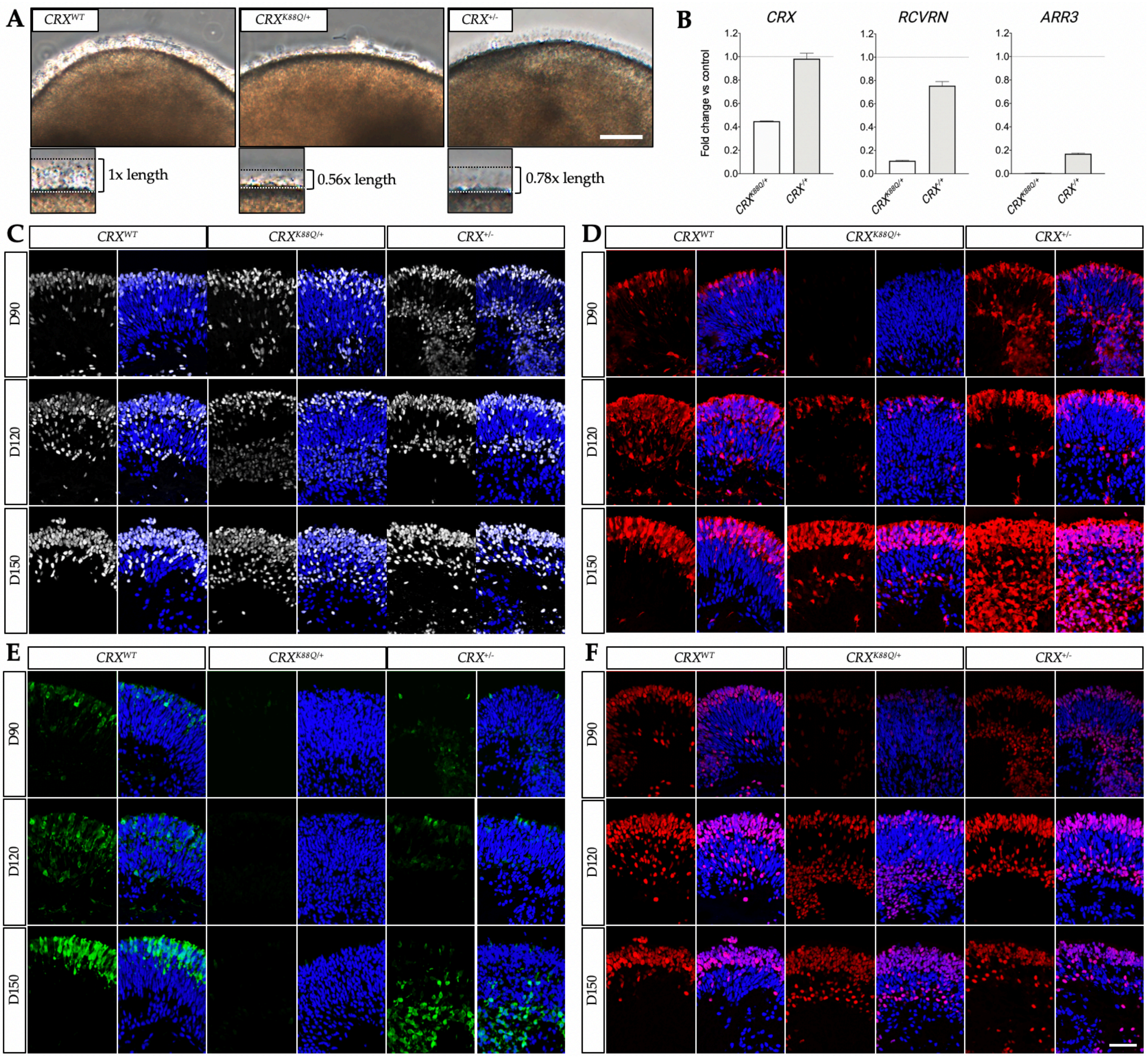

Figure S6. Analyses of $CRX^{+/-}$ retinal organoids at D90, D120, D150, and D180, *related to Figure 7*.
Outer segment length measurements for $CRX^{+/+}$, $CRX^{K88Q/+}$, and $CRX^{+/-}$ retinal organoids at D180 are shown in panel **A**. Preliminary qPCR data (n=6 organoids per sample from a single batch; statistics were performed on technical replicates) is shown at D180 for $CRX^{+/+}$, $CRX^{K88Q/+}$, and $CRX^{+/-}$ retinal organoids (**B**). Immunofluorescence staining with antibodies against OTX2 (white, **C**), RCVRN (red, **D**), ARR3 (green, **E**), and CRX (red, **F**) are shown for $CRX^{+/+}$, $CRX^{K88Q/+}$, and $CRX^{+/-}$ retinal organoids at D90, D120, and D150. Nuclei were counterstained with DAPI (blue, **C-F**). Scale bars (**A**&**F**) = 100µm.

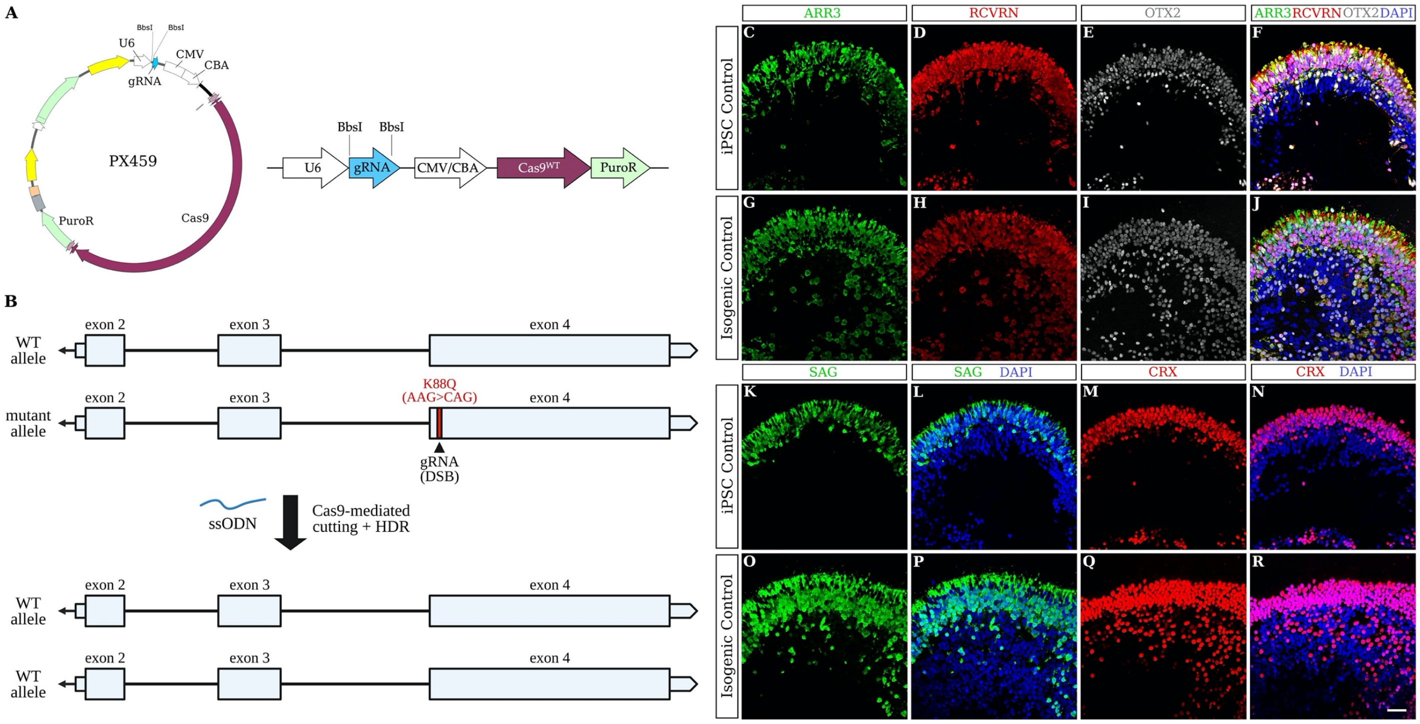

Figure S7. Generation of isogenic control hiPSC line and immunofluorescence in isogenic versus hiPSC control organoids at D180 of differentiation, *related to Figure 3, Figure 4, and Figure 7*.

To ensure our control hiPSC line is relevant in these studies, an isogenic control line was made for the *CRX*$^{K88Q/+}$ hiPSCs using CRISPR/Cas9 tools. A circular and linearized map of the PX459 plasmid is shown in **A**. The CRISPR/Cas9 target site is mapped onto the mutant allele of the *CRX* gene at the *K88Q* mutation (**B**). Immunofluorescence staining using antibodies against ARR3 (green; **C**, **F**, **G**, **J**), RCVRN (red; **D**, **F**, **H**, **J**), OTX2 (white; **E**, **F**, **I**, **J**), SAG (green; **K**, **L**, **O**, **P**), and CRX (red; **M**, **N**, **Q**, **R**) are shown for the hiPSC control (**C-F**, **K-N**) and the isogenic control *(***G-J**, **O-R**) retinal organoids at D180. Nuclei are counterstained with DAPI (blue). Scale bar (**R**) = 100µm.

Table S1. Patient donor information.

Sex	Ocular History	Visual Acuity	Age at time of blood collection	Variant
Female	LCA (nystagmus at birth); keratoconus with corneal scarring, cataracts	Bare light perception	44 y	*CRX*^{T155ins4}
Female	LCA (nystagmus at birth, retinal degeneration at 4mo)	Bare light perception	20 y	*CRX*^{T155ins4}
Female	LCA (nystagmus at 2 mo, severe retinal degeneration by 1yr)	Bare light perception	3 y	*CRX*^{K88Q}

Table S2. Oligonucleotide (oligo) sequences.

Name	Oligo sequence [PAM sequence]	Oligo size (bp)
gRNA K88Q	TGCATTTAGCCCTCCGGTTC [TGG]	20
gRNA SNP 1	GAGCCATTGTGCCTAGGCCC [GGG]	20
CRX^{K88Q} ssODN HDR construct	GCTCTCCTGGGCCTCTTCCCCACTTACCCACCCCCATCTCCGCT CTTATCCCCCAGGTTTGGTTCAAGAACCGGAGGGCTAAATGCAG GCAGCAGCGACAGCAGCAGAAACAGCAGCAGCAGCC	128

Table S3. Antibodies for immunofluorescence staining.

Target	Species	Manufacturer/Vendor	Catalog #	Dilution
AIPL1	Rabbit	Gift from Ramamurthy Lab, WVU	n/a	1:150
Blue cone opsin	Rabbit	Millipore	AB5407	1:50
Calbindin	Rabbit	Chemicon/Millipore	AB1778	1:200
CHX10/VSX2	Mouse	SCBT	sc-365519	1:50
Cone arrestin (7G6)	Mouse	Gift from Peter MacLeish Lab	n/a	1:100
CRX	Mouse	SCBT	sc-377138	1:50
CRX	Rabbit	Abcam	ab140603	1:100
Green/red cone opsin	Rabbit	Gift from Jeremy Nathans Lab	n/a	1:100
GFAP	Goat	Abcam	ab53554	1:500
Glutamine synthetase	Rabbit	Abcam	ab49873	1:2500
ISLET1/2	Mouse	DSHB	39.4D5	1:25
NANOG (lot 8)	Rabbit	Cell Signaling	4903	1:250
NRL	Goat	R&D	AF2945	1:100
OCT3/4	Mouse	SCBT	sc-5279	1:100
OTX2	Goat/Biotin	R&D	BAF1979	1:250
PKCa	Rabbit	Cell Signaling	2056T	1:200
PNR/NR2E3	Mouse	R&D	PP-H7223-00	1:25
Recoverin	Rabbit	Millipore	AB5585	1:600
Rhodopsin	Mouse	Millipore	MABN15MI	1:25
SOX2	Goat	R&D	AF2018	1:100
SV2	Mouse	DSHB	SV2	1:100
VGLUT1	Guinea Pig	Chemicon/Millipore	AB5905	1:250
Rod arrestin/SAG	Mouse	SCBT	sc-166383	1:50
Anti-mouse (AlexaFluor 488)	Donkey	Life Technologies	A-21202	1:200
Anti-rabbit (AlexaFluor 555)	Donkey	Life Technologies	A-31572	1:200
Anti-guinea pig (AlexaFluor 594)	Goat	Life Technologies	A-11076	1:200
Anti-goat (AlexaFluor 647)	Donkey	Life Technologies	A-21447	1:200

Table S4. qPCR and RT-PCR Primers.

Target mRNA	Forward primer (5'→3')	Reverse primer (5'→3')	Amplicon size (bp)
ACTB	GGATCAGCAAGCAGGAGTAT	GGTGTAACGCAACTAAGTCATAG	90
AIPL1	GGATCCCGAGTGATCTTTCATT	CAGACCTCGAGCTTGAACAT	128
ARR3	CAGGAATTCACGGAGACTGTAG	GGCCAGGTTGGTATCTTCAT	138
BCL2	GGATGCCTTTGTGGAACTG	CAGCCAGGAGAAATCAAACAG	61
CASP3	CTTCTTTAGAAACATCACGCATCAA	ACCTCAGGGAAACATTCAGAAA	102
CRX	CTGTTTGCCAAGACCCAGTA	CAAACCTGAACCCTGGACT	89
NR2E3	TGATGTCACCAGCAATGACC	TCTTCCAGCAGGATCACCT	180
NRL	GCCCAGTCCCTGTTGATG	CGCAGCTGCCGGTTTAG	142
OPN1SW	CTTCCGCTTCAGCTCCAA	GAACCGGCTCCAGCCA	100
OPN1MW	TGGTCTCTGGCCATCATTTC	AGTACCTGCTCCAACCAAAG	160
OPN1LW	CACCTTCTTCGCATGCTTTG	TCGAAACTGCCGGTTCATAA	139
OTX2	AGGGTGCAGGTATGGTTTAAG	CGAGCTGGAGATGTCTTCTTT	116
P53	GGCCCACTTCACCGTACTAA	GTGGTTTCAAGGCCAGATGT	156
PDE6C	CGAAGAAGCCAGATCCTTATGT	TCATGTCCAGCAGTCCAATG	142
RCVRN	GCTCCTTCCAGACGATGAAA	TTGGCATCAGGCGTTCTT	193
RHO	ACCCTGGGCGGTGAAAT	ATGACCCAGGTGAAGGCAA	137
VSX2	GGAGAAGGCATTCAACGAAGCC	ACTTGGCTCGACGGTTCTGGAA	119
SOX2	CGTTCATCGACGAGGCTAAG	CCGCTCGCCATGCTATT	160
NANOG	CCCAGCTGTGTGTACTCAAT	TTCACCTGTTTGTAGCTGAGG	106
OCT4	TGGGAAGGTATTCAGCCAAAC	CTCTCACTCGGTTCTCGATACT	203
CD11B	CAAAGTGGTACGAGAGCTGTTT	CGAATGACTCCCTCTCTGTCT	150

Table S5. Single-cell RNA sequencing-based gene expression data for key photoreceptor markers in $CRX^{T155ins4/+}$ and $CRX^{K88Q/+}$ organoids in comparison to control organoids.

		$CRX^{T155ins4/+}$		$CRX^{K88Q/+}$	
**Gene**	**Localization**	**Adj P-value**	**Avg Log_2 Fold Change**	**Adj P-value**	**Avg Log_2 Fold Change**
AIPL1	Rods & Cones	$p < 1x10^{-217}$	-0.71	$p < 1x10^{-300}$	-1.67
CRX	Rods & Cones	$p < 1x10^{-126}$	0.48	$p < 1x10^{-65}$	-0.38
GUCA1B	Rods & Cones	$p < 1x10^{-300}$	-1.89	$p < 1x10^{-300}$	-2.67
GUK1	Rods & Cones	$p < 1x10^{-268}$	-0.76	$p < 1x10^{-300}$	-0.73
KCNV2	Rods & Cones	$p < 1x10^{-270}$	-1.08	$p < 1x10^{-300}$	-1.88
OTX2	Rods & Cones	$p < 1x10^{-64}$	0.59	$p < 1x10^{-202}$	0.83
PRCD	Rods & Cones	$p < 1x10^{-134}$	-0.84	$p < 1x10^{-49}$	-0.53
PROM1	Rods & Cones	$p < 1x10^{-38}$	-0.44	$p < 1x10^{-44}$	-0.50
PRPH2	Rods & Cones	$p < 1x10^{-80}$	-0.65	$p < 1x10^{-42}$	-0.50
RCVRN	Rods & Cones	$p < 1x10^{-300}$	-1.00	$p < 1x10^{-300}$	-2.31
RP1	Rods & Cones	$p < 1x10^{-62}$	-0.50	$p < 1x10^{-249}$	-0.97
GNAT1	Rods	$p < 1x10^{-198}$	-1.05	$p < 1x10^{-300}$	-1.57
GNGT1	Rods	$p < 1x10^{-300}$	-2.11	$p < 1x10^{-300}$	-2.00
NRL	Rods	$p < 1x10^{-117}$	-0.48	$p < 1x10^{-17}$	-0.19
PDE6G	Rods	$p < 1x10^{-66}$	-0.54	$p < 1x10^{-59}$	-0.50
ROM1	Rods	$p < 1x10^{-81}$	-0.62	$p < 1x10^{-288}$	-1.17
SAG	Rods	$p < 1x10^{-06}$	-0.06	$p < 1x10^{-14}$	-0.07
ARR3	Cones	$p < 1x10^{-300}$	-2.43	$p < 1x10^{-300}$	-2.93
GNAT2	Cones	$p < 1x10^{-44}$	-0.47	$p < 1x10^{-167}$	-0.87
OPN1SW	Cones	n.s.	-0.04	$p < 1x10^{-12}$	-0.08
PDE6C	Cones	$p < 1x10^{-14}$	-0.21	$p < 1x10^{-36}$	-0.28
PDE6H	Cones	$p < 1x10^{-300}$	-0.93	$p < 1x10^{-300}$	-1.20
BSN	Ribbon Synapse	n.s.	0.07	n.s.	0.04
CTBP2	Ribbon Synapse	n.s.	0.06	n.s.	-0.17
SNAP25	Synaptic Vesicles	n.s.	0.02	$p < 1x10^{-22}$	0.26
STX3	Synaptic Vesicles	$p < 1x10^{-33}$	-0.38	$p < 1x10^{-35}$	-0.40
SV2B	Synaptic Vesicles	$p < 1x10^{-22}$	-0.33	$p < 1x10^{-41}$	-0.45
MPP4	CAZ	$p < 1x10^{-27}$	-0.34	$p < 1x10^{-144}$	-0.80
RIMS2	CAZ	$p < 1x10^{-122}$	-0.78	$p < 1x10^{-261}$	-1.11
UNC119	CAZ	$p < 1x10^{-300}$	-0.91	$p < 1x10^{-300}$	-1.31
RS1	Synaptic Cleft	$p < 1x10^{-145}$	-0.84	$p < 1x10^{-216}$	-1.01

n.s. = no significance

Table S6. Raw single-cell RNA sequencing data file.
p_val = p-value; avg_log2FC = average $\log_2$ fold change; pct.1 = percentage of cells in which the gene is detected in the first group; pct.2 = percentage of cells in which the gene is detected in the second group; p_val_adjusted = adjusted p-value

Supplemental Experimental Procedures

PBMC Processing and Reprogramming

Whole blood samples were collected in heparinized tubes and processed immediately after collection. Peripheral blood mononuclear cells (PBMCs) were isolated from whole blood samples using a centrifugation method as follows. Whole blood was diluted 1:1 with 1X Dulbecco's Phosphate Buffered Saline (DPBS, Fisher Scientific, MT21031CV) without calcium or magnesium, and 7 mL of diluted blood was added on top of 3 mL of Ficoll-Paque Plus medium (VWR, 95021-205). Samples were then centrifuged for 30 minutes at 400 x g. PBMCs were then collected from the cloudy white interface between the plasma and the Ficoll-Paque. 2 x 10^6 PBMCs were plated into a single well of a 12-well tissue culture plate in Expansion Medium: StemPro-34 SFM (Fisher Scientific, 10639011), hSCF (100ng/mL; PeproTech, 300-07), FLT3L (100ng/mL; PeproTech, 300-19), IL-3 (20ng/mL; Sigma, I1646), IL-6 (20ng/mL; BioLegend, 570802). Media was changed every 2 days to fresh Expansion Medium.

For reprogramming, PBMCs were transduced using the CytoTune™-iPS 2.0 Sendai Reprogramming Kit (ThermoFisher Scientific, A16517) at an MOI of 5, based on cell number according to manufacturer instructions for lot# L2150040. Briefly, PBMCs were resuspended in fresh Expansion Medium containing the appropriate amount of virus and spun down for 30 minutes at 2000rpm before briefly resuspending cells and plating directly into a 24-well plate pre-coated with vitronectin XF (STEMCELL Technologies, 7180). After 16-18 hours, virus-containing media was replaced with fresh Expansion Medium. On day 3 post-transduction, Expansion Medium was replaced with Reprogramming Medium: StemPro-34 SFM (Fisher Scientific, 10639011), hSCF (100ng/mL; PeproTech, 300-07), FLT3L (100ng/mL; PeproTech, 300-19). Starting on day 6 post-transduction, cells were given a half medium change with mTeSR™1 medium (STEMCELL Technologies, 85850) + 0.25mM sodium butyrate (NaB; SelleckChem, S1999) every day until induced pluripotent stem cell (iPSC) colonies began to appear. NaB was removed from the media starting on day 8. Newly formed iPSC colonies were picked off the plate using a P200 pipette tip and transferred to a 6-well plate coated with Matrigel (Corning, 354234). Newly picked iPSC clones were passaged at least 10 times until a clean, fully reprogrammed clone was established and verified by immunocytochemistry and RT-PCR. Passaging was conducted by gently scraping cells off the plate after incubating cells for 2 minutes at room temperature in a solution of EDTA (1:1000; Corning, 46-034-CI) in 1X DPBS. Human iPSC colonies were maintained in mTeSR™ Plus medium (STEMCELL Technolognies, 5825) with media changes occurring every other day. The control hiPSC line (CRX^{WT}, also known as "LiPSC-ER2.2") used throughout this work was originally generated from human umbilical cord blood in a cGMP facility at Lonza Walkersville, Inc. More information on how this line was generated can be found in Baghbaderani et al., 2015.

Reverse transcription polymerase chain reaction (RT-PCR)

To confirm pluripotency in newly reprogrammed hiPSC clones, total RNA was extracted using the RNeasy Extraction Kit (Qiagen, 74104), and cDNA was then generated using the iScript cDNA Synthesis Kit (Bio-Rad, 1708891). RT-PCR using the Taq DNA Polymerase kit (ThermoFisher Scientific, EP0402) was performed to look for mRNA expression of standard pluripotency markers *SOX2*, *OCT4*, and *NANOG*, as well as loss of *CD11B* (PBMC marker) expression. The reactions were then run on a 1% agarose gel to look for the presence of a band at the appropriate size.

Thermal cycling protocol for RT-PCR:

Initial Denaturation	Denaturation	Annealing	Extension	Final Extension
95°C	95°C	56°C	72°C	72°C
3:00	0:30	0:30	0:20	5:00
	Repeat for 34 cycles			

CRX gene sequencing

The thermal cycling protocol used to amplify *CRX* for sequencing applications:

Initial Denaturation	Denaturation	Annealing	Extension	Final Extension
95°C	95°C	56°C	72°C	72°C

3:00	0:30	0:30	0:45	5:00
	Repeat for 34 cycles			

Generating an isogenic control hiPSC line

All CRISPR-Cas9-mediated gene editing in hiPSCs was performed using the following protocol. Stable hiPSC colonies were grown in a 12-well TC-treated cell culture plate in mTeSR™ Plus medium until they reached ~40-50% confluency. The culture medium was then replaced with Opti-MEM™ I Reduced Serum Medium (Gibco, 31985062) 1 hour prior to transfection. To transfect the cells, Lipofectamine^{TM} Stem Transfection Reagent (ThermoFisher Scientific, STEM00008) was used at a concentration of 1µL/500ng of total DNA, following manufacturer's instructions. To generate the isogenic control line, hiPSCs containing the *CRX*$^{K88Q/+}$ genotype were transfected with the PX459-Cas9 plasmid containing an gRNA directly targeting the K88Q mutation (5µg) along with a single-stranded oligodeoxynucleotide (ssODN) construct (synthesized by Integrated DNA Technologies) as a homology-directed repair (HDR) template (500ng). On day 2-5 post-transfection, cells were treated with puromycin (0.25µg/mL) to select for cells that took up the PX459-Cas9 plasmid. After treatment, individual cells were left to grow until they could be split to propagate individual clones. To confirm correction of the *CRX* gene, individual clones were assessed by PCR amplification followed by Sanger sequencing. Oligo sequences can be found in **Table S2**.

Retinal organoid differentiation

Colonies used to make retinal organoids were transferred to a 6-well suspension plate in a solution of 3 parts mTeSR™ Plus medium and 1 part Neural Induction Medium (NIM); this is considered "day 0" of differentiation. To continue driving the hiPSCs toward a neural fate, NIM was slowly increased each day as follows: day 0 = 3:1 of mTeSR™ Plus:NIM, day 1 = 1:1 of mTeSR™ Plus:NIM, day 2 = 1:3 of mTeSR™ Plus:NIM, day 3 = complete NIM. Media was changed to fresh NIM on day 5, followed by fresh NIM + 1.5nM BMP-4 (PeproTech, 120-05ET) on day 6. On day 7, the 3D embryoid bodies (EBs) were transferred to a Matrigel-coated plate with fresh NIM medium + 1.5nM BMP-4, to allow EBs to adhere to the plate. Adherent EBs were fed as follows: day 8 = NIM + 1.5nM BMP-4, day 9 = NIM + 0.75nM BMP-4, day 11 = NIM + 0.75nM BMP-4, day 12 = NIM + 0.375nM BMP-4, day 14 = NIM + 0.375nM BMP-4, day 15 = NIM only. From day 16 through day 29, the EBs were fed Retinal Differentiation Medium (RDM) and regions of the plate that began to develop clear retinal morphology during this time were lifted off using a P1000 pipette tip. Lifted neural retina was transferred to a suspension plate and allowed to resume a 3D organoid structure. From day 30 to day 120, the developing retinal organoids were fed 3D-RDM medium and maintained in suspension. Beyond day 120, organoids were given 3D-RDM medium without added retinoic acid (ATRA).

Media formulations for retinal differentiation protocol:

**Neural Induction Medium (NIM)**	**Manufacturer/Vendor**	**Catalog #**	**Final Concentration**
DME/F12	HyClone	SH30023.02	
MEM nonessential amino acid solution (NEAA)	Corning	25025CI	1X
GlutaMAX™ Supplement	Gibco	35050061	1X (2mM)
Heparin sulfate	Sigma	H3393	2µg/mL
Penicillin/Streptomycin	Corning	30001CI	1%
N-2 Supplement	Gibco	17502001	1X

**Retinal Differentiation Medium (RDM)**	**Manufacturer/Vendor**	**Catalog #**	**Final Concentration**
DME/F12	HyClone	SH30023.02	1 part
DMEM High Glucose	HyClone	SH30022.02	1 part
MEM nonessential amino acid solution (NEAA)	Corning	25025CI	1X
GlutaMAX™ Supplement	Gibco	35050061	1X (2mM)
Penicillin/Streptomycin	Corning	30001CI	1%
B-27™ Supplement	Gibco	17504001	1X

**3D-Retinal Differentiation Medium (3D-RDM)**	**Manufacturer/Vendor**	**Catalog #**	**Final Concentration**
DME/F12	HyClone	SH30023.02	1 part
DMEM High Glucose	HyClone	SH30022.02	1 part

Fetal Bovine Serum	R&D	S11150	5%
GlutaMAX™ Supplement	Gibco	35050061	1X (2mM)
Penicillin/Streptomycin	Corning	30001CI	1%
Taurine	Sigma	T0625	200μM
Chemically Defined Lipid Concentrate	Gibco	11905031	1:1000
B-27™ Supplement	Gibco	17504001	1X
Retinoic acid (ATRA)	Sigma	R2625	1μM

Immunocytochemistry

Each newly reprogrammed hiPSC clone was grown on a 24-well TC-treated cell culture plate to reach ~50-70% confluency. The cells were then fixed on the plate for 10 minutes at room temperature using 4% PFA prior to staining. After rinsing three times with 1X PBS, non-specific binding was blocked using 10% normal donkey serum (NDS; MilliporeSigma, S30-100ML) in 1X PBS with 0.1% Triton X-100 (VWR, 0694) for 15 minutes at room temperature. The cells were then incubated for 1 hour at room temperature with primary antibodies targeting SOX2, NANOG, and OCT4 (see **Table S3** for full list of antibodies). After washing cells three times with 1X PBS, a secondary antibody solution was added to the cells for 30 minutes at room temperature, protected from light. Finally, cells were counterstained with 4′,6-diamidino-2-phenylindole (DAPI, 1μg/mL; Roche, 10236276001) for 2 minutes, before being imaged on an Olympus BX51 microscope in 1X PBS.

Immunofluorescence

Retinal organoids were collected at D75, D90, D120, D150, D180, and D240 (n=3/clone per collection, with 3 separate collections per time point) for each genotype. Organoids were fixed for 30 minutes in 4% PFA, followed by exposure to a series of sucrose solutions in 1X PBS: 10% sucrose for 15 minutes, 15% sucrose for 30 minutes, then 20% sucrose for 3 hours. Organoids were then flash frozen in a 2:1 mixture of 20% sucrose and Optimal Cutting Temperature compound (O.C.T.; VWR, 25608-930). 7μm-thick sections were collected from the cryoblocks for immunofluorescence staining. Sections were incubated in 10% NDS for 15 minutes, followed by a 2-hour incubation with primary antibodies at room temperature (see **Table S3** for full list of antibodies). After rinsing three times with 1X PBS, sections were incubated with the corresponding secondary antibodies for 30 minutes in the dark at room temperature. DAPI solution (1μg/mL) was then applied to each section for 2 minutes in the dark, before rinsing sections three times with 1X PBS. Finally, a coverslip was applied to each slide using Fluoromount-G™ Slide Mounting Medium (Electron Microscopy Sciences, 17984-25). Confocal images were captured using an LSM700 microscope with a 40X lens.

Quantitative real-time PCR (qPCR) analysis

For qPCR experiments, the following thermal cycling protocol was used on a Bio-Rad CFX Connect Real-Time PCR System:

Polymerase activation & initial denaturation	Denaturation	Annealing/ Extension	Melt Curve Analysis
95°C	95°C	58°C	65°C-95°C at 0.5°C increments
0:30	0:10	0:30	0:05/step
	Repeat for 39 cycles		

Fluorescence Intensity analysis

Fluorescence intensity measurements were collected for n=3 organoids per marker per line using FIJI. Measurements were first normalized to DAPI intensity measurements, and then normalized to control. All data summary graphs were made using GraphPad Prism 8, and all statistical analyses were performed via one-way ANOVA with a Dunnett test to correct for multiple comparisons.

Resource Availability

Further information and requests for resources and reagents should be directed to and will be fulfilled by the Corresponding Author, Deepak Lamba (Deepak.Lamba@ucsf.edu). All unique/stable reagents generated in this study are available from Dr. Lamba with a completed Materials Transfer Agreement. The published article includes all scRNAseq datasets generated or analyzed during this study.

Supplemental References

Baghbaderani, B.A., Tian, X., Neo, B.H., Burkall, A., Dimezzo, T., Sierra, G., Zeng, X., Warren, K., Kovarcik, D.P., Fellner, T., et al. (2015). cGMP-Manufactured Human Induced Pluripotent Stem Cells Are Available for Pre-clinical and Clinical Applications. Stem Cell Reports *5*, 647–659. 10.1016/j.stemcr.2015.08.015

Cao, J., Spielmann, M., Qiu, X., Huang, X., Ibrahim, D.M., Hill, A.J., Zhang, F., Mundlos, S., Christiansen, L., Steemers, F.J., et al. (2019). The single-cell transcriptional landscape of mammalian organogenesis. Nature *566*, 496–502. 10.1038/s41586-019-0969-x

Haghverdi, L., Lun, A.T.L., Morgan, M.D., and Marioni, J.C. (2018). Batch effects in single-cell RNA-sequencing data are corrected by matching mutual nearest neighbors. Nat Biotechnol *36*, 421–427. 10.1038/nbt.4091

Stuart, T., Butler, A., Hoffman, P., Hafemeister, C., Papalexi, E., Mauck, W.M., Hao, Y., Stoeckius, M., Smibert, P., and Satija, R. (2019). Comprehensive Integration of Single-Cell Data. Cell *177*, 1888-1902.e21. 10.1016/j.cell.2019.05.031

Yousif, A., Drou, N., Rowe, J., Khalfan, M., and Gunsalus, K.C. (2020). NASQAR: a web-based platform for high-throughput sequencing data analysis and visualization. BMC Bioinformatics *21*, 267. 10.1186/s12859-020-03577-4
